# BacPE: a versatile prime-editing platform in bacteria by inhibiting DNA exonucleases

**DOI:** 10.1038/s41467-024-45114-4

**Published:** 2024-01-27

**Authors:** Hongyuan Zhang, Jiacheng Ma, Zhaowei Wu, Xiaoyang Chen, Yangyang Qian, Weizhong Chen, Zhipeng Wang, Ya Zhang, Huanhu Zhu, Xingxu Huang, Quanjiang Ji

**Affiliations:** 1 School of Physical Science and Technology & State Key Laboratory of Advanced Medical Materials and Devices, ShanghaiTech University, Shanghai, 201210 China; 2 School of Life Science and Technology, ShanghaiTech University, Shanghai, 201210 China; 3 School of Marine Sciences, Ningbo University, Ningbo, Zhejiang 315832 China; 4 Zhejiang Lab, Hangzhou, Zhejiang 311121 China; 5 Gene Editing Center, School of Life Science and Technology, ShanghaiTech University, Shanghai, 201210 China; 6 Shanghai Clinical Research and Trial Center, Shanghai, 201210 China

**Keywords:** Microbial genetics, Genetic engineering, CRISPR-Cas9 genome editing

## Abstract

Prime editing allows precise installation of any single base substitution and small insertions and deletions without requiring homologous recombination or double-strand DNA breaks in eukaryotic cells. However, the applications in bacteria are hindered and the underlying mechanisms that impede efficient prime editing remain enigmatic. Here, we report the determination of vital cellular factors that affect prime editing in bacteria. Genetic screening of 129 *Escherichia coli* transposon mutants identified *sbcB*, a 3ʹ→5ʹ DNA exonuclease, as a key genetic determinant in impeding prime editing in *E. coli*, combinational deletions of which with two additional 3ʹ→5ʹ DNA exonucleases, *xseA* and *exoX*, drastically enhanced the prime editing efficiency by up to 100-fold. Efficient prime editing in wild-type *E. coli* can be achieved by simultaneously inhibiting the DNA exonucleases via CRISPRi. Our results pave the way for versatile applications of prime editing for bacterial genome engineering.

## Abstract (web summary)

Prime editing in bacteria is currently inefficient. Here the authors report BacPE, a versatile prime editing platform in *Escherichia coli* that works by inhibiting 3′→5′ DNA exonucleases, highlighting the intrinsic genetic factors that are adverse to efficient prime editing.

## Introduction

Prime editors can mediate DNA base pair substitutions, small insertions and deletions without introducing double-strand breaks or requiring homologous recombination^1,2^. Recently, strategies of twin prime editors that target long-distance enable large deletions^2–6^ (<10 kb), and a combination of prime editing and site-specific serine integrase achieves large-size DNA insertion^2,7^, and thereby prime editors show promising potential for genome engineering in all kingdoms of life. Prime editors minimally comprise an engineered reverse transcriptase (RT)-Cas9 nickase fusion protein (PE2) and a prime editing guide RNA (pegRNA) that contain a spacer sequence for DNA targeting and a 3ʹ extension containing the desired edits^1^. The prime editing machinery binds to a target site via base pairing with the spacer of the pegRNA and nicks the non-target strand to expose a DNA 3′ end. This exposed 3′ end hybridizes to the primer binding site (PBS) of the pegRNA to initiate the reverse transcription reaction with the engineered RT and synthesize the desired edits from the RT template. Subsequent flap equilibration, 5′ flap cleavage, and DNA repair processes enable the incorporation of the 3′ DNA flap that contains the desired edits into the target genomic site. The PE3 system is distinguished from the PE2 system by harboring an additional sgRNA that nicks the non-edited strand with enhanced editing efficiency by facilitating the favorable DNA repair pathway^1^.

Prime editors have been widely applied for versatile genome engineering in a variety of eukaryotic cells, such as human^1,8,9^, mice^10^, rice and wheat^11^, zebrafish^12^, and *Drosophila*^13^. However, applying prime editors in prokaryotes is limited to *E. coli,* and the editing activities are at low levels, restricting the practical applications of the prime editing system for bacterial genome engineering^14^. Encouraged by the success in identifying the DNA mismatch repair (MMR) pathway that impedes prime editing in human cells and the subsequent improvements of editing efficiency via the inhibition of this^8,15^, we sought to determine the key genetic determinants that restrict efficient prime editing in bacteria and establish a versatile prokaryotic prime editing platform.

In this study, through comparative prime editing in different bacterial species and genetic screening approaches, we identify that 3′→5′ DNA exonucleases are key genetic factors in impeding prime editing in bacteria, which are strikingly different from the MMR strategy employed by human cells for prime editing inhibition. We further show that deletion or inhibition of those 3′→5′ DNA exonucleases can drastically enhance prime editing efficiencies in bacteria. We propose a 3′-directed hydrolysis model for inhibiting prime editing via degradation of the prime editing intermediates by the 3′→5′ DNA exonucleases and demonstrate that the 3′-directed hydrolysis mechanism is conserved in other bacterial species. Our results uncover the exceptional prime editing inhibition mechanism and pave the way for the versatile application of prime editors for genome engineering in bacteria.

## Results

### Striking editing efficiency differences in distinct bacteria with prime editing

**Fig. 1:**
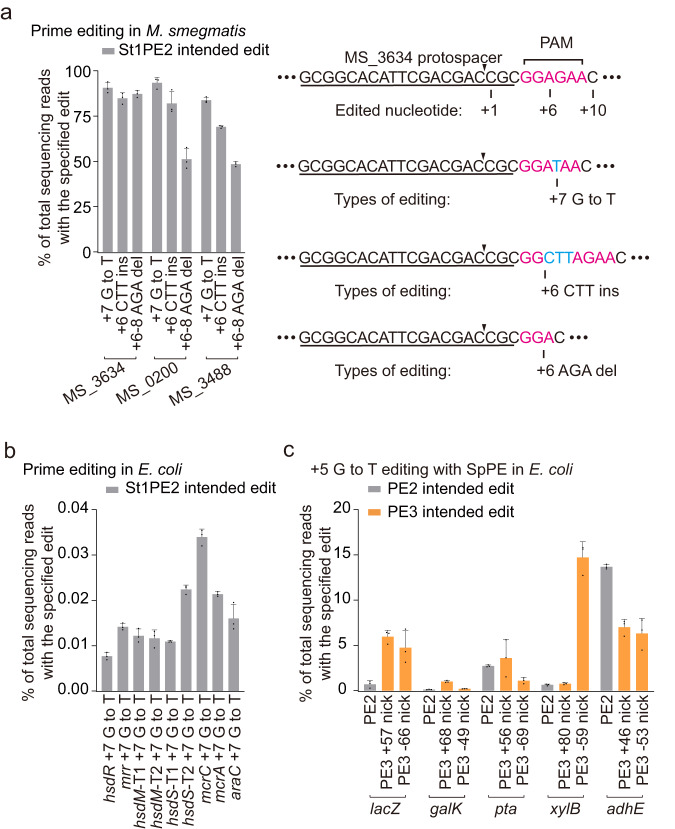
Prime-editing frequencies in *M. smegmatis* and *E. coli*. **a** Prime editing with St1PE2 in *M*. *smegmatis*. PAM sequence is colored pink, and the edited nucleotide is colored blue. The black triangle marks the nick site introduced by PE. Data represent mean ± s.d. of *n* = 3 independent replicates. **b** Prime editing with St1PE2 in *E. coli*. Data represent mean ± s.d. of *n* = 3 independent replicates. **c** Prime editing with SpPE in *E. coli*. The PE3 system nicks the non-edited strand to enhance prime editing. Data represent mean ± s.d. of *n* = 3 independent replicates.

Previously, we established a mycobacterial base editing platform using a *Streptococcus thermophilus* Cas9 (St1Cas9)-deaminase fusion that allows C-to-T or C-to-G conversions^16^. We sought to expand the editing versatility to mycobacteria by developing a prime editing platform. We constructed a prime editing system that harbors an St1PE2 fusion protein (*S. thermophilus* Cas9 H599A nickase [nSt1Cas9] fused to an engineered reverse transcriptase [ERT]) under an anhydrotetracycline (ATc)-inducible promoter^17^ and a cognate pegRNA under the control of another ATc-inducible promoter (Supplementary Fig. 1). We designed nine pegRNAs to target three different genes in *M. smegmatis* and determined the prime editing efficiency using deep amplicon sequencing. The St1PE2 system achieved the desired point mutations, insertions, and deletions with an editing efficiency of 45–90% at the target sites (Fig. 1a), showing that St1PE2 is an efficient prime editor in *M. smegmatis*. The editing efficiencies of St1PE2 varied with PBS length and RT template length, with the optimal lengths being 9–15 and 18 nucleotides, respectively (Supplementary Fig. 2).

To examine the universality of the St1PE2 system, we assessed the prime editing efficiency in *E. coli* using the same St1PE2 system as demonstrated in *M. smegmatis*. However, St1PE2 only achieved minimal editing efficiencies with a maximal efficiency of <0.1% among the nine targeted sites in *E. coli* (Fig. 1b). As St1Cas9 may not be an optimal Cas9 nuclease for *E. coli*, we constructed a SpCas9-based prime editing system that harbors an SpPE2 fusion protein^1^ (*Streptococcus pyogenes* Cas9 H840A nickase [nSpCas9] fused to an ERT) and a cognate pegRNA (Supplementary Fig. 3). SpPE2 resulted in only minimal editing efficiencies irrespective of the editing types (Fig. 1c, Supplementary Fig. 4a, b). We then applied the SpPE3 system that harbors an additional sgRNA to nick the non-edited strand to improve the editing efficiency. This moderately improved the editing efficiency at several of the targeted sites (Fig. 1c, Supplementary Fig. 4a, b), but the overall editing efficiency remains a low level ranging from 0 to 15%, suggesting that other intrinsic genetic factors may exist in limiting prime editing in *E. coli*.

### 3′→5′ ssDNA exonucleases are key genetic factors in impeding prime editing in *E. coli*

Recent studies demonstrated that the DNA mismatch repair (MMR) pathway is a major genetic factor in inhibiting prime editing in human cells^8,15^. MutS, an essential protein in MMR^18^, recognizes mismatched base pairs and initiates downstream pathways that repair the introduced mismatched bases, reducing the editing efficiency (Supplementary Fig. 5). To determine whether MMR also inhibits prime editing in *E. coli*, we examined the editing efficiency of the SpPE2 system in both wild-type *E. coli* MG1655 and an MMR-deficient MG1655 strain that carries a deletion of *mutS*. In most cases (40/45), deletion of *mutS* had minimal or no impact on the improvement of prime editing efficiency (Supplementary Fig. 6a–c), indicating that the key limiting factor of prime editing in *E. coli* is distinct from that in human cells.

**Fig. 2:**
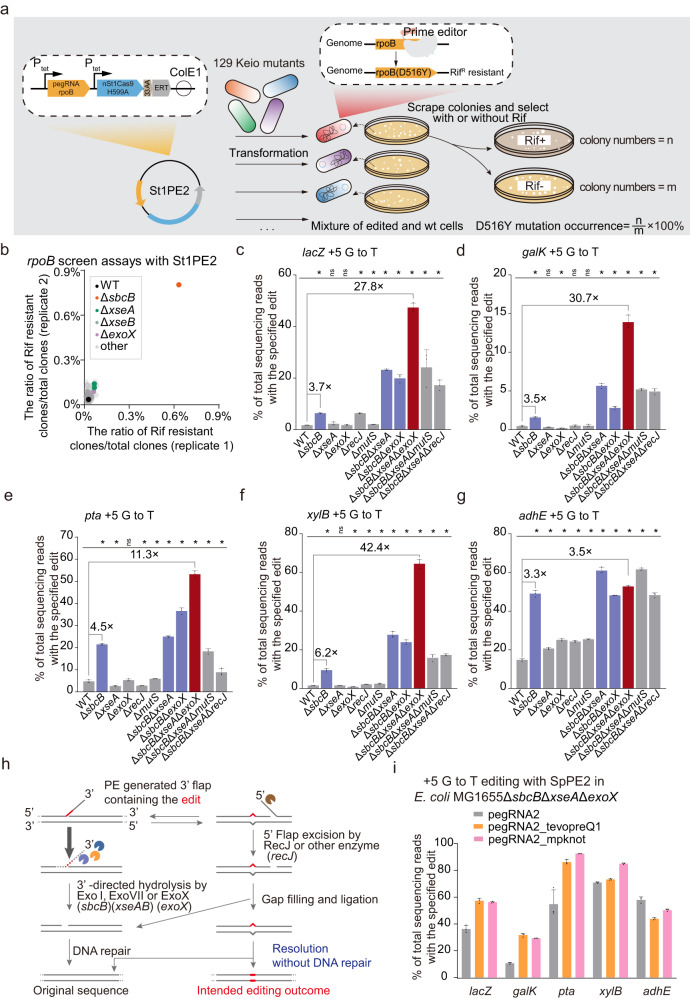
Identification of key genetic determinates that impede prime editing in *E. coli*. **a** Schematic of the *rpoB*-based genetic screening approach. 129 Keio mutants were individually transformed with St1PE2 to enable the conversion of D516Y mutation in *rpoB*. The ratio of (Rif resistant cononies)/(total colonies) was calculated by the number of colonies on Rif+ plate divided by the number of colonies on Rif- plate. **b** Identification of the key genes that inhibit prime editing using the *rpoB*-based screening assay. All values from *n* = 2 independent replicates are shown. **c**–**g** Comparison of the prime editing efficiency in *E. coli* MG1655 at different targeting loci *lacZ* (**c**), *galK* (**d**), *pta* (**e**), *xylB* (**f**), and *adhE* (**g**). Δ*sbcB*, Δ*sbcB*Δ*xseA*, and Δ*sbcB*Δ*exoX* mutants are colored blue, Δ*sbcB*Δ*xseA*Δ*exoX* mutants are colored red. The black stars represent the statistical differences between WT and mutants, respectively. Two-tailed student’s *t* test was performed. *p < 0.05. Data represent mean ± s.d. of *n* = 3 independent replicates. **h** The 3′-directed hydrolysis model that inhibits prime editing in *E. coli*. The prime editing intermediate was digested by exonucleases. **i** Impact of 3′ RNA structural motif on prime editing efficiency. Data represent mean ± s.d. of *n* = 3 independent replicates.

To identify the vital genetic factors that restrict prime editing in *E. coli*, we performed *rpoB*-based genetic screening with 129 *E. coli* transposon mutants^19^ of potential DNA repair-related genes (Fig. 2a). The St1PE2 system was introduced into the transposon mutants to produce a D516Y mutation in *rpoB*, the successful editing of which would enable bacterial survival in the presence of rifampin (Rif) (Supplementary Fig. 7). Therefore, the editing efficiency for the D516Y mutation could be indicated by the colony counts on Rif+ plates divided by the colony counts on the Rif− plates. We observed that *sbcB* emerged as the top hit in the screens, and the inactivation of *sbcB* (ExoI), a 3′→5′ ssDNA exonuclease, significantly enhanced the prime editing efficiency, whereas the individual inactivation of the other 128 genes had minimal or no impact (Fig. 2b). Furthermore, we systematically and quantitatively compared the editing efficiencies of SpPE2 in the wild-type MG1655 strain and the *sbcB*-clean-deletion mutant across different genomic loci and distinct editing types, showing that deletion of *sbcB* moderately improved prime editing efficiency with up to 9-fold (Fig. 2c–g, Supplementary Fig. 8).

Given the limited overall editing efficiency of SpPE2 in the *sbcB* mutant and the presence of other DNA exonucleases in *E. coli* (Fig. 2c–g, Supplementary Fig. 8), we reasoned that several of those DNA exonucleases may also inhibit prime editing, but in a redundant manner when *sbcB* is present. Consequently, we performed individual gene deletion of all the potential DNA nucleases^20^ in wild-type MG1655 and the *sbcB*-deletion mutant and assessed the editing efficiencies of SpPE2 in these strains. The deletion of *xseA* (a catalytic subunit of ExoVII) or *exoX*, two additional 3′→5′ ssDNA exonucleases, in the *sbcB* mutant, substantially enhanced the editing efficiencies (Fig. 2c–g, Supplementary Fig. 8), whereas the deletion of these and other potential DNA nucleases in the wild-type MG1655 did not improve the editing efficiency with SpPE2. Except at the *xylB* loci, the additional deletion of other potential 3′→5′ DNA exonuleases^20^ in the *sbcB* mutant did not enhance the editing efficiency (Supplementary Fig. 9a). Moreover, combinational deletions of all three 3′→5′ ssDNA exonucleases *sbcB*, *xseA*, and *exoX*, drastically increased editing efficiency up to 42.4-fold for +5 G to T conversion editing (Fig. 2c–g), 99.5-fold for +5 TTAA insertion editing (Supplementary Fig. 8), and 69.5-fold for +4–6 CGG deletion editing (Supplementary Fig. 8). Furthermore, we deleted *mutS* or *recJ* (a 5′→3′ ssDNA exonuclease) in *E. coli* MG1655Δ*sbcB*Δ*xseA*. The additional deletion of *mutS* had minimal or no impact on the prime editing efficiency, whereas the additional deletion of *recJ* decreased the prime editing efficiency across most of the edits (Fig. 2c–g, Supplementary Fig. 8).

### 3′-directed hydrolysis model for inhibiting prime editing

Consequently, we propose a 3′-directed hydrolysis model for inhibiting prime editing in *E. coli* by degrading the prime editing intermediates with the 3′→5′ ssDNA exonucleases (Fig. 2h). We hypothesize that the 3′ DNA flap generated by the prime editor would be efficiently degraded by DNA exonucleases that possess the 3′→5′ ssDNA exonuclease activity, thereby regenerating the original sequence through subsequent DNA repair processes. Therefore, deletion of the 3′→5′ ssDNA exonucleases would facilitate flap equilibration to generate the 5′ flap that would be further degraded by RecJ, enabling the incorporation of the desired edits with the subsequent DNA ligation and repair pathways.

Previous studies indicated that *sbcB* (ExoI) degrades DNA at a rate of up to 10,000 nucleotides/min^21^, substantially faster than that of *exoX* (ExoX), which degrades DNA with a rate of up to 1400 nucleotides/min^22^. We cannot find the substrate degradation rate of *xseAB* (ExoVII). Thereby, we performed the cleavage assay to compare the degradation activity of ExoI and ExoVII on PE intermediates. PE intermediates were produced by annealing the oligonucleotides depicted in Supplementary Fig. 9b, further digested with ExoI or ExoVII, and analyzed by denaturing Urea PAGE. The results showed that both ExoI and ExoVII could degrade the FAM-labeled DNA, but the catalytic rate of ExoI was faster than that of ExoVII, consistent with the notion that ExoI plays a primary role in PE inhibition. A ~20 nt DNA product could be observed in the degradation assay, and prolonged incubation could result in oligonucleotides shorter than 10 nt, suggesting that both nucleases could also degrade dsDNA.

Enhancing pegRNA stability is also an effective approach to improve prime editing in human cells^23–26^. To improve the editing efficiency, we incorporated different RNA structural motifs into the 3′ terminus of pegRNA and compared the editing efficiencies of these engineered pegRNAs with those of the original pegRNA. The incorporation of tevopreQ1 or mpknot improved the editing efficiency at most of the targeted sites by up to 3-fold in *E. coli* MG1655Δ*sbcB*Δ*xseA*Δ*exoX* (Fig. 2i).

### Efficient prime editing in *E. coli* by inhibiting 3′→5′ ssDNA exonucleases

**Fig. 3:**
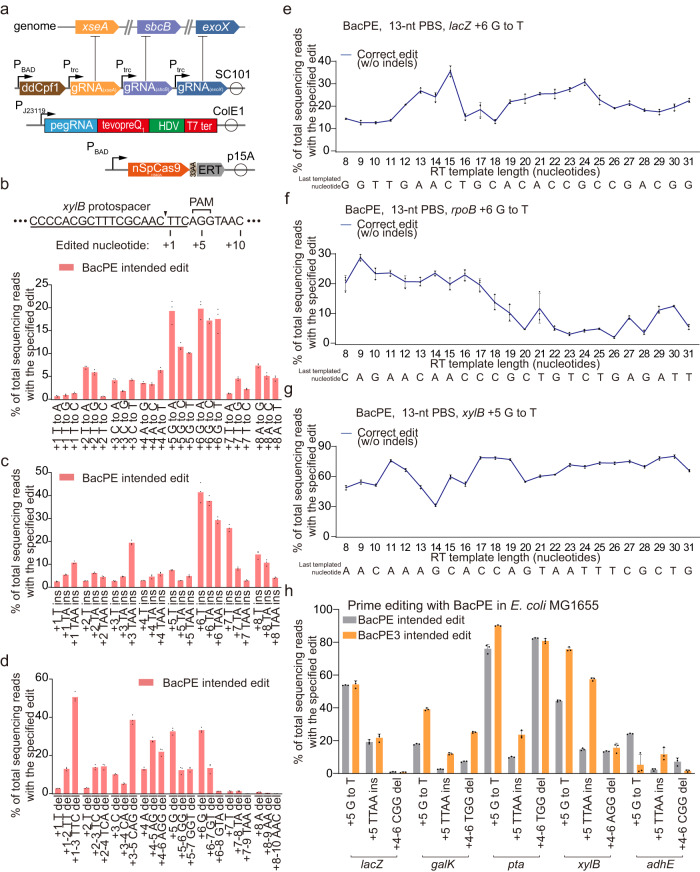
Characterizations of the BacPE system across diverse editing types. **a** Schematic of the BacPE system. Key genes that restrict prime editing were knocked down using CRISPRi prior to prime editing. PE effector protein and pegRNA were encoded in different plasmids. **b** Base substitution editing with BacPE at the *xylB* locus in *E. coli* MG1655. The epegRNA nick site is marked with a black triangle. Data represent mean ± s.d. of *n* = 3 independent replicates. **c** Insertion editing with BacPE at the *xylB* locus in *E. coli* MG1655. Data represent mean ± s.d. of *n* = 3 independent replicates. **d** Deletion editing with BacPE system at the *xylB* locus in *E. coli* MG1655. Data represent mean ± s.d. of n = 3 independent replicates. **e**–**g** Effect of RT template on BacPE-mediated base transversion editing at *lacZ* (**e**), *rpoB* (**f**), and *xylB* (**g**) loci. The sequence below shows the last templated nucleotide. Data represent mean ± s.d. of *n* = 3 independent replicates. **h** Prime editing with the BacPE3 system in *E. coli* MG1655. The BacPE3 system harbors an additional gRNA to nick the non-edited strand. Data represent mean ± s.d. of *n* = 3 independent replicates.

Next, we assessed whether repressing the three 3′→5′ ssDNA exonucleases via a Cas12a-based CRISPRi system^27^ could achieve efficient prime editing in wild-type *E. coli*. We obtained three different gRNAs that individually inhibited the transcription of the three exonucleases with approximately 60-90% repression efficiency (Supplementary Fig. 10). We designed a prime editing platform in *E. coli* termed BacPE that simultaneously inhibited the transcription of *sbcB*, *xseA*, and *exoX* and used the SpPE2 system for editing (Fig. 3a). We then systematically characterized the editing efficiency of BacPE in wild-type MG1655 across different editing types. Among all base substitution edits at the *xylB* locus, the editing efficiencies varied substantially at different editing positions from 0.59% to 19.79% with the maximum efficiency for the edits that alter the protospacer adjacent motif (PAM) sequence (Fig. 3b), consistent with previous findings^1^. The insertion and deletion editing efficiencies also varied substantially at different editing positions, with the maximum efficiency for the edits occurring at PAM or the seed region of the SpCas9 spacer (Fig. 3c, d). Furthermore, we investigated the effect of RT template length and last template nucleotide type on the editing efficiency. Editing efficiency was substantially reduced when the RT template was >18 nucleotides at the *rpoB* locus but not at the *lacZ* or *xylB* locus, where the RT template length did not drastically affect the editing efficiency (Fig. 3e–g). The editing efficiency was generally lower when the last template nucleotide was a G at the *rpoB* and *xylB* loci, but not at the *lacZ* locus (Fig. 3e–g). Collectively, these results indicate that the editing efficiency could be significantly affected by differences in editing positions and types, length of RT template, and type of nucleotide in the final position in the template. Although additional nicking at the non-edited strand with BacPE3 improved the prime editing efficiency between 1.01- and 5.19-fold across 11 of 15 tested sites, this nicking impeded prime editing at several of the targeted loci (Fig. 3h).

### The 3′-directed hydrolysis mechanism is conserved in different bacteria

**Fig. 4:**
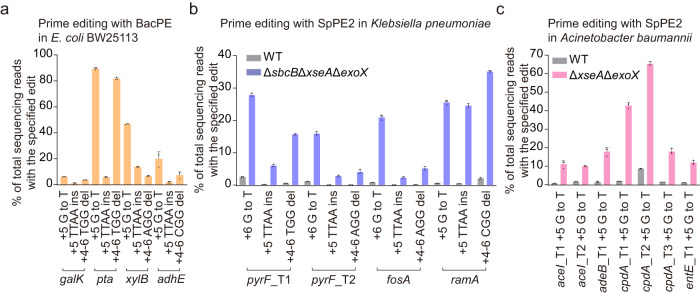
Prime editing in different bacteria. **a** Prime editing in *E. coli* BW25113 using BacPE. Data represent mean ± s.d. of *n* = 3 independent replicates. **b** Prime editing with the SpPE2 system in *K. pneumoniae* 1.6366 and the Δ*sbcB*Δ*xseA*Δ*exoX* mutant strain. Data represent mean ± s.d. of *n* = 3 independent replicates. **c** Prime editing with the SpPE2 system in *A. baumannii* ATCC17978 and the Δ*xseA*Δ*exoX* mutant strain. Data represent mean ± s.d. of *n* = 3 independent replicates.

Characterization of the editing efficiency of BacPE in *E. coli* BW25113 showed that the editing efficiencies varied from 1% to 89.4% at different sites and with different editing types (Fig. 4a). To assess whether the 3′-directed hydrolysis model is a conserved mechanism in impeding prime editing in other bacterial species, we deleted the three 3′→5′ ssDNA exonulceases in *Klebsiella pneumoniae* strain 1.6366. The editing efficiency of SpPE2 in this mutant was increased between 11- and 70-fold compared with that in the wild-type strain when *sbcB*, *xseA*, and *exoX* were simultaneously deleted (Fig. 4b). In addition, we simultaneously deleted *xseA* and *exoX* in *Acinetobacter baumannii* ATCC17978 that lacks the *sbcB* gene and observed a similar enhancement of the editing efficiency after deletion (Fig. 4c). Collectively, these results indicate that the 3′-directed hydrolysis model is a conserved mechanism in restricting prime editing in diverse bacterial species.

## Discussion

In summary, we used a genetic screening approach to identify that *sbcB* is a key genetic factor in impeding prime editing in *E. coli*. Despite the substantial enhancement of the editing efficiency after the deletion of *sbcB*, the overall editing efficiencies remain <25% at most targeted loci. Two other 3′→5′ ssDNA exonucleases, *xseA* and *exoX*, play important roles in restricting prime editing when *sbcB* is absent. Simultaneous deletion of *sbcB*, *xseA*, and *exoX* increased the editing efficiency by up to 100-fold. Thus, we propose a 3′-directed hydrolysis model whereby the prime editing intermediate is efficiently degraded by 3′→5′ ssDNA exonucleases to attenuate the editing efficiency. Furthermore, the deletion of *recJ*, a 5′→3′ ssDNA exonuclease for 5′ flap cleavage, reduced the prime editing efficiency, consistent with the previous hypothesis that the 5′ flap excision process is necessary for prime editing^1^.

Our findings highlight the importance of intrinsic pathways and redundant genes on prime editing restriction, and might provide insights on improving the editing efficiencies of other genome editing tools. Nuclease-based genome editing methods rely on intrinsic or exogenous homologous recombination (HR) systems for DSB repair^28,29^. Modulation of cellular pathways that can enhance HR capacity might improve the editing efficiencies of those technologies. CRISPR-Cas12k-associated transposons (CASTs) can mediate site-specific DNA insertion in bacteria^30^. However, the DNA-insertion efficiencies vary substantially across different bacterial species^31^. The discovery of the cellular determinants would also improve the editing efficiency of CRISPR-Cas12k-mediated DNA insertion.

The key genetic mechanism that impedes prime editing in bacteria is strikingly different from that in human cells, where the MMR pathway, but not the 3′→5′ ssDNA exonucleases, is primarily involved in prime editing inhibition^8,15^, suggesting that different mechanisms in prime editing restriction may exist in different organisms. Intriguingly, unlike that in *E. coli*, *K. pneumoniae*, or *A. baumannii*, highly efficient prime editing was readily achieved in *M. smegmatis* without the perturbation of the cellular genetic pathways. Many factors, including the slow growth that enables the continuous accumulation of the editing products and the absence of the key 3′→5′ ssDNA exonuclease *sbcB*, may contribute to the high editing efficiency in *M. smegmatis*. We notice that the prime editing efficiency varies substantially across different editing types and editing loci. Future efforts are required to systematically profile the editing efficiencies in a high-throughput manner in bacteria to enable the effective design of optimal pegRNAs as that in human cells^32–34^.

Compared with nuclease-based genome editing methods that rely on HR for precise editing, prime editors achieve the installation of any single base substitution and small insertions and deletions without requiring homologous recombination or double-strand DNA breaks, potentiating the editing in bacterial species that lack a strong HR system. The developed BacPE platform provides a template for prime editing system development in diverse bacterial species and, therefore, is highly valuable for bacterial genome engineering, and paves the way for direct engineering and improvement of prime editors in bacteria.

## Methods

### Bacterial strains, plasmids, primers and culture conditions

The strains, plasmids, and primers used in this study are listed in Supplementary Tables 1–3, respectively. *M. smegmatis* was grown in MiddleBrook 7H9 broth (ELITE-MEDIA) supplemented with 10% ADC enrichment, 0.05% Tween 80 (Solarbio) and 0.2% glycerol. *E. coli*, *K. pneumoniae* and *A. baumannii* were grown in Terrific Broth (TB). Antibiotics and inducers were used at the following concentrations: kanamycin (20 μg/mL for *M. smegmatis*, 100 μg/mL for other bacteria), carbenicillin (100 μg/mL), apramycin (100 μg/mL), chloramphenicol (50 μg/mL), arabinose (10 mM), IPTG (0.5 mM), and ATc (100 ng/mL for *M. smegmatis*, 1 μg/mL for *E. coli*).

### Plasmid construction

To construct the St1PE2 system, the St1Cas9-RT gene and the pegRNA were assembled into a pLJR962^17^-based plasmid by Gibson assembly. To construct the SpPE2 system for editing in *E. coli* and *K. pneumoniae*, the SpCas9-RT gene was PCR amplified, digested with Dpn1 (ABclonal) and cloned into a p15a-based plasmid with an *araBAD* promoter and the pegRNA was cloned into a pSGAb^29^-based plasmid with a J23119 promoter. To construct the SpPE2 system for editing in *A. baumannii*, the SpCas9-RT gene was cloned into a pBECAb^29^-based plasmid, and the pegRNA plasmid is the same as that in *K. pneumoniae*. To simultaneously inhibit the transcription of *sbcB*, *xseA* and *exoX* in *E. coli*, crRNAs targeting these genes were cloned into a CRISPRi^27^ plasmid. Targeting spacers were cloned to pegRNA plasmids by Golden Gate assembly (Supplementary Note 1). Colonies were cultured in LB broth, and plasmids were extracted using MolPure Plasmid Mini Kit (Yeasen).

### Strain construction

The *E. coli* mutant strains were constructed by using the pKD46-Cas9/pCRISPR system^35^ following the procedure described previously^35,36^. In brief, a targeting spacer was cloned into the pCRISPR plasmid, and the resulting plasmid and a corresponding ssDNA repair template were co-electroporated into *E. coli* MG1655 containing the pKD46-Cas9-RecA-Cure plasmid. The target site was amplified using Easy Taq (TransGen Biotech), and 1 μL PCR products and 3 μL Spark 1Kb Plus DNA Marker (Shandong Sparkjade Biotechnology Co., Ltd.) were analyzed using agarose gel. After verifying gene deletion using Sanger sequencing, the successfully edited mutants were subjected to plasmid curing. The *K. pneumoniae* mutant strains were constructed by using the pCasKP/pSGKP system^28^. In brief, a targeting spacer was cloned into the pSGKP plasmid. The constructed pSGKP plasmid and a corresponding ssDNA repair template were co-electroporated into *K. pneumoniae* 1.6366 containing pCasKP. After verifying gene deletion using Sanger sequencing, the successfully edited mutants were subjected to plasmid curing. The *A. baumannii* mutant strains were constructed by using the pBECAb system^29^. In brief, a targeting spacer was cloned into the pBECAb plasmid, and the resulting plasmid was electroporated into *A. baumannii* ATCC17978 to generate a premature stop codon. After verifying gene inactivation using Sanger sequencing, the successfully edited mutants were subjected to plasmid curing. Primers for strain construction were listed in Supplementary Table S3.

### Prime editing

For prime editing with the St1PE2 system, the editing plasmid containing the St1PE2 fusion protein and the pegRNA was electroporated into *M. smegmatis* or *E. coli* and incubated at 37 °C for different times (4 days for *M. smegmatis* and 18 hours for *E. coli*) before editing efficiency determination using deep amplicon sequencing. For prime editing with the SpPE2 system, the pegRNA plasmid was electroporated into *E. coli*, *K. pneumoniae*, or *A. baumannii* containing the SpPE2 fusion protein plasmid and incubated at 37 °C for 18 hours before editing efficiency determination using deep amplicon sequencing. For prime editing with the BacPE system, 1 mL *E. coli* cells carrying the SpPE2 plasmid and the CRISPRi plasmid were cultured into 100 mL TB supplemented with 100 μg/mL carbenicillin, 50 μg/mL chloramphenicol, 0.5 mM IPTG, and 10 mM arabinose. When OD_600_ reached 0.5, the cell cultures were placed on ice for 10 min, followed by centrifugation at 4000 × *g*. The pellets were washed three times with 10% ice-cold glycerol and resuspended in 1 mL 10% ice-cold glycerol. For electroporation, 50 ng pegRNA plasmids were mixed with 50 μL competent cells. The cells were electroporated using 1 mm cuvette at 1800 V and recovered in 1 mL TB at 30 °C for 1 hour before being plated onto a TB agar plate supplemented with 100 μg/mL carbenicillin, 50 μg/mL chloramphenicol, 100 μg/mL kanamycin, 0.5 mM IPTG, and 10 mM arabinose. The plate was incubated at 30 °C for 36 hours before editing efficiency determination using deep amplicon sequencing.

### Genetic screening assay

The St1PE2 prime editing plasmid that targets *rpoB* was electroporated into different *E. coli* transposon mutants^19^, and the cell cultures were recovered in 1 mL TB at 37 °C for 1 hour before being plated onto a TB agar plate supplemented with 100 μg/mL carbenicillin and 1 μg/mL ATc. After incubation at 37 °C for 18 hours, all colonies were collected and plated onto two individual TB agar plates supplemented with 100 μg/mL rifampicin or without rifampicin. Plates were incubated at 37 °C for 18 hours before colony counting. The colonies grown on the TB agar plate containing rifampicin were collected for Sanger sequencing to determine the desired base conversion.

### Deep amplicon sequencing

Genomic DNA was extracted using Ezup Column Bacteria Genomic DNA Purification Kit (Sangon Biotech). The target genomic sites were amplified and sequenced on Illumina HiSeq. In brief, primers containing barcode sequences were used to amplify the target sites (PCR1). Each 10 μL PCR1 reaction was performed with 5 μL 2 × Phanta® Max Master Mix (Dye Plus), 3.5 μL H_2_O, 0.5 μL P5-Primer (10 μM), 0.5 μL P7-Primer (10 μM) and 0.5 μL genomic DNA with the following thermocycling conditions: 98 °C for 60 s; 40 cycles of [98 °C for 30 s, 60 °C for 60 s], followed by 72 °C for 60 s. The PCR products were purified, and digested with ExoI (NEB) before being purified using TIANquick Midi Purification Kit. The amplicon-seq libraries were prepared by using the VAHTS Universal DNA Library Prep Kit for Illumina (Vazyme) according to the manufacturer’s instructions. The PCR products were purified and subjected to Illumina HiSeq by HaploX Genomics Center.

### Quantification of deep amplicon sequencing data

The sequencing data were demultiplexed using barcodeSpliter and analyzed using CRISPResso2^37^. CRISPResso2 was run with “discard_indel_reads” for the calculation of substitution, insertion, and deletion efficiency. Substitution efficiency quantification requires the following parameters: ‘name’, ‘fastq_r1’, ‘fastq_r2’, ‘amplicon_seq’, ‘prime_editing_pegRNA_spacer_seq’, ‘prime_editing_pegRNA_extension_seq’ and ‘prime_editing_nicking_guide_seq’. For deletion or insertion edits, CRISPResso2 was run in the HDR mode. The substitution efficiency was calculated as the percentage of [number of unmodified reads (amplicon = prime-edited)]/[number of reads_aligned_all_amplicons]. The insertion or deletion efficiency was calculated as a percentage of [number of unmodified reads (amplicon = HDR)]/[number of reads_aligned_all_amplicons].

### RT-qPCR analysis

*E. coli* MG1655 containing the CRISPRi plasmid was 1:100 diluted into 100 mL TB supplemented with 100 μg/mL carbenicillin, 50 μg/mL chloramphenicol, 0.5 mM IPTG and 10 mM arabinose. When OD_600_ reached 0.5, the cell cultures were placed on ice for 10 min. 3 mL cell cultures were centrifuged, resuspended with Buffer RL and lysed using FastPrep-24 5 G (MP). The total RNA was further purified using TaKaRa MiniBEST Universal RNA Extraction Kit (TaKaRa) and verified by electrophoresis using 1% agarose gel. The cDNA was synthesized using *TransScript*® One-Step gDNA Removal and cDNA Synthesis SuperMix (TransGen Biotech Co.) according to the manufacturer’s instructions. Primers for qPCR were listed in Supplementary Table S3. qPCR was performed using ChamQ SYBR Color qPCR Master Mix in 10 μL reactions in LightCycler®96 (Roche) following the manufacturer’s instructions. The results were analyzed using LightCycler®96 application software. The expression of target genes was normalized to *idnT* and further analyzed using the 2^−ΔΔ*C*T^ method^38^.

### In vitro nuclease digestion assay

5’-FAM-labeled DNA duplex substrates were prepared by annealing three oligonucleotides (FAM-vitro1, vitro2 and vitro3 with a ratio of 1:1.1:1.1). Cleavage reactions were carried out in 1× cleavage buffer (50 mM Potassium Acetate, 20 mM Tris-acetate, 10 mM Magnesium Acetate, 100 µg/ml Recombinant Albumin, pH 7.9@25 °C). The final concentration of annealed DNA was 20 nM, and 1 U ExoI (NEB) or 1U ExoVII (HUZHENG) was used for the cleavage experiment. The reaction was prepared on ice and started by incubation at 37 °C, quenched by adding 2× formamide stop buffer (0.075% bromophenol blue, 0.075% xylene cyanol FF, 50 mM EDTA and 90% formamide) and then incubated at 95 °C for 10 min. The cleavage products were analyzed by a 20% TBE-Urea PAGE gel in 1× TBE running buffer at 50 °C and 120 V for 60 min. The TBE-Urea PAGE gel was visualized by the ChemiDoc MP System (Bio-Rad).

### Statistical analysis

GraphPad Prism (v.9.0.0) was used for statistical analysis. All numerical values are presented as mean ± SD.

### Reporting summary

Further information on research design is available in the Nature Portfolio Reporting Summary linked to this article.

### Supplementary information

Supplementary Information

Reporting Summary

Peer Review File

### Source data

Source Data

## Data Availability

The sequencing data generated in this study has been deposited in the NCBI under accession code PRJNA996576. Source data are provided as a Source Data file with this paper. Source data are provided in this paper.
