# Supplementary material for: BacPE: a versatile prime-editing platform in bacteria by inhibiting DNA exonucleases
Source: Nat Commun. 2024 Jan 27;15:825. doi: 10.1038/s41467-024-45114-4 (PMC10821919; doi:10.1038/s41467-024-45114-4)
Supplement: Supplementary file 1 — Supplementary Information [file 41467_2024_45114_MOESM1_ESM.pdf]

Table of contents

Supplementary Figures

Supplementary Figure 1. Schematic of the St1PE2 system for prime editing in *M. smegmatis* and *E. coli*.

Supplementary Figure 2. The effect of RT template length and PBS length on prime editing efficiency of St1PE2.

Supplementary Figure 3. Schematic of the SpPE system for prime editing in *E. coli*.

Supplementary Figure 4. PE3 nicks the non-target strand to increase prime editing efficiency.

Supplementary Figure 5. Mechanism of DNA mismatch repair in *E. coli*.

Supplementary Figure 6. The effect of MMR on prime editing efficiency in *E. coli*.

Supplementary Figure 7. The D516Y mutation introduced by St1PE2 system is confirmed by Sanger sequencing.

Supplementary Figure 8. Identification of key genetic determinants that affect insertion or deletion editing efficiency.

Supplementary Figure 9. Evaluation of the roles of 3′ →5′ DNA exonucleases in prime editing.

Supplementary Figure 10. CRISPRi-mediated repression of gene expression with different spacers.

Supplementary Tables

Supplementary Table 1. Strains used in this study.

Supplementary Table 2. Plasmids used in this study.

Supplementary Table 3. Primers used in this study.

Supplementary Notes

Supplementary Note 1. Overview of pegRNA cloning protocol.

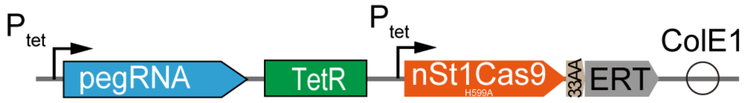

Supplementary Figure 1. Schematic of the St1PE2 system for prime editing in *M. smegmatis* and *E. coli*. The pegRNA and PE effecter are under the control of the inducible Ptet promoter.

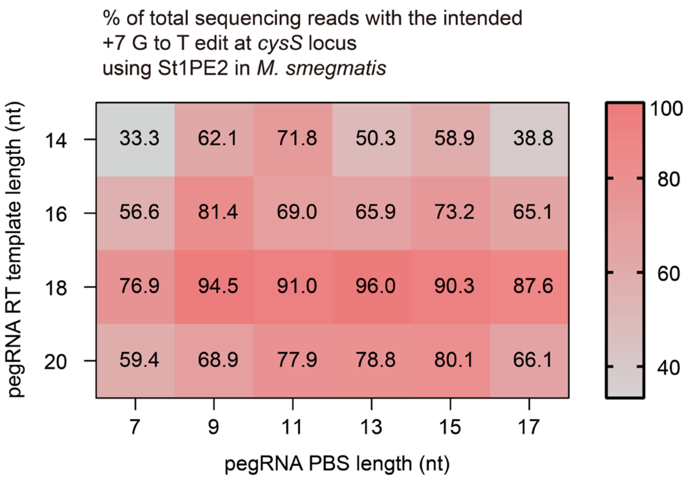

Supplementary Figure 2. The effect of RT template length and PBS length on prime editing efficiency of St1PE2. The heat maps show average editing efficiencies for given lengths of RT templates and PBS.

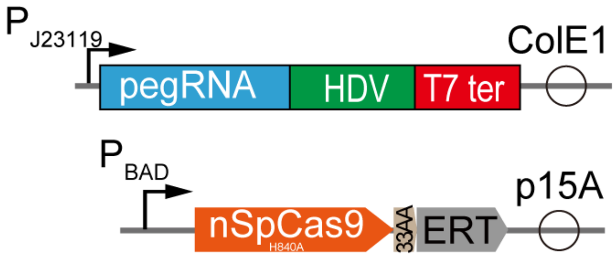

Supplementary Figure 3. Schematic of the SpPE system for prime editing in *E. coli*. The pegRNA and PE effector are encoded in two plasmids.

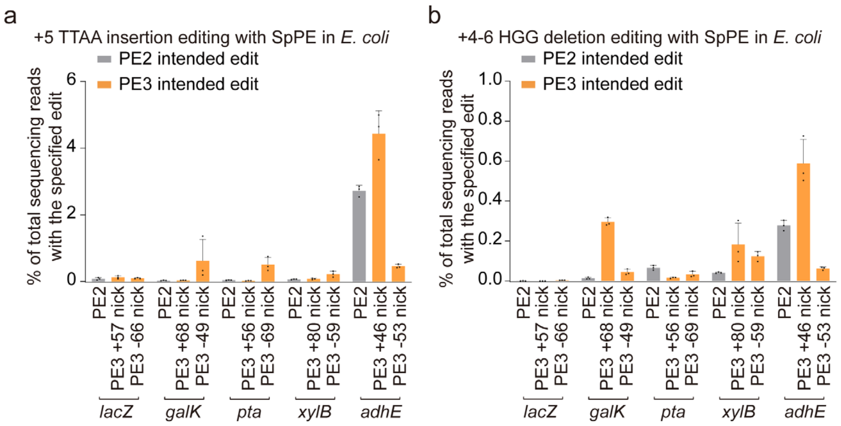

Supplementary Figure 4. PE3 nicks the non-target strand to increase prime editing efficiency. a, The effect of complementary strand nicking on insertion editing frequency. **b,** The effect of complementary strand nicking on deletion editing frequency.

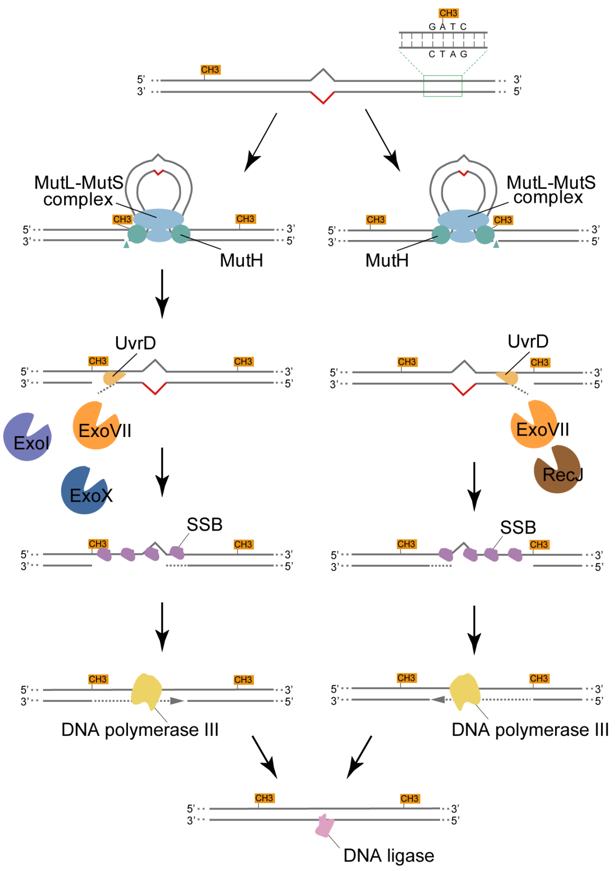

MutS recognizes mismatched base pair and recruits MutL. MutH is activated in a MutS-, MutL-, mismatch-dependent manner and incises the unmethylated strand at a hemi-methylated GATC site. MutH incision can occur either 5' or 3' to the mismatched base pair on the unmethylated strand.

The incised strand displaced by UvrD (DNA helicase II) is subjected to hydrolysis by single-strand specific exonucleases. When MutH incision occurs 5' to the mismatched base pair, ExoVII or recJ hydrolyzes ssDNA with 5' to 3' polarity. When MutH incision occurs 3' to the mismatched base pair, ExoI,ExoVII or ExoX hydrolyzes ssDNA with 3' to 5' polarity.

The single-stranded gap is stabilized by SSB.

DNA polymerase III resynthesizes the excised strand using the methylated strand as a template.

DNA ligase restores the helix integrity.

Supplementary Figure 5. Mechanism of DNA mismatch repair in *E. coli*. The newly synthesized strand is unmethylated.

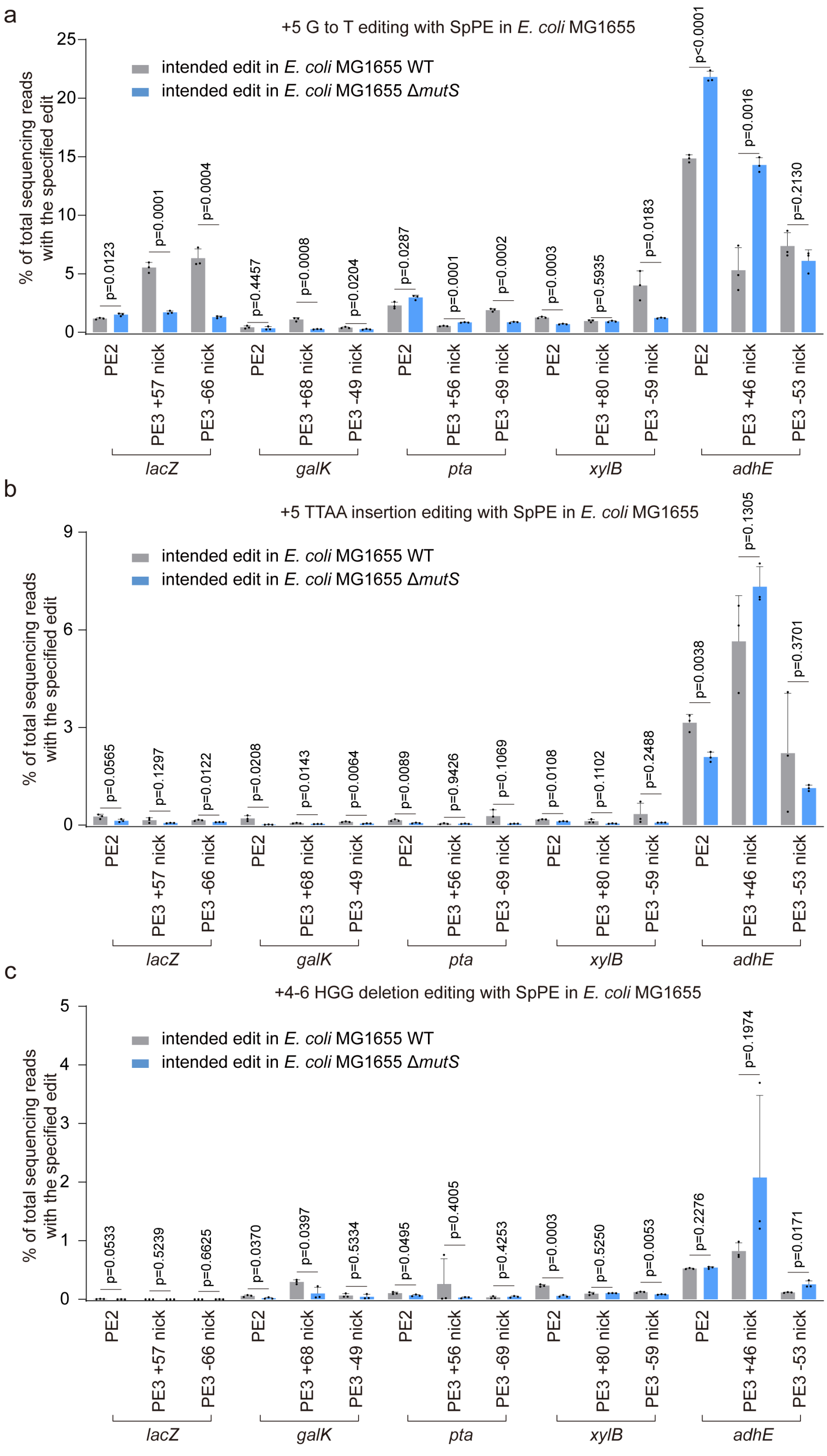

Supplementary Figure 6. The effect of MMR on prime editing efficiency in *E. coli*. **a,** +5 G to T editing efficiency in the WT strain and MMR-deficient strain. **b,** +5 TTAA insertion editing efficiency in the WT strain and MMR-deficient strain. **c,** +4-6 HGG deletion editing efficiency in the WT strain and MMR-deficient strain. H represents A, C or T. Data represent mean ± s.d. of n = 3 independent replicates.

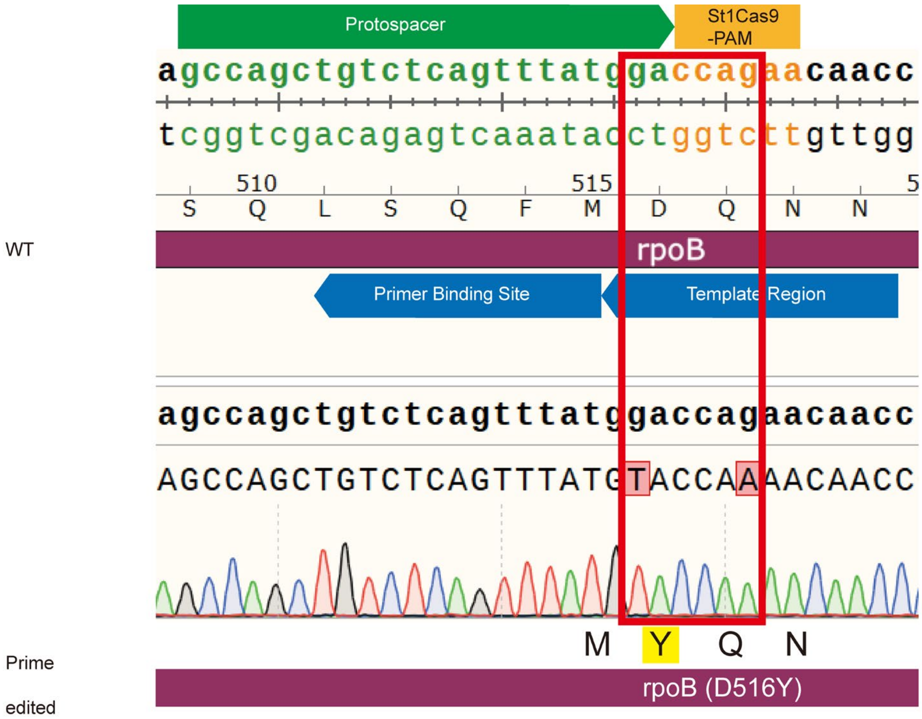

Supplementary Figure 7. The D516Y mutation introduced by St1PE2 system is confirmed by Sanger sequencing.

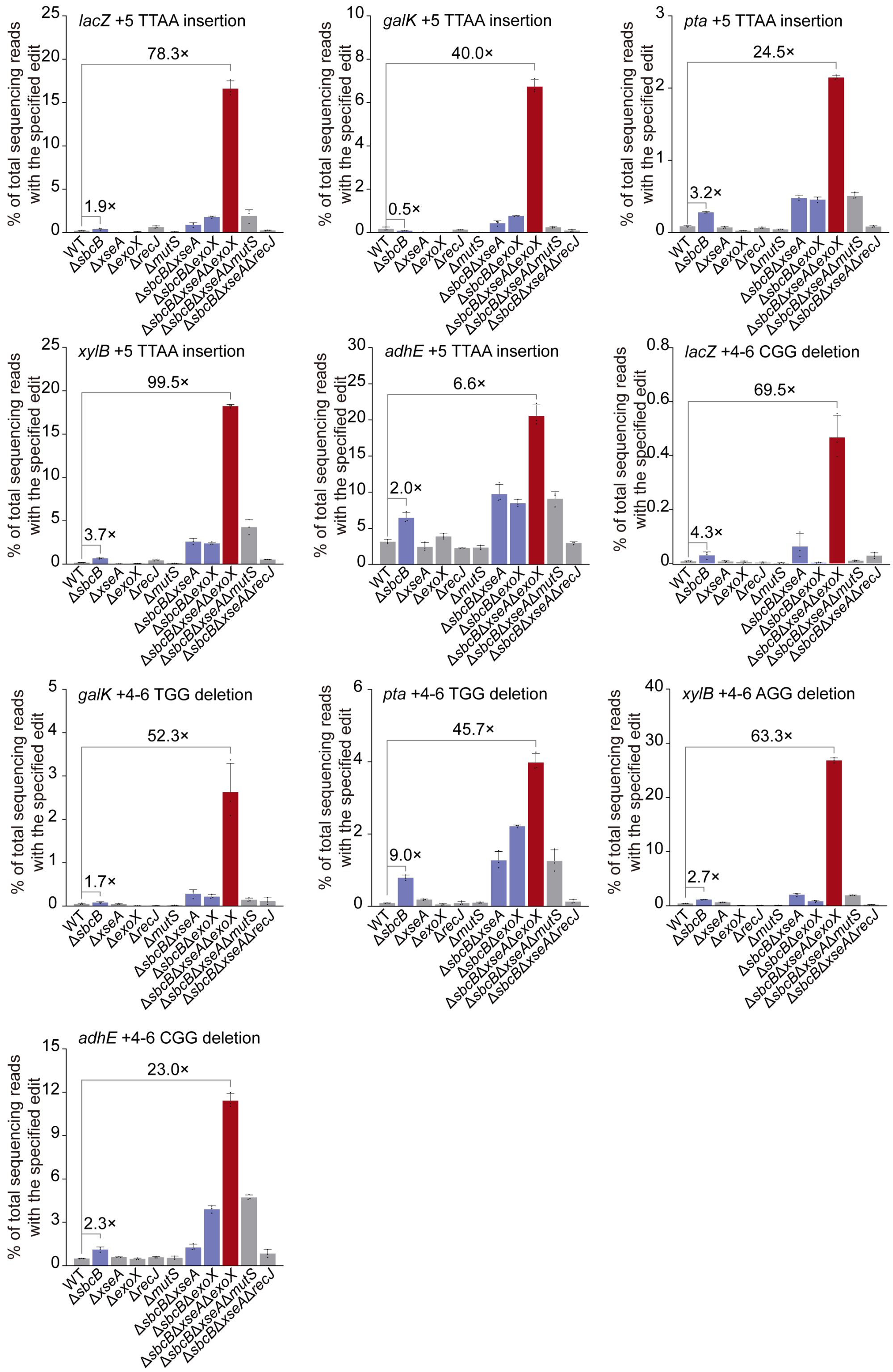

Supplementary Figure 8. Identification of key genetic determinants that affect insertion or deletion editing efficiency. Comparison of the +5 TTAA insertion or +4-6 HGG deletion efficiency in different *E. coli* mutant strains. H represents A, C or T. Δ*sbcB*, Δ*sbcB*Δ*xseA*, and Δ*sbcB*Δ*exoX* mutants are colored blue, and Δ*sbcB*Δ*xseA*Δ*exoX* mutants are colored red.

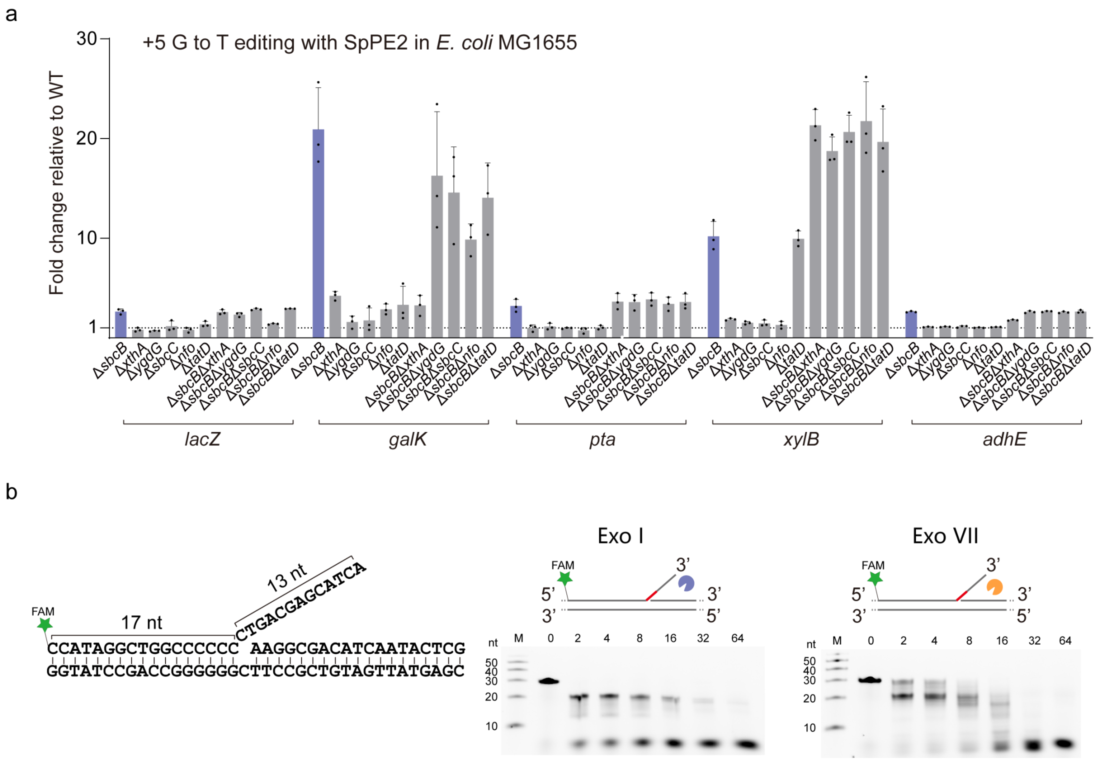

Supplementary Figure 9. Evaluation of the roles of 3′ →5′ DNA exonucleases in prime editing. a, The impact of 3’→5’ DNA exonucleases on prime editing efficiency. The dashed line represents a relative editing efficiency in the wild-type strain. **b,** Biochemical evidence for 3’-directed hydrolysis of PE intermediates. In vitro DNA cleavage results for FAM-labeled PE intermediates by exonucleases. PE intermediates were mimicked by the annealed oligonucleotides. The cleaved products were analyzed by TBE-Urea-PAGE.

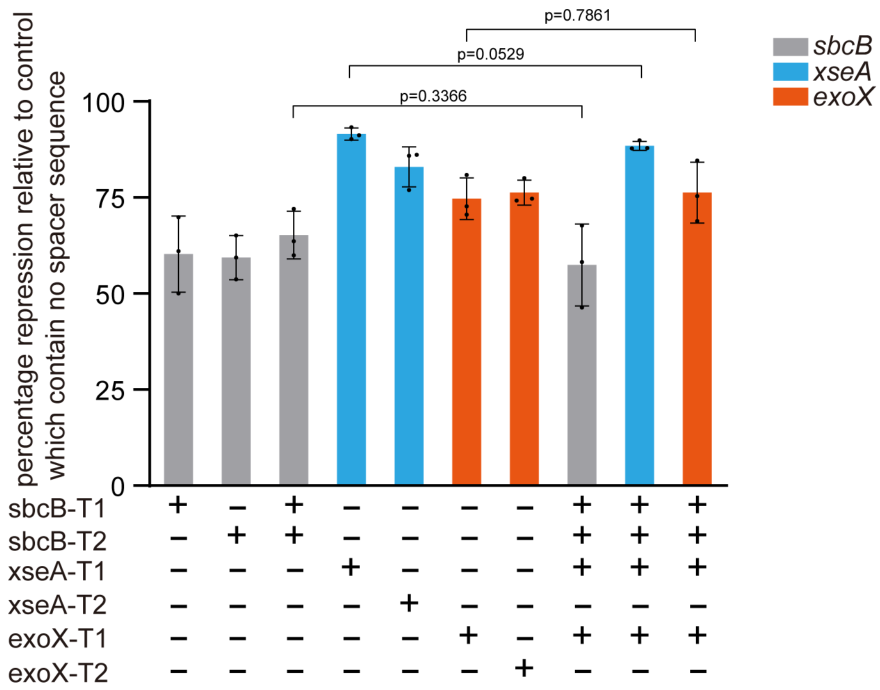

Supplementary Figure 10. CRISPRi-mediated repression of gene expression with different spacers. Gene-specific spacers were designed to target *scbB*, *xseA* or *exoX*. In the BacPE system, different crRNAs were assembled into a single plasmid to inhibit *sbcB*, *xseA* and *exoX* simultaneously. Student's *t*-test was performed. Data represent mean ± s.d. of n = 3 independent replicates.

Supplementary Table 1. Strains used in this study[1].

Organism	Strain	Description	Reference
E. coli	Top10	F-*mcrA*Δ(*mrr-hsdRMS-mcrBC*)φ80*lacZ*Δ*M15*Δ*lacX*74 *recA*1*ara*Δ139Δ(*ara-leu*)7697*galUgalKrpsL*(Str^R)*endA*1*nupG*	Lab stock
E. coli	BW25113	Δ*nhaR*	Baba *et al*., 2006
E. coli	BW25113	Δ*hns*	Baba *et al*., 2006
E. coli	BW25113	Δ*stpA*	Baba *et al*., 2006
E. coli	BW25113	Δ*hupB*	Baba *et al*., 2006
E. coli	BW25113	Δ*uvrY*	Baba *et al*., 2006
E. coli	BW25113	Δ*rnt*	Baba *et al*., 2006
E. coli	BW25113	Δ*aer*	Baba *et al*., 2006
E. coli	BW25113	Δ*helD*	Baba *et al*., 2006
E. coli	BW25113	Δ*yfcT*	Baba *et al*., 2006
E. coli	BW25113	Δ*mltB*	Baba *et al*., 2006
E. coli	BW25113	Δ*mltA*	Baba *et al*., 2006
E. coli	BW25113	Δ*yoaA*	Baba *et al*., 2006
E. coli	BW25113	Δ*radA*	Baba *et al*., 2006
E. coli	BW25113	Δ*recA*	Baba *et al*., 2006
E. coli	BW25113	Δ*yfcP*	Baba *et al*., 2006
E. coli	BW25113	Δ*yebG*	Baba *et al*., 2006
E. coli	BW25113	Δ*ydaL*	Baba *et al*., 2006
E. coli	BW25113	Δ*ynaK*	Baba *et al*., 2006
E. coli	BW25113	Δ*ybhP*	Baba *et al*., 2006
E. coli	BW25113	Δ*yajD*	Baba *et al*., 2006
E. coli	BW25113	Δ*ybcO*	Baba *et al*., 2006
E. coli	BW25113	Δ*yfcN*	Baba *et al*., 2006
E. coli	BW25113	Δ*yeeS*	Baba *et al*., 2006
E. coli	BW25113	Δ*yciV*	Baba *et al*., 2006
E. coli	BW25113	Δ*ybhM*	Baba *et al*., 2006
E. coli	BW25113	Δ*ykfG*	Baba *et al*., 2006
E. coli	BW25113	Δ*rarA*	Baba *et al*., 2006
E. coli	BW25113	Δ*ycfH*	Baba *et al*., 2006
E. coli	BW25113	Δ*ydaN*	Baba *et al*., 2006
E. coli	BW25113	Δ*ybhN*	Baba *et al*., 2006
E. coli	BW25113	Δ*ybhO*	Baba *et al*., 2006
E. coli	BW25113	Δ*ybhS*	Baba *et al*., 2006
E. coli	BW25113	Δ*ybhG*	Baba *et al*., 2006
E. coli	BW25113	Δ*yajC*	Baba *et al*., 2006
E. coli	BW25113	Δ*ybcD*	Baba *et al*., 2006
E. coli	BW25113	Δ*ybaZ*	Baba *et al*., 2006
E. coli	BW25113	Δ*uvrD*	Baba *et al*., 2006

Organism	Strain	Description	Reference
E. coli	BW25113	Δ*djlA*	Baba *et al*., 2006
E. coli	BW25113	Δ*dksA*	Baba *et al*., 2006
E. coli	BW25113	Δ*ruvB*	Baba *et al*., 2006
E. coli	BW25113	Δ*yfcO*	Baba *et al*., 2006
E. coli	BW25113	Δ*ogt*	Baba *et al*., 2006
E. coli	BW25113	Δ*ygjF*	Baba *et al*., 2006
E. coli	BW25113	Δ*yqjH*	Baba *et al*., 2006
E. coli	BW25113	Δ*yqjI*	Baba *et al*., 2006
E. coli	BW25113	Δ*ygjH*	Baba *et al*., 2006
E. coli	BW25113	Δ*yhbQ*	Baba *et al*., 2006
E. coli	BW25113	Δ*rmuC*	Baba *et al*., 2006
E. coli	BW25113	Δ*polA*	Baba *et al*., 2006
E. coli	BW25113	Δ*alkA*	Baba *et al*., 2006
E. coli	BW25113	Δ*recT*	Baba *et al*., 2006
E. coli	BW25113	Δ*sbcC*	Baba *et al*., 2006
E. coli	BW25113	Δ*mutL*	Baba *et al*., 2006
E. coli	BW25113	Δ*recR*	Baba *et al*., 2006
E. coli	BW25113	Δ*phr*	Baba *et al*., 2006
E. coli	BW25113	Δ*ruvA*	Baba *et al*., 2006
E. coli	BW25113	Δ*ruvC*	Baba *et al*., 2006
E. coli	BW25113	Δ*tus*	Baba *et al*., 2006
E. coli	BW25113	Δ*topB*	Baba *et al*., 2006
E. coli	BW25113	Δ*holE*	Baba *et al*., 2006
E. coli	BW25113	Δ*sbmC*	Baba *et al*., 2006
E. coli	BW25113	Δ*alkB*	Baba *et al*., 2006
E. coli	BW25113	Δ*slt*	Baba *et al*., 2006
E. coli	BW25113	Δ*dnaK*	Baba *et al*., 2006
E. coli	BW25113	Δ*dnaJ*	Baba *et al*., 2006
E. coli	BW25113	Δ*yfcV*	Baba *et al*., 2006
E. coli	BW25113	Δ*cbpA*	Baba *et al*., 2006
E. coli	BW25113	Δ*xseB*	Baba *et al*., 2006
E. coli	BW25113	Δ*rnhA*	Baba *et al*., 2006
E. coli	BW25113	Δ*uvrB*	Baba *et al*., 2006
E. coli	BW25113	Δ*rnhB*	Baba *et al*., 2006
E. coli	BW25113	Δ*sbcD*	Baba *et al*., 2006
E. coli	BW25113	Δ*nei*	Baba *et al*., 2006
E. coli	BW25113	Δ*xthA*	Baba *et al*., 2006
E. coli	BW25113	Δ*nfo*	Baba *et al*., 2006
E. coli	BW25113	Δ*recB*	Baba *et al*., 2006
E. coli	BW25113	Δ*recE*	Baba *et al*., 2006
E. coli	BW25113	Δ*dnaQ*	Baba *et al*., 2006
E. coli	BW25113	Δ*recJ*	Baba *et al*., 2006
E. coli	BW25113	Δ*dinB*	Baba *et al*., 2006

Organism	Strain	Description	Reference
E. coli	BW25113	Δ*xseA*	Baba *et al*., 2006
E. coli	BW25113	Δ*nth*	Baba *et al*., 2006
E. coli	BW25113	Δ*vsr*	Baba *et al*., 2006
E. coli	BW25113	Δ*endA*	Baba *et al*., 2006
E. coli	BW25113	Δ*ybhL*	Baba *et al*., 2006
E. coli	BW25113	Δ*tgt*	Baba *et al*., 2006
E. coli	BW25113	Δ*sbcB*	Baba *et al*., 2006
E. coli	BW25113	Δ*exoX*	Baba *et al*., 2006
E. coli	BW25113	Δ*uvrC*	Baba *et al*., 2006
E. coli	BW25113	Δ*ybcC*	Baba *et al*., 2006
E. coli	BW25113	Δ*rusA*	Baba *et al*., 2006
E. coli	BW25113	Δ*cspD*	Baba *et al*., 2006
E. coli	BW25113	Δ*dinD*	Baba *et al*., 2006
E. coli	BW25113	Δ*recG*	Baba *et al*., 2006
E. coli	BW25113	Δ*mltD*	Baba *et al*., 2006
E. coli	BW25113	Δ*ybhF*	Baba *et al*., 2006
E. coli	BW25113	Δ*exo*	Baba *et al*., 2006
E. coli	BW25113	Δ*ygjG*	Baba *et al*., 2006
E. coli	BW25113	Δ*nfi*	Baba *et al*., 2006
E. coli	BW25113	Δ*hda*	Baba *et al*., 2006
E. coli	BW25113	Δ*yicR*	Baba *et al*., 2006
E. coli	BW25113	Δ*yafD*	Baba *et al*., 2006
E. coli	BW25113	Δ*tatD*	Baba *et al*., 2006
E. coli	BW25113	Δ*ydcM*	Baba *et al*., 2006
E. coli	BW25113	Δ*ydaM*	Baba *et al*., 2006
E. coli	BW25113	Δ*mutH*	Baba *et al*., 2006
E. coli	BW25113	Δ*ung*	Baba *et al*., 2006
E. coli	BW25113	Δ*mltC*	Baba *et al*., 2006
E. coli	BW25113	Δ*ybhR*	Baba *et al*., 2006
E. coli	BW25113	Δ*ydjQ*	Baba *et al*., 2006
E. coli	BW25113	Δ*recO*	Baba *et al*., 2006
E. coli	BW25113	Δ*recN*	Baba *et al*., 2006
E. coli	BW25113	Δ*tag*	Baba *et al*., 2006
E. coli	BW25113	Δ*mutS*	Baba *et al*., 2006
E. coli	BW25113	Δ*pbl*	Baba *et al*., 2006
E. coli	BW25113	Δ*dam*	Baba *et al*., 2006
E. coli	BW25113	Δ*mutM*	Baba *et al*., 2006
E. coli	BW25113	Δ*ybhQ*	Baba *et al*., 2006
E. coli	BW25113	Δ*recF*	Baba *et al*., 2006
E. coli	BW25113	Δ*yfcS*	Baba *et al*., 2006
E. coli	BW25113	Δ*yfcU*	Baba *et al*., 2006
E. coli	BW25113	Δ*yfcQ*	Baba *et al*., 2006
E. coli	BW25113	Δ*dinG*	Baba *et al*., 2006

Organism	Strain	Description	Reference
E. coli	BW25113	Δ*recC*	Baba *et al*., 2006
E. coli	BW25113	Δ*recD*	Baba *et al*., 2006
E. coli	BW25113	Δ*polB*	Baba *et al*., 2006
E. coli	BW25113	Δ*emtA*	Baba *et al*., 2006
E. coli	BW25113	Δ*yeeY*	Baba *et al*., 2006
E. coli	BW25113	Δ*recQ*	Baba *et al*., 2006
E. coli	BW25113		Lab stock
M. smegmatis	mc^2155		Lab stock
E. coli	MG1655		Lab stock
E. coli	MG1655	Δ*sbcB*	This study
E. coli	MG1655	Δ*xseA*	This study
E. coli	MG1655	Δ*mutS*	This study
E. coli	MG1655	Δ*recJ*	This study
E. coli	MG1655	Δ*exoX*	This study
E. coli	MG1655	Δ*sbcB*Δ*xseA*	This study
E. coli	MG1655	Δ*sbcB*Δ*exoX*	This study
E. coli	MG1655	Δ*sbcB*Δ*xseA*Δ*exoX*	This study
E. coli	MG1655	Δ*sbcB*Δ*xseA*Δ*mutS*	This study
E. coli	MG1655	Δ*sbcB*Δ*xseA*Δ*recJ*	This study
E. coli	MG1655	Δ*xthA*	This study
E. coli	MG1655	Δ*ygdG*	This study
E. coli	MG1655	Δ*sbcC*	This study
E. coli	MG1655	Δ*nfo*	This study
E. coli	MG1655	*ΔtatD*	This study
E. coli	MG1655	Δ*sbcB*Δ*xthA*	This study
E. coli	MG1655	Δ*sbcB*Δ*ygdG*	This study
E. coli	MG1655	Δ*sbcB*Δ*sbcC*	This study
E. coli	MG1655	Δ*sbcB*Δ*nfo*	This study
E. coli	MG1655	Δ*sbcBΔtatD*	This study
K. pneumoniae	1.6366		Lab stock
K. pneumoniae	1.6366	Δ*sbcB*Δ*xseA*Δ*exoX*	This study
A. baumannii	ATCC17978		Lab stock
A. baumannii	ATCC17978	Δ*xseA*Δ*exoX*	This study

Supplementary Table 2. Plasmids used in this study[2, 3, 4, 5].

Plasmid ID	Description	Reference	Access link
MS_PE	Genome editing in *M. smegmatis*	This study	https://benchling.com/s/seq-0lAjOPTgqYsFA945VAd3?m=slm-82RyHHyq513P6TR28WDL
MS_PE_Cb	Genome editing in Keio mutants	This study	https://benchling.com/s/seq-x6t1tboKzgzcWCQq1en0?m=slm-IYtVOtvw6CnsIiZzbtJs
Sp_PE	Genome editing in *E. coli*, PE2 is under control of P_{BAD}	This study	https://benchling.com/s/seq-tSYVS1PpE5B1lZk5sJn3?m=slm-YZDhDKBPpbIfqkrktE57
pegRNA2	P_{J23119}-drived pegRNA expression	This study	https://benchling.com/s/seq-irwOdkHlaPmWbcuixwx6?m=slm-g2cY7ySHsFTOa3aNI4nc
pegRNA2_tevopreQ1	P_{J23119}-drived epegRNA expression	This study	https://benchling.com/s/seq-rOthM2vP1E2xZ32jf20U?m=slm-7BQKlJsvWWCEHYODYXUh
pegRNA2_mpknot	P_{J23119}-drived epegRNA expression	This study	https://benchling.com/s/seq-6nSPQeuoUAOJR1e4mU1L?m=slm-STwfOytMyZsuS8cbX8Ri
KP_PE	Genome editing in *K. pneumoniae*	This study	https://benchling.com/s/seq-UUSFYrnvTfrWvFZeumPk?m=slm-KccXdhXRRGEVv7gr8BfV
Ab_PE	Genome editing in *A. baumannii*	This study	https://benchling.com/s/seq-CDrVWPuUATPosQjI88m5?m=slm-hoDqFhhoXZbGtooxQ1l4
pBbS8c_ddCpf1_final	Inhibition of 3′-directed hydrolysis pathway	This study	https://benchling.com/s/seq-IV0cRNa1ngRHHJC0Dlto?m=slm-I4inF2LHwLFwRXe0ZqcN
pKD46-Cas9-RecA-Cure	*E. coli* genome editing vector	Bikard *et al.*, 2013	
pCRISPR	*E. coli* genome editing vector	Bikard *et al.*, 2013	
pBbS8c_ddCpf1	*E. coli* CRISPRi vector	Jervis *et al.*, 2021	
pBECAb	*A. baumannii* genome editing vector	Wang *et al.*, 2019	
pCasKP	*K. pneumoniae* genome editing vector	Wang *et al.*, 2018	
pSGKP	*K. pneumoniae* genome editing vector	Wang *et al.*, 2018	

Supplementary Table 3. Primers used in this study.

Name	Sequence (5’->3’)	Purpose
pegRNA spacer_F	TAGTNNNNNNNNNNNNNNNNNNNNNGTTTT	pegRNA assembly
pegRNA spacer_R	GAGATTTTGNNNNNNNNNNNNNNNNNNNN	pegRNA assembly
pegRNA 3′ Supension_F	GGTGCNNNNNNNNNNNNNNNNNNNNNNNNNNNNN	pegRNA assembly
pegRNA 3′ Supension_R	GCGCNNNNNNNNNNNNNNNNNNNNNNNNNNNNN	pegRNA assembly
pegRNA scaffold_F	AGAGCTAGAAATAGCAAGTTAAAATAAGGCTAGTCCGT TATCAACTTGAAAAAGTGGCACCGAGTC	pegRNA assembly
pegRNA scaffold_R	CGTGGCTGAGCCACGGTGAAAAAGTTCAACTATTGCCT GATCGGAATAAAATTGAACGATAAAGAT	pegRNA assembly
MSMEG_3634_UP-F	GGAgcggcacattcgacgaccgcGTCTTTGTACTCTGGTACCAGA AGCTACAAAGATAAGGCTTCATGCCGAAATCAAC	Fig. 1a
MSMEG_0200_UP-F	GGAgttgcccagtccgagcagacGTCTTTGTACTCTGGTACCAGA AGCTACAAAGATAAGGCTTCATGCCGAAATCAAC	Fig. 1a
MSMEG_3488_UP-F	GGAgcatcggactcgatcagcgtGTCTTTGTACTCTGGTACCAGA AGCTACAAAGATAAGGCTTCATGCCGAAATCAAC	Fig. 1a
MSMEG_3634_UP-R	ATGACAGGGTGTTGATTTCGGCATGAAGCCTTATCTTTG TAGCTTCTGGTACCAGAGTACAAAGACgcggtcgtcgaatgtgc cgc	Fig. 1a
MSMEG_0200_UP-R	ATGACAGGGTGTTGATTTCGGCATGAAGCCTTATCTTTG TAGCTTCTGGTACCAGAGTACAAAGACgtctgctcggactgggc aac	Fig. 1a
MSMEG_3488_UP-R	ATGACAGGGTGTTGATTTCGGCATGAAGCCTTATCTTTG TAGCTTCTGGTACCAGAGTACAAAGACacgctgatcgagtccga tgc	Fig. 1a
+7G-to-TMSMEG_3634_DN-F	ACCCTGTCATTTTATGGCAGGGTGTccacagttAtccgcggtcgtc gaatgtg	Fig. 1a
+6CTTinsMSMEG_3634_DN-F	ACCCTGTCATTTTATGGCAGGGTGTccacagttctAAGccgcggt cgtcgaatgtg	Fig. 1a
+6-8AGAdelMSMEG_3634_DN-F	ACCCTGTCATTTTATGGCAGGGTGTccacagtccgcggtcgtcgaa tgtg	Fig. 1a
+7G-to-TMSMEG_0200_DN-F	ACCCTGTCATTTTATGGCAGGGTGTccacacttAtcggtctgctcgg actggg	Fig. 1a
+6CTTinsMSMEG_0200_DN-F	ACCCTGTCATTTTATGGCAGGGTGTccacacttctAAGcggtctg ctcggactggg	Fig. 1a
+6-8AGAdelMSMEG_0200_DN-F	ACCCTGTCATTTTATGGCAGGGTGTccacactcggtctgctcggact ggg	Fig. 1a
+7G-to-TMSMEG_3488_DN-F	ACCCTGTCATTTTATGGCAGGGTGTcacagttAtcgacgctgatcg agtccg	Fig. 1a
+6CTTinsMSMEG_3488_DN-F	ACCCTGTCATTTTATGGCAGGGTGTcacagttctAAGcgacgctg atcgagtccg	Fig. 1a
+6-8AGAdelMSMEG_3488_DN-F	ACCCTGTCATTTTATGGCAGGGTGTcacagtcgacgctgatcgagt ccg	Fig. 1a
+7G-to-TMSMEG_3634_DN-R	AAAcacattcgacgaccgcggaTaactgtggACACCCTGCCATAAA	Fig. 1a
+6CTTinsMSMEG_3634_	AAAcacattcgacgaccgcggCTTagaactgtggACACCCTGCCATAA	Fig. 1a

DN-R	A	
+6-8AGAdelMSMEG_3634_DN-R	AAAcacattcgacgaccgcggactgtggACACCCTGCCATAAA	Fig. 1a
+7G-to-TMSMEG_0200_DN-R	AAAcccagtccgagcagaccgaTaagtgtggACACCCTGCCATAAA	Fig. 1a
+6CTTinsMSMEG_0200_DN-R	AAAcccagtccgagcagaccgCTTagaagtgtggACACCCTGCCATAAA	Fig. 1a
+6-8AGAdelMSMEG_0200_DN-R	AAAcccagtccgagcagaccgagtgtggACACCCTGCCATAAA	Fig. 1a
+7G-to-TMSMEG_3488_DN-R	AAAcggactcgatcagcgtcgaTaactgtgACACCCTGCCATAAA	Fig. 1a
+6CTTinsMSMEG_3488_DN-R	AAAcggactcgatcagcgtcgCTTagaactgtgACACCCTGCCATAAA	Fig. 1a
+6-8AGAdelMSMEG_3488_DN-R	AAAcggactcgatcagcgtcgactgtgACACCCTGCCATAAA	Fig. 1a
cysS_UP-F	GGAgatctggtgttcccgcaccaGTCTTTGTACTCTGGTACCAGAAGCTACAAAGATAAGGCTTCATGCCGAAATCAAC	Sup.2
cysS_UP-R	ATGACAGGGTGTTGATTTCGGCATGAAGCCTTATCTTTGTAGCTTCTGGTACCAGAGTACAAAGACtggtgcgggaacaccagatc	Sup.2
cysS+sapI_F	ACCCTGTCATTTTATGGCAGGGTGTggaagagcccgctcttcg	Sup.2
cysS+sapI_R	aaacgaagagcgggctcttccACACCCTGCCATAAA	Sup.2
cysS_opti_1_F	TGTtctcgttAtcgtggtgcggga	Sup.2
cysS_opti_2_F	TGTtctcgttAtcgtggtgcgggaac	Sup.2
cysS_opti_3_F	TGTtctcgttAtcgtggtgcgggaacac	Sup.2
cysS_opti_4_F	TGTtctcgttAtcgtggtgcgggaacacca	Sup.2
cysS_opti_5_F	TGTtctcgttAtcgtggtgcgggaacaccaga	Sup.2
cysS_opti_6_F	TGTtctcgttAtcgtggtgcgggaacaccagatc	Sup.2
cysS_opti_7_F	TGTgatctcgttAtcgtggtgcggga	Sup.2
cysS_opti_8_F	TGTgatctcgttAtcgtggtgcgggaac	Sup.2
cysS_opti_9_F	TGTgatctcgttAtcgtggtgcgggaacac	Sup.2
cysS_opti_10_F	TGTgatctcgttAtcgtggtgcgggaacacca	Sup.2
cysS_opti_11_F	TGTgatctcgttAtcgtggtgcgggaacaccaga	Sup.2
cysS_opti_12_F	TGTgatctcgttAtcgtggtgcgggaacaccagatc	Sup.2
cysS_opti_13_F	TGTgcgatctcgttAtcgtggtgcggga	Sup.2
cysS_opti_14_F	TGTgcgatctcgttAtcgtggtgcgggaac	Sup.2
cysS_opti_15_F	TGTgcgatctcgttAtcgtggtgcgggaacac	Sup.2
cysS_opti_16_F	TGTgcgatctcgttAtcgtggtgcgggaacacca	Sup.2
cysS_opti_17_F	TGTgcgatctcgttAtcgtggtgcgggaacaccaga	Sup.2
cysS_opti_18_F	TGTgcgatctcgttAtcgtggtgcgggaacaccagatc	Sup.2
cysS_opti_19_F	TGTgcgcgatctcgttAtcgtggtgcggga	Sup.2
cysS_opti_20_F	TGTgcgcgatctcgttAtcgtggtgcgggaac	Sup.2
cysS_opti_21_F	TGTgcgcgatctcgttAtcgtggtgcgggaacac	Sup.2
cysS_opti_22_F	TGTgcgcgatctcgttAtcgtggtgcgggaacacca	Sup.2
cysS_opti_23_F	TGTgcgcgatctcgttAtcgtggtgcgggaacaccaga	Sup.2
cysS_opti_24_F	TGTgcgcgatctcgttAtcgtggtgcgggaacaccagatc	Sup.2

cysS_opti_1_R	AAAtcccgcaccacgaTaacgaga	Sup.2
cysS_opti_2_R	AAAgttcccgcaccacgaTaacgaga	Sup.2
cysS_opti_3_R	AAAgtgttcccgcaccacgaTaacgaga	Sup.2
cysS_opti_4_R	AAAtggtgttcccgcaccacgaTaacgaga	Sup.2
cysS_opti_5_R	AAAtctggtgttcccgcaccacgaTaacgaga	Sup.2
cysS_opti_6_R	AAAgatctggtgttcccgcaccacgaTaacgaga	Sup.2
cysS_opti_7_R	AAAtcccgcaccacgaTaacgagatc	Sup.2
cysS_opti_8_R	AAAgttcccgcaccacgaTaacgagatc	Sup.2
cysS_opti_9_R	AAAgtgttcccgcaccacgaTaacgagatc	Sup.2
cysS_opti_10_R	AAAtggtgttcccgcaccacgaTaacgagatc	Sup.2
cysS_opti_11_R	AAAtctggtgttcccgcaccacgaTaacgagatc	Sup.2
cysS_opti_12_R	AAAgatctggtgttcccgcaccacgaTaacgagatc	Sup.2
cysS_opti_13_R	AAAtcccgcaccacgaTaacgagatcgc	Sup.2
cysS_opti_14_R	AAAgttcccgcaccacgaTaacgagatcgc	Sup.2
cysS_opti_15_R	AAAgtgttcccgcaccacgaTaacgagatcgc	Sup.2
cysS_opti_16_R	AAAtggtgttcccgcaccacgaTaacgagatcgc	Sup.2
cysS_opti_17_R	AAAtctggtgttcccgcaccacgaTaacgagatcgc	Sup.2
cysS_opti_18_R	AAAgatctggtgttcccgcaccacgaTaacgagatcgc	Sup.2
cysS_opti_19_R	AAAtcccgcaccacgaTaacgagatcgcgc	Sup.2
cysS_opti_20_R	AAAgttcccgcaccacgaTaacgagatcgcgc	Sup.2
cysS_opti_21_R	AAAgtgttcccgcaccacgaTaacgagatcgcgc	Sup.2
cysS_opti_22_R	AAAtggtgttcccgcaccacgaTaacgagatcgcgc	Sup.2
cysS_opti_23_R	AAAtctggtgttcccgcaccacgaTaacgagatcgcgc	Sup.2
cysS_opti_24_R	AAAgatctggtgttcccgcaccacgaTaacgagatcgcgc	Sup.2
hsdR_UP_F	GGAggtttcctgctgtttgccttGTCTTTGTACTCTGGTACCAGAAGCTACAAAGATAAGGCTTCATGCCGAAATCAAC	Fig. 1b
mrr_UP_F	GGAggcagaatactgtaggcaggGTCTTTGTACTCTGGTACCAGAAGCTACAAAGATAAGGCTTCATGCCGAAATCAAC	Fig. 1b
hsdM_T1_UP_F	GGAgatatgtacgaagggctgttGTCTTTGTACTCTGGTACCAGAAGCTACAAAGATAAGGCTTCATGCCGAAATCAAC	Fig. 1b
hsdM_T2_UP_F	GGAggtggcgaacccgaatcaggaGTCTTTGTACTCTGGTACCAGAAGCTACAAAGATAAGGCTTCATGCCGAAATCAAC	Fig. 1b
hsdS_T1_UP_F	GGAgatctacatggccagcacgtGTCTTTGTACTCTGGTACCAGAAGCTACAAAGATAAGGCTTCATGCCGAAATCAAC	Fig. 1b
hsdS_T2_UP_F	GGAgagccgcaacattctgtattGTCTTTGTACTCTGGTACCAGAAGCTACAAAGATAAGGCTTCATGCCGAAATCAAC	Fig. 1b
mcrC_UP_F	GGAgtcctttgttttgacctggaGTCTTTGTACTCTGGTACCAGAAGCTACAAAGATAAGGCTTCATGCCGAAATCAAC	Fig. 1b
mcrA_UP_F	GGAgctccaggaaaccagttgagGTCTTTGTACTCTGGTACCAGAAGCTACAAAGATAAGGCTTCATGCCGAAATCAAC	Fig. 1b
araC_UP_F	GGAgcgagtttcgtgccggttgtGTCTTTGTACTCTGGTACCAGAAGCTACAAAGATAAGGCTTCATGCCGAAATCAAC	Fig. 1b
hsdR_UP_R	ATGACAGGGTGTTGATTTCGGCATGAAGCCTTATCTTTGTAGCTTCTGGTACCAGAGTACAAAGACaaggcaaacagcaggaaacc	Fig. 1b
mrr_UP_R	ATGACAGGGTGTTGATTTCGGCATGAAGCCTTATCTTTGTAGCTTCTGGTACCAGAGTACAAAGACcctgcctacagtattctgcc	Fig. 1b
hsdM_T1_UP_R	ATGACAGGGTGTTGATTTCGGCATGAAGCCTTATCTTTG	Fig. 1b

	TAGCTTCTGGTACCAGAGTACAAAGACaacagcccttcgtacatatc	
hsdM_T2_UP_R	ATGACAGGGTGTTGATTTCGGCATGAAGCCTTATCTTTGTAGCTTCTGGTACCAGAGTACAAAGACtcctgattcgggttcgccacc	Fig. 1b
hsdS_T1_UP_R	ATGACAGGGTGTTGATTTCGGCATGAAGCCTTATCTTTGTAGCTTCTGGTACCAGAGTACAAAGACacgtgctggccatgtagatc	Fig. 1b
hsdS_T2_UP_R	ATGACAGGGTGTTGATTTCGGCATGAAGCCTTATCTTTGTAGCTTCTGGTACCAGAGTACAAAGACaatacagaatgttgcggctc	Fig. 1b
mcrC_UP_R	ATGACAGGGTGTTGATTTCGGCATGAAGCCTTATCTTTGTAGCTTCTGGTACCAGAGTACAAAGACtccaggtcaaaacaaaggac	Fig. 1b
mcrA_UP_R	ATGACAGGGTGTTGATTTCGGCATGAAGCCTTATCTTTGTAGCTTCTGGTACCAGAGTACAAAGACctcaactggtttcctggagc	Fig. 1b
araC_UP_R	ATGACAGGGTGTTGATTTCGGCATGAAGCCTTATCTTTGTAGCTTCTGGTACCAGAGTACAAAGACacaaccggcacgaaactcgc	Fig. 1b
hsdR_Mut_F	ACCCTGTCATTTTATGGCAGGGTGTgccattAtggaaggcaaacagcagga	Fig. 1b
mrr_Mut_F	ACCCTGTCATTTTATGGCAGGGTGTgtaattAtggcctgcctacagtattc	Fig. 1b
hsdM_T1_Mut_F	ACCCTGTCATTTTATGGCAGGGTGTgttcttAtgcaacagcccttcgtaca	Fig. 1b
hsdM_T2_Mut_F	ACCCTGTCATTTTATGGCAGGGTGTacagttAttatcctgattcgggttcg	Fig. 1b
hsdS_T1_Mut_F	ACCCTGTCATTTTATGGCAGGGTGTttagttAtgtacgtgctggccatgta	Fig. 1b
hsdS_T2_Mut_F	ACCCTGTCATTTTATGGCAGGGTGTtaacttAttaaatacagaatgttgcg	Fig. 1b
mcrC_Mut_F	ACCCTGTCATTTTATGGCAGGGTGTataattAtattccaggtcaaaacaaa	Fig. 1b
mcrA_Mut_F	ACCCTGTCATTTTATGGCAGGGTGTgtctttAtacctcaactggtttcctg	Fig. 1b
araC_Mut_F	ACCCTGTCATTTTATGGCAGGGTGTctttttAttcacaaccggcacgaaac	Fig. 1b
hsdR_Mut_R	AAAtcctgctgtttgccttccaTaatggcACACCCTGCCATAAA	Fig. 1b
mrr_Mut_R	AAAgaatactgtaggcaggccaTaattacACACCCTGCCATAAA	Fig. 1b
hsdM_T1_Mut_R	AAAtgtacgaagggctgttgcaTaagaacACACCCTGCCATAAA	Fig. 1b
hsdM_T2_Mut_R	AAAcgaacccgaatcaggataaTaactgtACACCCTGCCATAAA	Fig. 1b
hsdS_T1_Mut_R	AAAtacatggccagcacgtacaTaactaaACACCCTGCCATAAA	Fig. 1b
hsdS_T2_Mut_R	AAAcgcaacattctgtatttaaTaagttaACACCCTGCCATAAA	Fig. 1b
mcrC_Mut_R	AAAtttgttttgacctggaataTaattatACACCCTGCCATAAA	Fig. 1b
mcrA_Mut_R	AAAcaggaaaccagttgaggtaTaaagacACACCCTGCCATAAA	Fig. 1b
araC_Mut_R	AAAgtttcgtgccggttgtgaaTaaaaagACACCCTGCCATAAA	Fig. 1b
lacZ_F	TAGTaatcccgaatctctatcgtgGTTTT	Fig. 1cSup.3Sup.4Fig. 2c-gSup.7Sup.8Fig. 2iFig. 3h

galK_F	TAGTgacagccacacctttgggcaGTTTT	Fig. 1cSup.3Sup.4Fig. 2c-gSup.7Sup.8Fig. 2iFig. 3hFig. 4a
pta_F	TAGTacgcagcattccgcacatgcGTTTT	Fig. 1cSup.3Sup.4Fig. 2c-gSup.7Sup.8Fig. 2iFig. 3hFig. 4a
xylB_F	TAGTccccacgctttcgcaacttcGTTTT	Fig. 1cSup.3Sup.4Fig. 2c-gSup.7Sup.8Fig. 2iFig. 3hFig. 4a
adhE_F	TAGTctgcaggctgctatcgctgcGTTTT	Fig. 1cSup.3Sup.4Fig. 2c-gSup.7Sup.8Fig. 2iFig. 3hFig. 4a
lacZ_R	gctctaaaaccacgatagagattcgggatt	Fig. 1cSup.3Sup.4Fig. 2c-gSup.7Sup.8Fig. 2iFig. 3h
galK_R	gctctaaaactgcccaaaggtgtggctgtc	Fig. 1cSup.3Sup.4Fig. 2c-gSup.7Sup.8Fig. 2iFig. 3hFig. 4a
pta_R	gctctaaaacgcatgtgcggaatgctgcgt	Fig. 1cSup.3Sup.4Fig. 2c-gSup.7Sup.8Fig. 2iFig. 3hFig. 4a
xylB_R	gctctaaaacgaagttgcgaaagcgtgggg	Fig. 1cSup.3Sup.4Fig. 2c-gSup.7Sup.8Fig. 2iFig. 3hFig. 4a
adhE_R	gctctaaaacgcagcgatagcagcctgcag	Fig. 1cSup.3Sup.4Fig. 2c-gSup.7Sup.8Fig. 2iFig. 3hFig. 4a
pegRNA2lacZ+5G-to-T_F	ggtGctcaaccacAgcacgatagagattcgg	Fig. 1cSup.3Sup.4Fig. 2c-gSup.7Sup.8Fig. 2i
pegRNA2lacZ+5TTAA ins_F	ggtGctcaaccaccTTAAgcacgatagagattcgg	Fig. 1cSup.3Sup.4Fig. 2c-gSup.7
pegRNA2lacZ+4-6 CGG del_F	ggtGctcaaccacacgatagagattcgg	Fig. 1cSup.3Sup.4Fig. 2c-gSup.7
pegRNA2galK+5G-to-T_F	ggtGcgcagtttcAatgcccaaaggtgtggc	Fig. 1cSup.3Sup.4Fig. 2c-gSup.7Sup.8Fig.2i
pegRNA2galK+5TTAA ins_F	ggtGcgcagtttccTTAAatgcccaaaggtgtggc	Fig. 1cSup.3Sup.4Fig. 2c-gSup.7
pegRNA2galK+4-6 TGG del_F	ggtGcgcagttttgcccaaaggtgtggc	Fig. 1cSup.3Sup.4Fig. 2c-gSup.7
pegRNA2pta+5G-to-T_F	ggtGcaagtgctcAagcatgtgcggaatgct	Fig. 1cSup.3Sup.4Fig. 2c-gSup.7Sup.8Fig.2i
pegRNA2pta+5TTAA ins_F	ggtGcaagtgctccTTAAagcatgtgcggaatgct	Fig. 1cSup.3Sup.4Fig. 2c-gSup.7
pegRNA2pta+4-6 TGG del_F	ggtGcaagtgctgcatgtgcggaatgct	Fig. 1cSup.3Sup.4Fig. 2c-gSup.7
pegRNA2xylB+5G-to-T_F	ggtGctttgttacAtgaagttgcgaaagcgt	Fig. 1cSup.3Sup.4Fig. 2c-gSup.7Sup.8Fig.2i
pegRNA2xylB+5TTAA ins_F	ggtGctttgttaccTTAAtgaagttgcgaaagcgt	Fig. 1cSup.3Sup.4Fig. 2c-gSup.7
pegRNA2xylB+4-6 AGG	ggtGctttgttagaagttgcgaaagcgt	Fig. 1cSup.3Sup.4Fig. 2c-

del_F		gSup.7
pegRNA2adhE+5G-to-T_F	ggtGccggagcacAggcagcgatagcagcct	Fig. 1cSup.3Sup.4Fig. 2c-gSup.7Sup.8Fig.2i
pegRNA2adhE+5TTAA ins_F	ggtGccggagcaccTTAAggcagcgatagcagcct	Fig. 1cSup.3Sup.4Fig. 2c-gSup.7
pegRNA2adhE+4-6 CGG del_F	ggtGccggagcagcagcgatagcagcct	Fig. 1cSup.3Sup.4Fig. 2c-gSup.7
pegRNA2lacZ+5G-to-T_R	GGCCccgaatctctatcgtgcTgtggttga	Fig. 1cSup.3Sup.4Fig. 2c-gSup.7Sup.8Fig.2i
pegRNA2lacZ+5TTAA ins_R	GGCCccgaatctctatcgtgcTTAAggtggttga	Fig. 1cSup.3Sup.4Fig. 2c-gSup.7
pegRNA2lacZ+4-6 CGG del_R	GGCCccgaatctctatcgtgtggttga	Fig. 1cSup.3Sup.4Fig. 2c-gSup.7
pegRNA2galK+5G-to-T_R	GGCCgccacacctttgggcatTgaaactgc	Fig. 1cSup.3Sup.4Fig. 2c-gSup.7Sup.8Fig.2i
pegRNA2galK+5TTAA ins_R	GGCCgccacacctttgggcatTTAAggaaactgc	Fig. 1cSup.3Sup.4Fig. 2c-gSup.7
pegRNA2galK+4-6 TGG del_R	GGCCgccacacctttgggcaaaactgc	Fig. 1cSup.3Sup.4Fig. 2c-gSup.7
pegRNA2pta+5G-to-T_R	GGCCagcattccgcacatgctTgagcactt	Fig. 1cSup.3Sup.4Fig. 2c-gSup.7Sup.8Fig.2i
pegRNA2pta+5TTAA ins_R	GGCCagcattccgcacatgctTTAAggagcactt	Fig. 1cSup.3Sup.4Fig. 2c-gSup.7
pegRNA2pta+4-6 TGG del_R	GGCCagcattccgcacatgcagcactt	Fig. 1cSup.3Sup.4Fig. 2c-gSup.7
pegRNA2xylB+5G-to-T_R	GGCCacgctttcgcaacttcaTgtaacaaa	Fig. 1cSup.3Sup.4Fig. 2c-gSup.7Sup.8Fig.2i
pegRNA2xylB+5TTAA ins_R	GGCCacgctttcgcaacttcaTTAAggtaacaaa	Fig. 1cSup.3Sup.4Fig. 2c-gSup.7
pegRNA2xylB+4-6 AGG del_R	GGCCacgctttcgcaacttctaacaaa	Fig. 1cSup.3Sup.4Fig. 2c-gSup.7
pegRNA2adhE+5G-to-T_R	GGCCaggctgctatcgctgccTgtgctccg	Fig. 1cSup.3Sup.4Fig. 2c-gSup.7Sup.8Fig.2i
pegRNA2adhE+5TTAA ins_R	GGCCaggctgctatcgctgccTTAAggtgctccg	Fig. 1cSup.3Sup.4Fig. 2c-gSup.7
pegRNA2adhE+4-6 CGG del_R	GGCCaggctgctatcgctgctgctccg	Fig. 1cSup.3Sup.4Fig. 2c-gSup.7
lacZ(P+)+57_F	AGTCCTAGGTATAATACTAGTctcgcggaaaccgacatcgcgtttt	Fig. 1cSup.3Sup.4Fig. 3h
lacZ(P+)-66_F	AGTCCTAGGTATAATACTAGTcagacgtagtgtgacgcgatgtttt	Fig. 1cSup.3Sup.4
galK(P+)+68_F	AGTCCTAGGTATAATACTAGTgatcagctaatttccgcgctgtttt	Fig. 1cSup.3Sup.4Fig. 3h
galK(P+)-49_F	AGTCCTAGGTATAATACTAGTaacttcaaacgtaccctggtgtttt	Fig. 1cSup.3Sup.4
pta(P+)+56_F	AGTCCTAGGTATAATACTAGTcaagcggccaccagcacgtcgtttt	Fig. 1cSup.3Sup.4Fig. 3h
pta(P+)-69_F	AGTCCTAGGTATAATACTAGTgatgtcgccttcgttgatgagtttt	Fig. 1cSup.3Sup.4
xylB(P+)+80_F	AGTCCTAGGTATAATACTAGTctggagtgacgtcatgctgcgtttt	Fig. 1cSup.3Sup.4Fig. 3h
xylB(P+)-59_F	AGTCCTAGGTATAATACTAGTggcggtggcgacaatgcagcgtttt	Fig. 1cSup.3Sup.4
adhE(P+)+46_F	AGTCCTAGGTATAATACTAGTgcgttagacagttcaacagagtttt	Fig. 1cSup.3Sup.4Fig. 3h
adhE(P+)-53_F	AGTCCTAGGTATAATACTAGTttggtggcatcttttgcacggtttt	Fig. 1cSup.3Sup.4
lacZ(P+)+57_R	gctctaaaacgcgatgtcggtttccgcgagACTAGTATTATACCTAG	Fig. 1cSup.3Sup.4Fig. 3h
lacZ(P+)-66_R	gctctaaaacatcgcgtcacactacgtctgACTAGTATTATACCTAG	Fig. 1cSup.3Sup.4

galK(P+)+68_R	gctctaaaacagcgcggaaattagctgatcACTAGTATTATACCTAG	Fig. 1cSup.3Sup.4Fig. 3h
galK(P+)-49_R	gctctaaaacaccagggtacgtttgaagttACTAGTATTATACCTAG	Fig. 1cSup.3Sup.4
pta(P+)+56_R	gctctaaaacgacgtgctggtggccgcttgACTAGTATTATACCTAG	Fig. 1cSup.3Sup.4Fig. 3h
pta(P+)-69_R	gctctaaaactcatcaacgaaggcgacatcACTAGTATTATACCTAG	Fig. 1cSup.3Sup.4
xylB(P+)+80_R	gctctaaaacgcagcatgacgtcactccagACTAGTATTATACCTAG	Fig. 1cSup.3Sup.4Fig. 3h
xylB(P+)-59_R	gctctaaaacgctgcattgtcgccaccgccACTAGTATTATACCTAG	Fig. 1cSup.3Sup.4
adhE(P+)+46_R	gctctaaaactctgttgaactgtctaacgcACTAGTATTATACCTAG	Fig. 1cSup.3Sup.4Fig. 3h
adhE(P+)-53_R	gctctaaaaccgtgcaaaagatgccaccaaACTAGTATTATACCTAG	Fig. 1cSup.3Sup.4
rpoB_assay_UP_F	ggagccagctgtctcagtttatggaGTCTTTGTACTCTGGTACCAGAAGCTACAAAGATAAGGCTTCATGCCGAAATCAAC	Fig. 2bSup.6
rpoB_assay_UP_R	ATGACAGGGTGTTGATTTCGGCATGAAGCCTTATCTTTGTAGCTTCTGGTACCAGAGTACAAAGACtccataaactgagacagctggc	Fig. 2bSup.6
rpoB_assay_Mut_F	ACCCTGTCATTTTATGGCAGGGTGTgttgttTtggtAcataaactgagaca	Fig. 2bSup.6
rpoB_assay_Mut_R	aaAtgtctcagtttatgTaccaAaacaacACACCCTGCCATAAA	Fig. 2bSup.6
pegRNA2@tevopreQ1lacZ+5G-to-T_F	ggtGctcaaccacAgcacgatagagattcggAAACAATG	Fig.2i
pegRNA2@tevopreQ1galK+5G-to-T_F	ggtGcgcagtttcAatgcccaaaggtgtggcAAATTATC	Fig.2i
pegRNA2@tevopreQ1pta+5G-to-T_F	ggtGcaagtgctcAagcatgtgcggaatgctAATAAGAT	Fig.2i
pegRNA2@tevopreQ1xylB+5G-to-T_F	ggtGctttgttacAtgaagttgcgaaagcgtCATTAGAG	Fig.2i
pegRNA2@tevopreQ1adhE+5G-to-T_F	ggtGccggagcacAggcagcgatagcagcctAATTTAAT	Fig.2i
pegRNA2@mpknotlacZ+5G-to-T_F	ggtGctcaaccacAgcacgatagagattcggAAACAATT	Fig.2i
pegRNA2@mpknotgalK+5G-to-T_F	ggtGcgcagtttcAatgcccaaaggtgtggcAAATTATC	Fig.2i
pegRNA2@mpknotpta+5G-to-T_F	ggtGcaagtgctcAagcatgtgcggaatgctAATAAGAT	Fig.2i
pegRNA2@mpknotxylB+5G-to-T_F	ggtGctttgttacAtgaagttgcgaaagcgtCATTAGAG	Fig.2i
pegRNA2@mpknotadhE+5G-to-T_F	ggtGccggagcacAggcagcgatagcagcctAATTTAAT	Fig.2i
pegRNA2@tevopreQ1lacZ+5G-to-T_R	TCAACATTGTTTccgaatctctatcgtgcTgtggttga	Fig.2i
pegRNA2@tevopreQ1galK+5G-to-T_R	TCAAGATAATTTgccacacctttgggcatTgaaactgc	Fig.2i
pegRNA2@tevopreQ1pta+5G-to-T_R	TCAAATCTTATTagcattccgcacatgctTgagcactt	Fig.2i
pegRNA2@tevopreQ1xylB+5G-to-T_R	TCAACTCTAATGacgctttcgcaacttcaTgtaacaaa	Fig.2i
pegRNA2@tevopreQ1adhE+5G-to-T_R	TCAAATTAAATTaggctgctatcgctgccTgtgctccg	Fig.2i
pegRNA2@mpknotlacZ+5G-to-T_R	ACCCAATTGTTTccgaatctctatcgtgcTgtggttga	Fig.2i
pegRNA2@mpknotgalK+5	ACCCGATAATTTgccacacctttgggcatTgaaactgc	Fig.2i

G-to-T_R		
pegRNA2@mpknotpta+5G-to-T_R	ACCCATCTTATTagcattccgcacatgctTgagcactt	Fig.2i
pegRNA2@mpknotxylB+5G-to-T_R	ACCCCTCTAATGacgctttcgcaacttcaTgtaacaaa	Fig.2i
pegRNA2@mpknotadhE+5G-to-T_R	ACCCATTAAATTaggctgctatcgctgccTgtgctccg	Fig.2i
pCRISPR-sbcB_F	AAACtaattaccggtattaccccgcG	Fig. 2c-gSup.8Sup.9
pCRISPR-tatD_F	AAACgttaatgggctactcatcacG	Sup.8
pCRISPR-mutS_F	AAACccgagatcctgctgttttacG	Fig.1cFig. 2c-g
pCRISPR-xthA_F	AAACgtgacgacgaagaggcgcagG	Sup.8
pCRISPR-xseA_F	AAACgtgaccttccgcccacagcaG	Fig. 2c-gSup.9
pCRISPR-ygdG(xni)_F	AAACtcgtcgcattcatgccgttcG	Sup.8
pCRISPR-exoX_F	AAACgcctctgttgatgtcattgaG	Fig. 2c-g
pCRISPR-sbcC_F	AAACcctgctggacgccatttgtcG	Sup.8
pCRISPR-recJ_F	AAACgtgttaaaggtatgctgcccG	Fig. 2c-g
pCRISPR-nfo_F	AAACacatccggtcactgaagctcG	Sup.8
pCRISPR-sbcB_R	AAAACgcggggtaataccggtaatta	Fig. 2c-gSup.8Sup.9
pCRISPR-tatD_R	AAAACgtgatgagtagcccattaac	Sup.8
pCRISPR-mutS_R	AAAACgtaaaacagcaggatctcgg	Fig.1cFig. 2c-g
pCRISPR-xthA_R	AAAACctgcgcctcttcgtcgtcac	Sup.8
pCRISPR-xseA_R	AAAACtgctgtgggcggaaggtcac	Fig. 2c-gSup.9
pCRISPR-ygdG(xni)_R	AAAACgaacggcatgaatgcgacga	Sup.8
pCRISPR-exoX_R	AAAACtcaatgacatcaacagaggc	Fig. 2c-g
pCRISPR-sbcC_R	AAAACgacaaatggcgtccagcagg	Sup.8
pCRISPR-recJ_R	AAAACgggcagcataccttttaacac	Fig. 2c-g
pCRISPR-nfo_R	AAAACgagcttcagtgaccggatgt	Sup.8
FAM-vitro1	CCATAGGCTGGCCCCCC CTGACGAGCATCA	Sup.9b
vitro2	aaggcgacatcaatactcg	Sup.9b
vitro3	cgagtattgatgtcgccttcGGGGGgccAGCCTATGG	Sup.9b
ddCpf1@sbcB_T1_F	GTAGATacctaatgatgaatgacggtaagTTTTTTT	Sup.10
ddCpf1@sbcB_T2_F	GTAGATacgattacgaaacctttggcacgTTTTTTT	Sup.10
ddCpf1@xseA_T1_F	GTAGATgaatttgatctcgctcacatgttTTTTTTT	Sup.10
ddCpf1@xseA_T2_F	GTAGATatctcgctcacatgttaccttctTTTTTTT	Sup.10
ddCpf1@exoX_T1_F	GTAGATcactatgaaactggcccgtcgttTTTTTTT	Sup.10
ddCpf1@exoX_T2_F	GTAGATtggcctgggatcaagtacagcaaTTTTTTT	Sup.10
ddCpf1@sbcB_T1_R	cttcaaaaaaacttaccgtcattcatcattaggtAT	Sup.10
ddCpf1@sbcB_T2_R	cttcaaaaaaacgtgccaaaggtttcgtaatcgtAT	Sup.10
ddCpf1@xseA_T1_R	cttcaaaaaaaaacatgtgagcgagatcaaattcAT	Sup.10
ddCpf1@xseA_T2_R	cttcaaaaaaaagaaggtaacatgtgagcgagatAT	Sup.10
ddCpf1@exoX_T1_R	cttcaaaaaaaaacgacgggccagtttcatagtgAT	Sup.10
ddCpf1@exoX_T2_R	cttcaaaaaaattgctgtacttgatcccaggccaAT	Sup.10
qPCR_sbcB_F	gcgctgggatttactggatg	Sup.10
qPCR_sbcB_R	cagctttgccatcgcaatag	Sup.10
qPCR_xseA_F	agtgagccgtaatcagcaag	Sup.10
qPCR_xseA_R	ttccagcgcaaagctcattc	Sup.10
qPCR_exoX_F	ttgacggaaaaatcgtcaac	Sup.10
qPCR_exoX_R	ttcatagtgcaaatccactc	Sup.10

qPCR_idnT_F	GTGCGCCTCTTCTTTGAATTT	Sup.10
qPCR_idnT_R	TCGATGGTGCGTCCATTAC	Sup.10
EMxylB+1T-to-A_F	gtGcctttgttacctgaTgttgcgaaagcgtCTCCATTG	Fig. 3b-d
EMxylB+1T-to-G_F	gtGcctttgttacctgaCgttgcgaaagcgtCTCTTTCC	Fig. 3b-d
EMxylB+1T-to-C_F	gtGcctttgttacctgaGgttgcgaaagcgtTTAAGAAT	Fig. 3b-d
EMxylB+2T-to-A_F	gtGcctttgttacctgTagttgcgaaagcgtTCAAAGAT	Fig. 3b-d
EMxylB+2T-to-G_F	gtGcctttgttacctgCagttgcgaaagcgtTCAAAGAT	Fig. 3b-d
EMxylB+2T-to-C_F	gtGcctttgttacctgGagttgcgaaagcgtCCACATTT	Fig. 3b-d
EMxylB+3C-to-A_F	gtGcctttgttacctTaagttgcgaaagcgtTAAGACCT	Fig. 3b-d
EMxylB+3C-to-G_F	gtGcctttgttacctCaagttgcgaaagcgtTGATCTCA	Fig. 3b-d
EMxylB+3C-to-T_F	gtGcctttgttacctAaagttgcgaaagcgtTAAAGAAA	Fig. 3b-d
EMxylB+4A-to-G_F	gtGcctttgttaccCgaagttgcgaaagcgtCCATTATA	Fig. 3b-d
EMxylB+4A-to-C_F	gtGcctttgttaccGgaagttgcgaaagcgtCCATTATC	Fig. 3b-d
EMxylB+4A-to-T_F	gtGcctttgttaccAgaagttgcgaaagcgtATCAAAGA	Fig. 3b-d
EMxylB+5G-to-A_F	gtGcctttgttacTtgaagttgcgaaagcgtTCATCTAC	Fig. 3b-d
EMxylB+5G-to-C_F	gtGcctttgttacGtgaagttgcgaaagcgtTAAAGATC	Fig. 3b-d
EMxylB+5G-to-T_F	gtGcctttgttacAtgaagttgcgaaagcgtCATACCAA	Fig. 3b-d
EMxylB+6G-to-A_F	gtGcctttgttaTctgaagttgcgaaagcgtTCCATGAT	Fig. 3b-d
EMxylB+6G-to-C_F	gtGcctttgttaGctgaagttgcgaaagcgtTCATTCTA	Fig. 3b-d
EMxylB+6G-to-T_F	gtGcctttgttaActgaagttgcgaaagcgtTCAATCCA	Fig. 3b-d
EMxylB+7T-to-A_F	gtGcctttgttTcctgaagttgcgaaagcgtTCATACCG	Fig. 3b-d
EMxylB+7T-to-G_F	gtGcctttgttCcctgaagttgcgaaagcgtTCAATATA	Fig. 3b-d
EMxylB+7T-to-C_F	gtGcctttgttGcctgaagttgcgaaagcgtTCAAGATG	Fig. 3b-d
EMxylB+8A-to-G_F	gtGcctttgtCacctgaagttgcgaaagcgtTCAATAGA	Fig. 3b-d
EMxylB+8A-to-C_F	gtGcctttgtGacctgaagttgcgaaagcgtTAAATATT	Fig. 3b-d
EMxylB+8A-to-T_F	gtGcctttgtAacctgaagttgcgaaagcgtTTCCCAAG	Fig. 3b-d
EMxylB+1T ins_F	gtGcctttgttacctgaaAgttgcgaaagcgtTCATCTAA	Fig. 3b-d
EMxylB+1TA ins_F	gtGcctttgttacctgaaTAgttgcgaaagcgtTCATACCG	Fig. 3b-d
EMxylB+1TAA ins_F	gtGcctttgttacctgaaTTAgttgcgaaagcgtATTCGATA	Fig. 3b-d
EMxylB+2T ins_F	gtGcctttgttacctgaAagttgcgaaagcgtTCATCTAA	Fig. 3b-d
EMxylB+2TA ins_F	gtGcctttgttacctgaTAagttgcgaaagcgtTCCAAAGA	Fig. 3b-d
EMxylB+2TAA ins_F	gtGcctttgttacctgaTTAagttgcgaaagcgtTCAAAGAC	Fig. 3b-d
EMxylB+3T ins_F	gtGcctttgttacctgAaagttgcgaaagcgtTCATCTAA	Fig. 3b-d
EMxylB+3TA ins_F	gtGcctttgttacctgTAaagttgcgaaagcgtTTCAGAAT	Fig. 3b-d
EMxylB+3TAA ins_F	gtGcctttgttacctgTTAaagttgcgaaagcgtTATAGATC	Fig. 3b-d
EMxylB+4T ins_F	gtGcctttgttacctAgaagttgcgaaagcgtCTAGTCCG	Fig. 3b-d
EMxylB+4TA ins_F	gtGcctttgttacctTAgaagttgcgaaagcgtTCTACCCA	Fig. 3b-d
EMxylB+4TAA ins_F	gtGcctttgttacctTTAgaagttgcgaaagcgtTTAAATAT	Fig. 3b-d
EMxylB+5T ins_F	gtGcctttgttaccAtgaagttgcgaaagcgtTCAAATTG	Fig. 3b-d
EMxylB+5TA ins_F	gtGcctttgttaccTAtgaagttgcgaaagcgtTCAGTCCT	Fig. 3b-d
EMxylB+5TAA ins_F	gtGcctttgttaccTTAtgaagttgcgaaagcgtTCATCTTA	Fig. 3b-d
EMxylB+6T ins_F	gtGcctttgttacActgaagttgcgaaagcgtTCAATTAC	Fig. 3b-d
EMxylB+6TA ins_F	gtGcctttgttacTActgaagttgcgaaagcgtTTAAATTC	Fig. 3b-d
EMxylB+6TAA ins_F	gtGcctttgttacTTActgaagttgcgaaagcgtTAATAATA	Fig. 3b-d
EMxylB+7T ins_F	gtGcctttgttaAcctgaagttgcgaaagcgtTCAAATCC	Fig. 3b-d
EMxylB+7TA ins_F	gtGcctttgttaTAcctgaagttgcgaaagcgtTTCAATTC	Fig. 3b-d
EMxylB+7TAA ins_F	gtGcctttgttaTTAcctgaagttgcgaaagcgtTTCACCTC	Fig. 3b-d
EMxylB+8T ins_F	gtGcctttgttAacctgaagttgcgaaagcgtTCAAATCC	Fig. 3b-d

EMxylB+8TA ins_F	gtGcctttgttTAacctgaagttgcgaaagcgtTCAAATTG	Fig. 3b-d
EMxylB+8TAA ins_F	gtGcctttgttTTAacctgaagttgcgaaagcgtTCATGATA	Fig. 3b-d
EMxylB+1T del_F	gtGcctttgttacctgagttgcgaaagcgtTCAGCCTA	Fig. 3b-d
EMxylB+1-2TT del_F	gtGcctttgttacctggttgcgaaagcgtTACAAAGC	Fig. 3b-d
EMxylB+1-3TTC del_F	gtGcctttgttacctgttgcgaaagcgtATCAAAGC	Fig. 3b-d
EMxylB+2T del_F	gtGcctttgttacctgagttgcgaaagcgtTCAGCCTA	Fig. 3b-d
EMxylB+2-3TC del_F	gtGcctttgttacctagttgcgaaagcgtTATAAAGT	Fig. 3b-d
EMxylB+2-4TCA del_F	gtGcctttgttaccagttgcgaaagcgtTGAATCAC	Fig. 3b-d
EMxylB+3C del_F	gtGcctttgttacctaagttgcgaaagcgtTAGAAACC	Fig. 3b-d
EMxylB+3-4CA del_F	gtGcctttgttaccaagttgcgaaagcgtAACGAAGA	Fig. 3b-d
EMxylB+3-5CAG del_F	gtGcctttgttacaagttgcgaaagcgtTACAGAGC	Fig. 3b-d
EMxylB+4A del_F	gtGcctttgttaccgaagttgcgaaagcgtCCCTATTA	Fig. 3b-d
EMxylB+4-5AG del_F	gtGcctttgttacgaagttgcgaaagcgtCCAAACAG	Fig. 3b-d
EMxylB+4-6AGG del_F	gtGcctttgttagaagttgcgaaagcgtCTAGTCTC	Fig. 3b-d
EMxylB+5G del_F	gtGcctttgttactgaagttgcgaaagcgtTCAGCCTC	Fig. 3b-d
EMxylB+5-6GG del_F	gtGcctttgttatgaagttgcgaaagcgtTCAGTTAA	Fig. 3b-d
EMxylB+5-7GGT del_F	gtGcctttgtttgaagttgcgaaagcgtTCATCCTG	Fig. 3b-d
EMxylB+6G del_F	gtGcctttgttactgaagttgcgaaagcgtTCAGCCTC	Fig. 3b-d
EMxylB+6-7GT del_F	gtGcctttgttctgaagttgcgaaagcgtTCATACCT	Fig. 3b-d
EMxylB+6-8GTA del_F	gtGcctttgtctgaagttgcgaaagcgtTCATGTAA	Fig. 3b-d
EMxylB+7T del_F	gtGcctttgttcctgaagttgcgaaagcgtTCAATCAA	Fig. 3b-d
EMxylB+7-8TA del_F	gtGcctttgtcctgaagttgcgaaagcgtTCATTAAG	Fig. 3b-d
EMxylB+7-9TAA del_F	gtGcctttgcctgaagttgcgaaagcgtTCATTATC	Fig. 3b-d
EMxylB+8A del_F	gtGcctttgtacctgaagttgcgaaagcgtTCATGAAT	Fig. 3b-d
EMxylB+8-9AA del_F	gtGcctttgacctgaagttgcgaaagcgtTCAATGAA	Fig. 3b-d
EMxylB+8-10AAC del_F	gtGcctttacctgaagttgcgaaagcgtTCAATAAC	Fig. 3b-d
EMxylB+1T-to-A_R	TCAACAATGGAGacgctttcgcaacAtcaggtaacaaag	Fig. 3b-d
EMxylB+1T-to-G_R	TCAAGGAAAGAGacgctttcgcaacGtcaggtaacaaag	Fig. 3b-d
EMxylB+1T-to-C_R	TCAAATTCTTAAacgctttcgcaacCtcaggtaacaaag	Fig. 3b-d
EMxylB+2T-to-A_R	TCAAATCTTTGAacgctttcgcaactAcaggtaacaaag	Fig. 3b-d
EMxylB+2T-to-G_R	TCAAATCTTTGAacgctttcgcaactGcaggtaacaaag	Fig. 3b-d
EMxylB+2T-to-C_R	TCAAAAATGTGGacgctttcgcaactCcaggtaacaaag	Fig. 3b-d
EMxylB+3C-to-A_R	TCAAAGGTCTTAacgctttcgcaacttAaggtaacaaag	Fig. 3b-d
EMxylB+3C-to-G_R	TCAATGAGATCAacgctttcgcaacttGaggtaacaaag	Fig. 3b-d
EMxylB+3C-to-T_R	TCAATTTCTTTAacgctttcgcaacttTaggtaacaaag	Fig. 3b-d
EMxylB+4A-to-G_R	TCAATATAATGGacgctttcgcaacttcGggtaacaaag	Fig. 3b-d
EMxylB+4A-to-C_R	TCAAGATAATGGacgctttcgcaacttcCggtaacaaag	Fig. 3b-d
EMxylB+4A-to-T_R	TCAATCTTTGATacgctttcgcaacttcTggtaacaaag	Fig. 3b-d
EMxylB+5G-to-A_R	TCAAGTAGATGActttgttacTtgaagttgcgaaagcgt	Fig. 3b-d
EMxylB+5G-to-C_R	TCAAGATCTTTAacgctttcgcaacttcaCgtaacaaag	Fig. 3b-d
EMxylB+5G-to-T_R	TCAATTGGTATGacgctttcgcaacttcaTgtaacaaag	Fig. 3b-d
EMxylB+6G-to-A_R	TCAAATCATGGAacgctttcgcaacttcagAtaacaaag	Fig. 3b-d
EMxylB+6G-to-C_R	TCAATAGAATGAacgctttcgcaacttcagCtaacaaag	Fig. 3b-d
EMxylB+6G-to-T_R	TCAATGGATTGAacgctttcgcaacttcagTtaacaaag	Fig. 3b-d
EMxylB+7T-to-A_R	TCAACGGTATGAacgctttcgcaacttcaggAaacaaag	Fig. 3b-d
EMxylB+7T-to-G_R	TCAATATATTGAacgctttcgcaacttcaggGaacaaag	Fig. 3b-d
EMxylB+7T-to-C_R	TCAACATCTTGAacgctttcgcaacttcaggCaacaaag	Fig. 3b-d
EMxylB+8A-to-G_R	TCAATCTATTGAacgctttcgcaacttcaggtGacaaag	Fig. 3b-d

EMxylB+8A-to-C_R	TCAAAATATTTAacgctttcgcaacttcaggtCacaaag	Fig. 3b-d
EMxylB+8A-to-T_R	TCAACTTGGGAAacgctttcgcaacttcaggtTacaaag	Fig. 3b-d
EMxylB+1T ins_R	TCAATTAGATGAacgctttcgcaacTttcaggtaacaaag	Fig. 3b-d
EMxylB+1TA ins_R	TCAACGGTATGAacgctttcgcaacTAttcaggtaacaaag	Fig. 3b-d
EMxylB+1TAA ins_R	TCAATATCGAATacgctttcgcaacTAAttcaggtaacaaag	Fig. 3b-d
EMxylB+2T ins_R	TCAATTAGATGAacgctttcgcaactTtcaggtaacaaag	Fig. 3b-d
EMxylB+2TA ins_R	TCAATCTTTGGAacgctttcgcaactTAtcaggtaacaaag	Fig. 3b-d
EMxylB+2TAA ins_R	TCAAGTCTTTGAacgctttcgcaactTAAtcaggtaacaaag	Fig. 3b-d
EMxylB+3T ins_R	TCAATTAGATGAacgctttcgcaacttTcaggtaacaaag	Fig. 3b-d
EMxylB+3TA ins_R	TCAAATTCTGAAacgctttcgcaacttTAcaggtaacaaag	Fig. 3b-d
EMxylB+3TAA ins_R	TCAAGATCTATAacgctttcgcaacttTAAcaggtaacaaag	Fig. 3b-d
EMxylB+4T ins_R	TCAACGGACTAGacgctttcgcaacttcTaggtaacaaag	Fig. 3b-d
EMxylB+4TA ins_R	TCAATGGGTAGAacgctttcgcaacttcTAaggtaacaaag	Fig. 3b-d
EMxylB+4TAA ins_R	TCAAATATTTAAacgctttcgcaacttcTAAaggtaacaaag	Fig. 3b-d
EMxylB+5T ins_R	TCAACAATTTGAacgctttcgcaacttcaTggtaacaaag	Fig. 3b-d
EMxylB+5TA ins_R	TCAAAGGACTGAacgctttcgcaacttcaTAggtaacaaag	Fig. 3b-d
EMxylB+5TAA ins_R	TCAATAAGATGAacgctttcgcaacttcaTAAggtaacaaag	Fig. 3b-d
EMxylB+6T ins_R	TCAAGTAATTGAacgctttcgcaacttcagTgtaacaaag	Fig. 3b-d
EMxylB+6TA ins_R	TCAAGAATTTAAacgctttcgcaacttcagTAgtaacaaag	Fig. 3b-d
EMxylB+6TAA ins_R	TCAATATTATTAacgctttcgcaacttcagTAAgtaacaaag	Fig. 3b-d
EMxylB+7T ins_R	TCAAGGATTTGAacgctttcgcaacttcaggTtaacaaag	Fig. 3b-d
EMxylB+7TA ins_R	TCAAGAATTGAAacgctttcgcaacttcaggTAtaacaaag	Fig. 3b-d
EMxylB+7TAA ins_R	TCAAGAGGTGAAacgctttcgcaacttcaggTAAtaacaaag	Fig. 3b-d
EMxylB+8T ins_R	TCAAGGATTTGAacgctttcgcaacttcaggtTaacaaag	Fig. 3b-d
EMxylB+8TA ins_R	TCAACAATTTGAacgctttcgcaacttcaggtTAaacaaag	Fig. 3b-d
EMxylB+8TAA ins_R	TCAATATCATGAacgctttcgcaacttcaggtTAAaacaaag	Fig. 3b-d
EMxylB+1T del_R	TCAATAGGCTGAacgctttcgcaactcaggtaacaaag	Fig. 3b-d
EMxylB+1-2TT del_R	TCAAGCTTTGTAacgctttcgcaaccaggtaacaaag	Fig. 3b-d
EMxylB+1-3TTC del_R	TCAAGCTTTGATacgctttcgcaacaggtaacaaag	Fig. 3b-d
EMxylB+2T del_R	TCAATAGGCTGAacgctttcgcaactcaggtaacaaag	Fig. 3b-d
EMxylB+2-3TC del_R	TCAAACTTTATAacgctttcgcaactaggtaacaaag	Fig. 3b-d
EMxylB+2-4TCA del_R	TCAAGTGATTCAacgctttcgcaactggtaacaaag	Fig. 3b-d
EMxylB+3C del_R	TCAAGGTTTCTAacgctttcgcaacttaggtaacaaag	Fig. 3b-d
EMxylB+3-4CA del_R	TCAATCTTCGTTacgctttcgcaacttggtaacaaag	Fig. 3b-d
EMxylB+3-5CAG del_R	TCAAGCTCTGTAacgctttcgcaacttgtaacaaag	Fig. 3b-d
EMxylB+4A del_R	TCAATAATAGGGacgctttcgcaacttcggtaacaaag	Fig. 3b-d
EMxylB+4-5AG del_R	TCAACTGTTTGGacgctttcgcaacttcgtaacaaag	Fig. 3b-d
EMxylB+4-6AGG del_R	TCAAGAGACTAGacgctttcgcaacttctaacaaag	Fig. 3b-d
EMxylB+5G del_R	TCAAGAGGCTGAacgctttcgcaacttcagtaacaaag	Fig. 3b-d
EMxylB+5-6GG del_R	TCAATTAACTGAacgctttcgcaacttcataacaaag	Fig. 3b-d
EMxylB+5-7GGT del_R	TCAACAGGATGAacgctttcgcaacttcaaacaaag	Fig. 3b-d
EMxylB+6G del_R	TCAAGAGGCTGAacgctttcgcaacttcagtaacaaag	Fig. 3b-d
EMxylB+6-7GT del_R	TCAAAGGTATGAacgctttcgcaacttcagaacaaag	Fig. 3b-d
EMxylB+6-8GTA del_R	TCAATTACATGAacgctttcgcaacttcagacaaag	Fig. 3b-d
EMxylB+7T del_R	TCAATTGATTGAacgctttcgcaacttcaggaacaaag	Fig. 3b-d
EMxylB+7-8TA del_R	TCAACTTAATGAacgctttcgcaacttcaggacaaag	Fig. 3b-d
EMxylB+7-9TAA del_R	TCAAGATAATGAacgctttcgcaacttcaggcaaag	Fig. 3b-d
EMxylB+8A del_R	TCAAATTCATGAacgctttcgcaacttcaggtacaaag	Fig. 3b-d

EMxylB+8-9AA del_R	TCAATTCATTGAacgctttcgcaacttcaggtcaaag	Fig. 3b-d
EMxylB+8-10AAC del_R	TCAAGTTATTGAacgctttcgcaacttcaggtaaag	Fig. 3b-d
EMlacZ+6GT_F	TAGTaatcccgaatctctatcgtgGTTTT	Fig. 3e-g
EMrpoB+6GT_F	TAGTcagccagctgtctcagtttaGTTTT	Fig. 3e-g
EMxylB+5GT_F	TAGTccccacgctttcgcaacttcGTTTT	Fig. 3e-g
EMlacZ+6GT_R	gctctaaaaccacgatagagattcgggatt	Fig. 3e-g
EMrpoB+6GT_R	gctctaaaactaaactgagacagctggctg	Fig. 3e-g
EMxylB+5GT_R	gctctaaaacgaagttgcgaaagcgtgggg	Fig. 3e-g
EMlacZ+6GTRTT=8_F	ggtGccaAcgcacgatagagattcggAAACAAGC	Fig. 3e-g
EMlacZ+6GTRTT=9_F	ggtGcccaAcgcacgatagagattcggAACATAAT	Fig. 3e-g
EMlacZ+6GTRTT=10_F	ggtGcaccaAcgcacgatagagattcggAACACATA	Fig. 3e-g
EMlacZ+6GTRTT=11_F	ggtGcaaccaAcgcacgatagagattcggAACCATAA	Fig. 3e-g
EMlacZ+6GTRTT=12_F	ggtGccaaccaAcgcacgatagagattcggAAACAATA	Fig. 3e-g
EMlacZ+6GTRTT=13_F	ggtGctcaaccaAcgcacgatagagattcggAAACAATG	Fig. 3e-g
EMlacZ+6GTRTT=14_F	ggtGcttcaaccaAcgcacgatagagattcggAAACAAGA	Fig. 3e-g
EMlacZ+6GTRTT=15_F	ggtGcgttcaaccaAcgcacgatagagattcggACCAACTA	Fig. 3e-g
EMlacZ+6GTRTT=16_F	ggtGcagttcaaccaAcgcacgatagagattcggAAACATAC	Fig. 3e-g
EMlacZ+6GTRTT=17_F	ggtGccagttcaaccaAcgcacgatagagattcggAAACAAAT	Fig. 3e-g
EMlacZ+6GTRTT=18_F	ggtGcgcagttcaaccaAcgcacgatagagattcggAAACAAAT	Fig. 3e-g
EMlacZ+6GTRTT=19_F	ggtGctgcagttcaaccaAcgcacgatagagattcggTACAATAA	Fig. 3e-g
EMlacZ+6GTRTT=20_F	ggtGcgtgcagttcaaccaAcgcacgatagagattcggACAGCAAG	Fig. 3e-g
EMlacZ+6GTRTT=21_F	ggtGctgtgcagttcaaccaAcgcacgatagagattcggAACAAATG	Fig. 3e-g
EMlacZ+6GTRTT=22_F	ggtGcgtgtgcagttcaaccaAcgcacgatagagattcggACATAATG	Fig. 3e-g
EMlacZ+6GTRTT=23_F	ggtGcggtgtgcagttcaaccaAcgcacgatagagattcggCAAACAAT	Fig. 3e-g
EMlacZ+6GTRTT=24_F	ggtGccggtgtgcagttcaaccaAcgcacgatagagattcggACAAAGTG	Fig. 3e-g
EMlacZ+6GTRTT=25_F	ggtGcgcggtgtgcagttcaaccaAcgcacgatagagattcggACATAAGT	Fig. 3e-g
EMlacZ+6GTRTT=26_F	ggtGcggcggtgtgcagttcaaccaAcgcacgatagagattcggACATATGC	Fig. 3e-g
EMlacZ+6GTRTT=27_F	ggtGccggcggtgtgcagttcaaccaAcgcacgatagagattcggAACAATAA	Fig. 3e-g
EMlacZ+6GTRTT=28_F	ggtGctcggcggtgtgcagttcaaccaAcgcacgatagagattcggAACATATA	Fig. 3e-g
EMlacZ+6GTRTT=29_F	ggtGcgtcggcggtgtgcagttcaaccaAcgcacgatagagattcggCCCAGTA C	Fig. 3e-g
EMlacZ+6GTRTT=30_F	ggtGcccgaatctctatcgtgcgTtggttgaactgcacaccgccgacgCAACAAC A	Fig. 3e-g
EMlacZ+6GTRTT=31_F	ggtGcccgtcggcggtgtgcagttcaaccaAcgcacgatagagattcggTTAAAC AC	Fig. 3e-g
EMrpoB+6GTRTT=8_F	ggtGcggtAcataaactgagacagctATAATTAC	Fig. 3e-g
EMrpoB+6GTRTT=9_F	ggtGctggtAcataaactgagacagctATATAATG	Fig. 3e-g
EMrpoB+6GTRTT=10_F	ggtGcctggtAcataaactgagacagctAAATTAAG	Fig. 3e-g
EMrpoB+6GTRTT=11_F	ggtGctctggtAcataaactgagacagctACAAATTA	Fig. 3e-g
EMrpoB+6GTRTT=12_F	ggtGcttctggtAcataaactgagacagctACATAAAT	Fig. 3e-g
EMrpoB+6GTRTT=13_F	ggtGcgttctggtAcataaactgagacagctATAATTAT	Fig. 3e-g
EMrpoB+6GTRTT=14_F	ggtGctgttctggtAcataaactgagacagctAATCCCTT	Fig. 3e-g
EMrpoB+6GTRTT=15_F	ggtGcttgttctggtAcataaactgagacagctTTAAATCG	Fig. 3e-g
EMrpoB+6GTRTT=16_F	ggtGcgttgttctggtAcataaactgagacagctAATTCCAC	Fig. 3e-g
EMrpoB+6GTRTT=17_F	ggtGcggttgttctggtAcataaactgagacagctTAAATCCG	Fig. 3e-g
EMrpoB+6GTRTT=18_F	ggtGcgggttgttctggtAcataaactgagacagctATATAGAC	Fig. 3e-g
EMrpoB+6GTRTT=19_F	ggtGccgggttgttctggtAcataaactgagacagctATATAATT	Fig. 3e-g
EMrpoB+6GTRTT=20_F	ggtGcgcgggttgttctggtAcataaactgagacagctATTAACAT	Fig. 3e-g

EMrpoB+6GTRTT=21_F	ggtGcagcgggttgttctggtAcataaactgagacagctATAGATCG	Fig. 3e-g
EMrpoB+6GTRTT=22_F	ggtGccagcgggttgttctggtAcataaactgagacagctATAACAAA	Fig. 3e-g
EMrpoB+6GTRTT=23_F	ggtGcacagcgggttgttctggtAcataaactgagacagctATAATATA	Fig. 3e-g
EMrpoB+6GTRTT=24_F	ggtGcgacagcgggttgttctggtAcataaactgagacagctAATTAGAC	Fig. 3e-g
EMrpoB+6GTRTT=25_F	ggtGcagacagcgggttgttctggtAcataaactgagacagctAATAATAT	Fig. 3e-g
EMrpoB+6GTRTT=26_F	ggtGccagacagcgggttgttctggtAcataaactgagacagctAATAACAT	Fig. 3e-g
EMrpoB+6GTRTT=27_F	ggtGctcagacagcgggttgttctggtAcataaactgagacagctATATAGAT	Fig. 3e-g
EMrpoB+6GTRTT=28_F	ggtGcctcagacagcgggttgttctggtAcataaactgagacagctAATTATAC	Fig. 3e-g
EMrpoB+6GTRTT=29_F	ggtGctctcagacagcgggttgttctggtAcataaactgagacagctAAATTATA	Fig. 3e-g
EMrpoB+6GTRTT=30_F	ggtGcatctcagacagcgggttgttctggtAcataaactgagacagctATAAATAT	Fig. 3e-g
EMrpoB+6GTRTT=31_F	ggtGcaatctcagacagcgggttgttctggtAcataaactgagacagctATAAATAC	Fig. 3e-g
EMxylB+6GTRTT=8_F	ggtGctacAtgaagttgcgaaagcgtTCAATTAA	Fig. 3e-g
EMxylB+6GTRTT=9_F	ggtGcttacAtgaagttgcgaaagcgtTCAAATAG	Fig. 3e-g
EMxylB+6GTRTT=10_F	ggtGcgttacAtgaagttgcgaaagcgtCATATATA	Fig. 3e-g
EMxylB+6GTRTT=11_F	ggtGctgttacAtgaagttgcgaaagcgtTCAAGATC	Fig. 3e-g
EMxylB+6GTRTT=12_F	ggtGcttgttacAtgaagttgcgaaagcgtTCATACTC	Fig. 3e-g
EMxylB+6GTRTT=13_F	ggtGctttgttacAtgaagttgcgaaagcgtCATTAGAG	Fig. 3e-g
EMxylB+6GTRTT=14_F	ggtGcctttgttacAtgaagttgcgaaagcgtCATACCAA	Fig. 3e-g
EMxylB+6GTRTT=15_F	ggtGcgctttgttacAtgaagttgcgaaagcgtCCTAGAGT	Fig. 3e-g
EMxylB+6GTRTT=16_F	ggtGctgctttgttacAtgaagttgcgaaagcgtTTATCAAC	Fig. 3e-g
EMxylB+6GTRTT=17_F	ggtGcgtgctttgttacAtgaagttgcgaaagcgtTCCCGAGT	Fig. 3e-g
EMxylB+6GTRTT=18_F	ggtGcggtgctttgttacAtgaagttgcgaaagcgtATAATAGA	Fig. 3e-g
EMxylB+6GTRTT=19_F	ggtGctggtgctttgttacAtgaagttgcgaaagcgtAAATATCC	Fig. 3e-g
EMxylB+6GTRTT=20_F	ggtGcctggtgctttgttacAtgaagttgcgaaagcgtAAATAAAT	Fig. 3e-g
EMxylB+6GTRTT=21_F	ggtGcactggtgctttgttacAtgaagttgcgaaagcgtATACATCC	Fig. 3e-g
EMxylB+6GTRTT=22_F	ggtGctactggtgctttgttacAtgaagttgcgaaagcgtAAATATCC	Fig. 3e-g
EMxylB+6GTRTT=23_F	ggtGcttactggtgctttgttacAtgaagttgcgaaagcgtTTACACAC	Fig. 3e-g
EMxylB+6GTRTT=24_F	ggtGcattactggtgctttgttacAtgaagttgcgaaagcgtAAATATTG	Fig. 3e-g
EMxylB+6GTRTT=25_F	ggtGcaattactggtgctttgttacAtgaagttgcgaaagcgtATATAAAG	Fig. 3e-g
EMxylB+6GTRTT=26_F	ggtGcaaattactggtgctttgttacAtgaagttgcgaaagcgtATATATTC	Fig. 3e-g
EMxylB+6GTRTT=27_F	ggtGcgaaattactggtgctttgttacAtgaagttgcgaaagcgtATACATCT	Fig. 3e-g
EMxylB+6GTRTT=28_F	ggtGccgaaattactggtgctttgttacAtgaagttgcgaaagcgtATACATCT	Fig. 3e-g
EMxylB+6GTRTT=29_F	ggtGcgcgaaattactggtgctttgttacAtgaagttgcgaaagcgtAAATATAT	Fig. 3e-g
EMxylB+6GTRTT=30_F	ggtGcagcgaaattactggtgctttgttacAtgaagttgcgaaagcgtATATATAG	Fig. 3e-g
EMxylB+6GTRTT=31_F	ggtGccagcgaaattactggtgctttgttacAtgaagttgcgaaagcgtATAAATAT	Fig. 3e-g
EMlacZ+6GTRTT=8_R	CGCGGCTTGTTTccgaatctctatcgtgcgTtg	Fig. 3e-g
EMlacZ+6GTRTT=9_R	CGCGATTATGTTccgaatctctatcgtgcgTtgg	Fig. 3e-g
EMlacZ+6GTRTT=10_R	CGCGTATGTGTTccgaatctctatcgtgcgTtggt	Fig. 3e-g
EMlacZ+6GTRTT=11_R	CGCGTTATGGTTccgaatctctatcgtgcgTtggtt	Fig. 3e-g
EMlacZ+6GTRTT=12_R	CGCGTATTGTTTccgaatctctatcgtgcgTtggttg	Fig. 3e-g
EMlacZ+6GTRTT=13_R	CGCGCATTGTTTccgaatctctatcgtgcgTtggttga	Fig. 3e-g
EMlacZ+6GTRTT=14_R	CGCGTCTTGTTTccgaatctctatcgtgcgTtggttgaa	Fig. 3e-g
EMlacZ+6GTRTT=15_R	CGCGTAGTTGGTccgaatctctatcgtgcgTtggttgaac	Fig. 3e-g
EMlacZ+6GTRTT=16_R	CGCGGTATGTTTccgaatctctatcgtgcgTtggttgaact	Fig. 3e-g
EMlacZ+6GTRTT=17_R	CGCGATTTGTTTccgaatctctatcgtgcgTtggttgaactg	Fig. 3e-g
EMlacZ+6GTRTT=18_R	CGCGATTTGTTTccgaatctctatcgtgcgTtggttgaactgc	Fig. 3e-g
EMlacZ+6GTRTT=19_R	CGCGTTATTGTAccgaatctctatcgtgcgTtggttgaactgca	Fig. 3e-g

EMlacZ+6GTRTT=20_R	CGCGCTTGCTGTccgaatctctatcgtgcgTtggttgaactgcac	Fig. 3e-g
EMlacZ+6GTRTT=21_R	CGCGCATTTGTTccgaatctctatcgtgcgTtggttgaactgcaca	Fig. 3e-g
EMlacZ+6GTRTT=22_R	CGCGCATTATGTccgaatctctatcgtgcgTtggttgaactgcacac	Fig. 3e-g
EMlacZ+6GTRTT=23_R	CGCGATTGTTTGccgaatctctatcgtgcgTtggttgaactgcacacc	Fig. 3e-g
EMlacZ+6GTRTT=24_R	CGCGCACTTTGTccgaatctctatcgtgcgTtggttgaactgcacaccg	Fig. 3e-g
EMlacZ+6GTRTT=25_R	CGCGACTTATGTccgaatctctatcgtgcgTtggttgaactgcacaccgc	Fig. 3e-g
EMlacZ+6GTRTT=26_R	CGCGGCATATGTccgaatctctatcgtgcgTtggttgaactgcacaccgcc	Fig. 3e-g
EMlacZ+6GTRTT=27_R	CGCGTTATTGTTccgaatctctatcgtgcgTtggttgaactgcacaccgccg	Fig. 3e-g
EMlacZ+6GTRTT=28_R	CGCGTATATGTTccgaatctctatcgtgcgTtggttgaactgcacaccgccga	Fig. 3e-g
EMlacZ+6GTRTT=29_R	CGCGGTACTGGGccgaatctctatcgtgcgTtggttgaactgcacaccgccga c	Fig. 3e-g
EMlacZ+6GTRTT=30_R	CGCGTGTTGTTGccgaatctctatcgtgcgTtggttgaactgcacaccgccga cg	Fig. 3e-g
EMlacZ+6GTRTT=31_R	CGCGGTGTTTAAccgaatctctatcgtgcgTtggttgaactgcacaccgccga cgg	Fig. 3e-g
EMrpoB+6GTRTT=8_R	CGCGGTAATTATagctgtctcagtttatgTacc	Fig. 3e-g
EMrpoB+6GTRTT=9_R	CGCGCATTATATagctgtctcagtttatgTacca	Fig. 3e-g
EMrpoB+6GTRTT=10_R	CGCGCTTAATTTagctgtctcagtttatgTaccag	Fig. 3e-g
EMrpoB+6GTRTT=11_R	CGCGTAATTTGTagctgtctcagtttatgTaccaga	Fig. 3e-g
EMrpoB+6GTRTT=12_R	CGCGATTTATGTagctgtctcagtttatgTaccagaa	Fig. 3e-g
EMrpoB+6GTRTT=13_R	CGCGATAATTATagctgtctcagtttatgTaccagaac	Fig. 3e-g
EMrpoB+6GTRTT=14_R	CGCGAAGGGATTagctgtctcagtttatgTaccagaaca	Fig. 3e-g
EMrpoB+6GTRTT=15_R	CGCGCGATTTAAagctgtctcagtttatgTaccagaacaa	Fig. 3e-g
EMrpoB+6GTRTT=16_R	CGCGGTGGAATTagctgtctcagtttatgTaccagaacaac	Fig. 3e-g
EMrpoB+6GTRTT=17_R	CGCGCGGATTTAagctgtctcagtttatgTaccagaacaacc	Fig. 3e-g
EMrpoB+6GTRTT=18_R	CGCGGTCTATATagctgtctcagtttatgTaccagaacaaccc	Fig. 3e-g
EMrpoB+6GTRTT=19_R	CGCGAATTATATagctgtctcagtttatgTaccagaacaacccg	Fig. 3e-g
EMrpoB+6GTRTT=20_R	CGCGATGTTAATagctgtctcagtttatgTaccagaacaacccgc	Fig. 3e-g
EMrpoB+6GTRTT=21_R	CGCGCGATCTATagctgtctcagtttatgTaccagaacaacccgct	Fig. 3e-g
EMrpoB+6GTRTT=22_R	CGCGTTTGTTATagctgtctcagtttatgTaccagaacaacccgctg	Fig. 3e-g
EMrpoB+6GTRTT=23_R	CGCGTATATTATagctgtctcagtttatgTaccagaacaacccgctgt	Fig. 3e-g
EMrpoB+6GTRTT=24_R	CGCGGTCTAATTagctgtctcagtttatgTaccagaacaacccgctgtc	Fig. 3e-g
EMrpoB+6GTRTT=25_R	CGCGATATTATTagctgtctcagtttatgTaccagaacaacccgctgtct	Fig. 3e-g
EMrpoB+6GTRTT=26_R	CGCGATGTTATTagctgtctcagtttatgTaccagaacaacccgctgtctg	Fig. 3e-g
EMrpoB+6GTRTT=27_R	CGCGATCTATATagctgtctcagtttatgTaccagaacaacccgctgtctga	Fig. 3e-g
EMrpoB+6GTRTT=28_R	CGCGGTATAATTagctgtctcagtttatgTaccagaacaacccgctgtctgag	Fig. 3e-g
EMrpoB+6GTRTT=29_R	CGCGTATAATTTagctgtctcagtttatgTaccagaacaacccgctgtctgaga	Fig. 3e-g
EMrpoB+6GTRTT=30_R	CGCGATATTTATagctgtctcagtttatgTaccagaacaacccgctgtctgagat	Fig. 3e-g
EMrpoB+6GTRTT=31_R	CGCGGTATTTATagctgtctcagtttatgTaccagaacaacccgctgtctgagatt	Fig. 3e-g
EMxylB+6GTRTT=8_R	CGCGTTAATTGAacgctttcgcaacttcaTgta	Fig. 3e-g
EMxylB+6GTRTT=9_R	CGCGCTATTTGAacgctttcgcaacttcaTgtaa	Fig. 3e-g
EMxylB+6GTRTT=10_R	CGCGTATATATGacgctttcgcaacttcaTgtaac	Fig. 3e-g
EMxylB+6GTRTT=11_R	CGCGGATCTTGAacgctttcgcaacttcaTgtaaca	Fig. 3e-g
EMxylB+6GTRTT=12_R	CGCGGAGTATGAacgctttcgcaacttcaTgtaacaa	Fig. 3e-g
EMxylB+6GTRTT=13_R	CGCGCTCTAATGacgctttcgcaacttcaTgtaacaaa	Fig. 3e-g
EMxylB+6GTRTT=14_R	CGCGTTGGTATGacgctttcgcaacttcaTgtaacaaag	Fig. 3e-g
EMxylB+6GTRTT=15_R	CGCGACTCTAGGacgctttcgcaacttcaTgtaacaaagc	Fig. 3e-g
EMxylB+6GTRTT=16_R	CGCGGTTGATAAacgctttcgcaacttcaTgtaacaaagca	Fig. 3e-g

EMxylB+6GTRTT=17_R	CGCGACTCGGGAacgctttcgcaacttcaTgtaacaaagcac	Fig. 3e-g
EMxylB+6GTRTT=18_R	CGCGTCTATTATacgctttcgcaacttcaTgtaacaaagcacc	Fig. 3e-g
EMxylB+6GTRTT=19_R	CGCGGGATATTTacgctttcgcaacttcaTgtaacaaagcacca	Fig. 3e-g
EMxylB+6GTRTT=20_R	CGCGATTTATTTacgctttcgcaacttcaTgtaacaaagcaccag	Fig. 3e-g
EMxylB+6GTRTT=21_R	CGCGGGATGTATacgctttcgcaacttcaTgtaacaaagcaccagt	Fig. 3e-g
EMxylB+6GTRTT=22_R	CGCGGGATATTTacgctttcgcaacttcaTgtaacaaagcaccagta	Fig. 3e-g
EMxylB+6GTRTT=23_R	CGCGGTGTGTAAacgctttcgcaacttcaTgtaacaaagcaccagtaa	Fig. 3e-g
EMxylB+6GTRTT=24_R	CGCGCAATATTTacgctttcgcaacttcaTgtaacaaagcaccagtaat	Fig. 3e-g
EMxylB+6GTRTT=25_R	CGCGCTTTATATacgctttcgcaacttcaTgtaacaaagcaccagtaatt	Fig. 3e-g
EMxylB+6GTRTT=26_R	CGCGGAATATATacgctttcgcaacttcaTgtaacaaagcaccagtaattt	Fig. 3e-g
EMxylB+6GTRTT=27_R	CGCGAGATGTATacgctttcgcaacttcaTgtaacaaagcaccagtaatttc	Fig. 3e-g
EMxylB+6GTRTT=28_R	CGCGAGATGTATacgctttcgcaacttcaTgtaacaaagcaccagtaatttcg	Fig. 3e-g
EMxylB+6GTRTT=29_R	CGCGATATATTTacgctttcgcaacttcaTgtaacaaagcaccagtaatttcgc	Fig. 3e-g
EMxylB+6GTRTT=30_R	CGCGCTATATATacgctttcgcaacttcaTgtaacaaagcaccagtaatttcgct	Fig. 3e-g
EMxylB+6GTRTT=31_R	CGCGATATTTATacgctttcgcaacttcaTgtaacaaagcaccagtaatttcgctg	Fig. 3e-g
pegRNA2_tevopreQ1galK +5G-to-T_F	ggtGcgcagtttcAatgcCCAAAGGTGTGGCAAATATTC	Fig. 3hFig. 4a
pegRNA2_tevopreQ1galK +5TTAA ins_F	ggtGcgcagtttccTTAAatgcCCAAAGGTGTGGCACAATATT	Fig. 3hFig. 4a
pegRNA2_tevopreQ1galK +4-6 TGG del_F	ggtGcaaagcagttttgcCCAAAGGTGTGGCACAGGAAT	Fig. 3hFig. 4a
pegRNA2_tevopreQ1pta+5 G-to-T_F	ggtGcaagtgctcAagcaTGTGCGGAATGCTATCAAATT	Fig. 3hFig. 4a
pegRNA2_tevopreQ1pta+5 TTAA ins_F	ggtGcaagtgctccTTAAagcaTGTGCGGAATGCTAATAGATC	Fig. 3hFig. 4a
pegRNA2_tevopreQ1pta+4 -6 TGG del_F	ggtGccggaagtgctgcaTGTGCGGAATGCTATAAACAA	Fig. 3hFig. 4a
pegRNA2_tevopreQ1xylB +5G-to-T_F	ggtGctttgttacAtgaaGTTGCGAAAGCGTTCAATCAG	Fig. 3hFig. 4a
pegRNA2_tevopreQ1xylB +5TTAA ins_F	ggtGctttgttaccTTAAtgaaGTTGCGAAAGCGTCATATATA	Fig. 3hFig. 4a
pegRNA2_tevopreQ1xylB +4-6 AGG del_F	ggtGctgctttgttagaaGTTGCGAAAGCGTCACACCGA	Fig. 3hFig. 4a
pegRNA2_tevopreQ1adhE +5G-to-T_F	ggtGccggagcacAggcaGCGATAGCAGCCTAATATTGA	Fig. 3hFig. 4a
pegRNA2_tevopreQ1adhE +5TTAA ins_F	ggtGccggagcaccTTAAggcaGCGATAGCAGCCTAACTATAA	Fig. 3hFig. 4a
pegRNA2_tevopreQ1adhE +4-6 CGG del_F	ggtGctttcggagcagcaGCGATAGCAGCCTAATACCAA	Fig. 3hFig. 4a
pegRNA2_tevopreQ1galK +5G-to-T_R	CGCGGAATATTTgccacacctttgggcatTgaaactgc	Fig. 3hFig. 4a
pegRNA2_tevopreQ1galK +5TTAA ins_R	CGCGAATATTGTgccacacctttgggcatTTAAggaaactgc	Fig. 3hFig. 4a
pegRNA2_tevopreQ1galK +4-6 TGG del_R	CGCGATTCCTGTgccacacctttgggcaaaactgc	Fig. 3hFig. 4a
pegRNA2_tevopreQ1pta+5 G-to-T_R	CGCGAATTTGATagcattccgcacatgctTgagcactt	Fig. 3hFig. 4a
pegRNA2_tevopreQ1pta+5 TTAA ins_R	CGCGGATCTATTagcattccgcacatgctTTAAggagcactt	Fig. 3hFig. 4a

pegRNA2_tevopreQ1pta+4-6 TGG del_R	CGCGTTGTTTATagcattccgcacatgcagcactt	Fig. 3hFig. 4a
pegRNA2_tevopreQ1xylB+5G-to-T_R	CGCGCTGATTGAacgctttcgcaacttcaTgtaacaaa	Fig. 3hFig. 4a
pegRNA2_tevopreQ1xylB+5TTAA ins_R	CGCGTATATATGacgctttcgcaacttcaTTAAggtaacaaa	Fig. 3hFig. 4a
pegRNA2_tevopreQ1xylB+4-6 AGG del_R	CGCGTCGGTGTGacgctttcgcaacttctaacaaa	Fig. 3hFig. 4a
pegRNA2_tevopreQ1adhE+5G-to-T_R	CGCGTCAATATTaggctgctatcgctgccTgtgctccg	Fig. 3hFig. 4a
pegRNA2_tevopreQ1adhE+5TTAA ins_R	CGCGTTATAGTTaggctgctatcgctgccTTAAggtgctccg	Fig. 3hFig. 4a
pegRNA2_tevopreQ1adhE+4-6 CGG del_R	CGCGTTGGTATTaggctgctatcgctgctgctccg	Fig. 3hFig. 4a
pegRNA2_tevopreQ1lacZ+5G-to-T_F	ggtGctcaaccacAgcacGATAGAGATTCGGAAACAAAT	Fig. 3h
pegRNA2_tevopreQ1lacZ+5TTAA ins_F	ggtGctcaaccaccTTAAgcacGATAGAGATTCGGACCCACAT	Fig. 3h
pegRNA2_tevopreQ1lacZ+4-6 CGG del_F	ggtGcagttcaaccacacGATAGAGATTCGGAAACATAA	Fig. 3h
pegRNA2_tevopreQ1lacZ+5G-to-T_R	CGCGATTTGTTTccgaatctctatcgtgcTgtggttga	Fig. 3h
pegRNA2_tevopreQ1lacZ+5TTAA ins_R	CGCGATGTGGGTccgaatctctatcgtgcTTAAggtggttga	Fig. 3h
pegRNA2_tevopreQ1lacZ+4-6 CGG del_R	CGCGTTATGTTTccgaatctctatcgtgtggttga	Fig. 3h
pyrF_T1_F	TAGTTGATAATCGCGATAAAGCGCGTTTT	Fig. 4b
pyrF_T2_F	TAGTCATGGAGTCCAGCGACCTGCGTTTT	Fig. 4b
fosA_F	TAGTCTGCTTCGCCGCTCGTCTTGGTTTT	Fig. 4b
ramA_T1_F	TAGTGATATCGTAGACCCGCTGATGTTTT	Fig. 4b
pyrF_T1_R	gctctaaaacGCGCTTTATCGCGATTATCA	Fig. 4b
pyrF_T2_R	gctctaaaacGCAGGTCGCTGGACTCCATG	Fig. 4b
fosA_R	gctctaaaacCAAGACGAGCGGCGAAGCAG	Fig. 4b
ramA_T1_R	gctctaaaacATCAGCGGGTCTACGATATC	Fig. 4b
pegRNA2_tevopreQ1pyrF_T1pyrF+6G-to-T_F	ggtGcACAAATGACAGCGCTTTATCGCGATTTGAAACCG	Fig. 4b
pegRNA2_tevopreQ1pyrF_T1pyrF+5TTAA ins_F	ggtGcACAAATGCCttaaAGCGCTTTATCGCGATTTGACCTAT	Fig. 4b
pegRNA2_tevopreQ1pyrF_T1pyrF+4-6 TGG del_F	ggtGcTCGACAAATGGCGCTTTATCGCGATTTGAACTAG	Fig. 4b
pegRNA2_tevopreQ1pyrF_T2pyrF+6G-to-T_F	ggtGcCCCAGATACTGCAGGTCGCTGGACTCAAACTAAA	Fig. 4b
pegRNA2_tevopreQ1pyrF_T2pyrF+5TTAA ins_F	ggtGcCCCAGATCCTTAATGCAGGTCGCTGGACTCAACTCCCG	Fig. 4b
pegRNA2_tevopreQ1pyrF_T2pyrF+4-6 AGG del_F	ggtGcATGCCCAGATGCAGGTCGCTGGACTCAACTTATT	Fig. 4b
pegRNA2_tevopreQ1fosAfosA+6G-to-T_F	ggtGcCCGGCAAACTCAAGACGAGCGGCGAATTTAAACA	Fig. 4b
pegRNA2_tevopreQ1fosAf	ggtGcCCGGCAACCTTAATCAAGACGAGCGGCGAATTTA	Fig. 4b

osA+5TTAA ins_F	ACCT	
pegRNA2_tevopreQ1fosAfosA+4-6 AGG del_F	ggtGcACGCCGGCAACAAGACGAGCGGCGAAATTAAACA	Fig. 4b
pegRNA2_tevopreQ1ramA_T1ramA+5G-to-T_F	ggtGcCGCGACACAGATCAGCGGGTCTACGAAACAAATG	Fig. 4b
pegRNA2_tevopreQ1ramA_T1ramA+5TTAA ins_F	ggtGcCGCGACACCTTAAGATCAGCGGGTCTACGAGGAAAGGC	Fig. 4b
pegRNA2_tevopreQ1ramA_T1ramA+4-6 CGG del_F	ggtGcCTGCGCGACAATCAGCGGGTCTACGACGCAGAAT	Fig. 4b
pegRNA2_tevopreQ1pyrF_T1pyrF+6G-to-T_R	CGCGCGGTTTCAAATCGCGATAAAGCGCTGtCATTTGT	Fig. 4b
pegRNA2_tevopreQ1pyrF_T1pyrF+5TTAA ins_R	CGCGATAGGTCAAATCGCGATAAAGCGCTttaaGGCATTTGT	Fig. 4b
pegRNA2_tevopreQ1pyrF_T1pyrF+4-6 TGG del_R	CGCGCTAGTTCAAATCGCGATAAAGCGCCATTTGTCGA	Fig. 4b
pegRNA2_tevopreQ1pyrF_T2pyrF+6G-to-T_R	CGCGTTTAGTTTGAGTCCAGCGACCTGCAGtATCTGGG	Fig. 4b
pegRNA2_tevopreQ1pyrF_T2pyrF+5TTAA ins_R	CGCGCGGGAGTTGAGTCCAGCGACCTGCATTAAGGATCTGGG	Fig. 4b
pegRNA2_tevopreQ1pyrF_T2pyrF+4-6 AGG del_R	CGCGAATAAGTTGAGTCCAGCGACCTGCATCTGGGCAT	Fig. 4b
pegRNA2_tevopreQ1fosAfosA+6G-to-T_R	CGCGTGTTTAAATTCGCCGCTCGTCTTGAGtTTGCCGG	Fig. 4b
pegRNA2_tevopreQ1fosAfosA+5TTAA ins_R	CGCGAGGTTAAATTCGCCGCTCGTCTTGATTAAGGTTGCCGG	Fig. 4b
pegRNA2_tevopreQ1fosAfosA+4-6 AGG del_R	CGCGTGTTTAATTTCGCCGCTCGTCTTGTTGCCGGCGT	Fig. 4b
pegRNA2_tevopreQ1ramA_T1ramA+5G-to-T_R	CGCGCATTTGTTTCGTAGACCCGCTGATCtGTGTCGCG	Fig. 4b
pegRNA2_tevopreQ1ramA_T1ramA+5TTAA ins_R	CGCGGCCTTTCCTCGTAGACCCGCTGATCTTAAGGTGTCGCG	Fig. 4b
pegRNA2_tevopreQ1ramA_T1ramA+4-6 CGG del_R	CGCGATTCTGCGTCGTAGACCCGCTGATTGTCGCGCAG	Fig. 4b
GGA_KO_KP_sbcB_4_F	tagtGTGCCGCAAACCTGCATCGT	Fig. 4b
GGA_KO_KP_xseA_2_F	tagtAACTTCATCCAGCCTTCTTC	Fig. 4b
GGA_KO_KP_exoX_2_F	tagtCACCAATCCCATGAGTCATC	Fig. 4b
GGA_KO_KP_sbcB_4_R	aaacACGATGCAGGTTTGCGGCAC	Fig. 4b
GGA_KO_KP_xseA_2_R	aaacGAAGAAGGCTGGATGAAGTT	Fig. 4b
GGA_KO_KP_exoX_2_R	aaacGATGACTCATGGGATTGGTG	Fig. 4b
aceI_T1_F	TAGTgagcggtcaattgaacccaaGTTTT	Fig. 4c
aceI_T2_F	TAGTcttgggatgcaaatgcttcaGTTTT	Fig. 4c
adeB_T1_F	TAGTactattccgttgtctgctcaGTTTT	Fig. 4c
cpdA_T1_F	TAGTcatcaacaacctgtagttgtGTTTT	Fig. 4c
cpdA_T2_F	TAGTgattcccttaatatctggcaGTTTT	Fig. 4c
cpdA_T3_F	TAGTtacattcgtctgaataacgaGTTTT	Fig. 4c
entE_T1_F	TAGTtgcaggtggatgtgttgtgaGTTTT	Fig. 4c
aceI_T1_R	gctctaaaacttgggttcaattgaccgctc	Fig. 4c
aceI_T2_R	gctctaaaactgaagcatttgcatcccaag	Fig. 4c
adeB_T1_R	gctctaaaactgagcagacaacggaatagt	Fig. 4c

cpdA_T1_R	gctctaaaacacaactacaggttgttgatg	Fig. 4c
cpdA_T2_R	gctctaaaactgccagatattaagggaatc	Fig. 4c
cpdA_T3_R	gctctaaaactcgttattcagacgaatgta	Fig. 4c
entE_T1_R	gctctaaaactcacaacacatccacctgca	Fig. 4c
pegRNA2_tevopreQ1aceI_T1aceI_T1+5G_to_T_F	ggtGcagatgcacAattgggttcaattgaccAAAGATAG	Fig. 4c
pegRNA2_tevopreQ1aceI_T2aceI_T2+5G_to_T_F	ggtGcacgaaatcAatgaagcatttgcatccATCTACGT	Fig. 4c
pegRNA2_tevopreQ1adeB_T1adeB_T1+5G_to_T_F	ggtGccaattgccAttgagcagacaacggaaCCTTAAAC	Fig. 4c
pegRNA2_tevopreQ1cpdA_T1cpdA_T1+5G_to_T_F	ggtGctcctaaacAgacaactacaggttgttACAATTCT	Fig. 4c
pegRNA2_tevopreQ1cpdA_T2cpdA_T2+5G_to_T_F	ggtGcctcgatacAttgccagatattaagggCCCAAAGG	Fig. 4c
pegRNA2_tevopreQ1cpdA_T3cpdA_T3+5G_to_T_F	ggtGcaaaactacAatcgttattcagacgaaCCTTATTA	Fig. 4c
pegRNA2_tevopreQ1entE_T1entE_T1+5G_to_T_F	ggtGctttggtgcAatcacaacacatccaccCCTTCTAG	Fig. 4c
pegRNA2_tevopreQ1aceI_T1aceI_T1+5G_to_T_R	CGCGCTATCTTTggtcaattgaacccaatTgtgcatct	Fig. 4c
pegRNA2_tevopreQ1aceI_T2aceI_T2+5G_to_T_R	CGCGACGTAGATggatgcaaatgcttcatTgatttcgt	Fig. 4c
pegRNA2_tevopreQ1adeB_T1adeB_T1+5G_to_T_R	CGCGGTTTAAGGttccgttgtctgctcaaTggcaattg	Fig. 4c
pegRNA2_tevopreQ1cpdA_T1cpdA_T1+5G_to_T_R	CGCGAGAATTGTaacaacctgtagttgtcTgtttagga	Fig. 4c
pegRNA2_tevopreQ1cpdA_T2cpdA_T2+5G_to_T_R	CGCGCCTTTGGGcccttaatatctggcaaTgtatcgag	Fig. 4c
pegRNA2_tevopreQ1cpdA_T3cpdA_T3+5G_to_T_R	CGCGTAATAAGGttcgtctgaataacgatTgtagtttt	Fig. 4c
pegRNA2_tevopreQ1entE_T1entE_T1+5G_to_T_R	CGCGCTAGAAGGggtggatgtgttgtgatTgcaccaaa	Fig. 4c
GGA_KO_Ab_xseA_3_F	tagtcctcaaaatgctgcgggttt	Fig. 4c
GGA_KO_Ab_exoX_1_F	tagttttgatcaactttatcaagt	Fig. 4c
GGA_KO_Ab_xseA_3_R	aaacaaacccgcagcattttgagg	Fig. 4c
GGA_KO_Ab_exoX_1_R	aaacacttgataaagttgatcaaa	Fig. 4c
CRISPRi_GGA_sbcB_T1_F	GTAGATacctaatgatgaatgacggtaagTTTTTTT	Sup.10
CRISPRi_GGA_sbcB_T2_F	GTAGATacgattacgaaacctttggcacgTTTTTTT	Sup.10
CRISPRi_GGA_xseA_T1_F	GTAGATatctcgctcacatgttaccttctTTTTTTT	Sup.10
CRISPRi_GGA_xseA_T2_F	GTAGATacgcaaccagcttccggtcactgTTTTTTT	Sup.10
CRISPRi_GGA_exoX_T1_F	GTAGATcagggagggatcgttgagattgcTTTTTTT	Sup.10
CRISPRi_GGA_exoX_T2_F	GTAGATtggcctgggatcaagtacagcaaTTTTTTT	Sup.10
CRISPRi_GGA_sbcB_T1_	cttcaaaaaaacttaccgtcattcatcattaggtAT	Sup.10

R		
CRISPRi_GGA_sbcB_T2_R	cttcaaaaaaacgtgccaaaggtttcgtaatcgtAT	Sup.10
CRISPRi_GGA_xseA_T1_R	cttcaaaaaaaagaaggtaacatgtgagcgagatAT	Sup.10
CRISPRi_GGA_xseA_T2_R	cttcaaaaaaacagtgaccggaagctggttgcgtAT	Sup.10
CRISPRi_GGA_exoX_T1_R	cttcaaaaaaagcaatctcaacgatccctccctgAT	Sup.10
CRISPRi_GGA_exoX_T2_R	cttcaaaaaaattgctgtacttgatcccaggccaAT	Sup.10
pBbS8c-ddcpf1@GGAmethod_R	AGATGCGggtctcGctacaagagtagaaattgaaattgttatccgctcac	Sup.10
pBbS8c-ddcpf1@GGAmethod_F	CGCATCTggtctCagaagcttgggcccgaacaaaaactc	Sup.10
MS_PE_1	aagctggacttcAGCGGCGGTTCTTCCGGCGG	MS_PE
MS_PE_2	AGAACCGCCGCTgaagtccagcttcggcttgt	MS_PE
MS_PE_3	ctcactcaaaggTGGCTCACGCAAAAACAACGAACCACAC	MS_PE
MS_PE_4	TTGCGTGAGCCAcctttgagtgagctgatacc	MS_PE
MS_PE_5	atcctcatcggtggctcttcctcccagattatatctatcactgatagggatcgcaaatc	MS_PE
MS_PE_6	gaagagccaccgatgaggatctcgatccgctcttcgTttttttaatacggttatccac	MS_PE
MS_PE_7	gggcctggAcatcggcatcggctc	MS_PE
MS_PE_8	tgccgatgTccaggcccagcacc	MS_PE
MS_PE_Cb_1	acgttgtgtctcgtcgcaaacgctgtttgctcc	MS_PE_Cb
MS_PE_Cb_2	agcgtttgcgacgagacacaacgtggctttccc	MS_PE_Cb
MS_PE_Cb_3	gttggtgagagcggaaatgtgcgcggaacccc	MS_PE_Cb
MS_PE_Cb_4	cgcgcacatttccgctctcaccaaccgtggctc	MS_PE_Cb
PEE1_1	taaggaggttataaaaaatggataagaaatactcaataggcttagatatc	SpPE
PEE1_2	cctattgagtatttcttatccattttttataacctccttagagctcgaattc	SpPE
PEE1_3	ccatgggtatggTTTCCATAGGCTCCGCCCCC	SpPE
PEE1_4	AGCCTATGGAAAccatacccatggattcttcgtctg	SpPE
PEE1_5	AAACGATCTCAAgtcgcaaacgctgtttgctc	SpPE
PEE1_6	agcgtttgcgacTTGAGATCGTTTTGGTCTGCGCG	SpPE
PEE1_7	aagttgtcataataaatcgatgcaggtggcac	SpPE
PEE1_8	tgcatcgatttattatgacaacttgacggcta	SpPE
epegRNA_accepter_1	aagcttgatatcACGCTGTGGAAGTTGACAGC	epegRNA_accepter
epegRNA_accepter_2	CTTCCACAGCGTgatatcaagcttgcaactttatg	epegRNA_accepter
epegRNA_accepter_3	attcattaatgcACCTCGATACCCAAAAAACC	epegRNA_accepter
epegRNA_accepter_4	GGGTATCGAGGTgcattaatgaatcggccaac	epegRNA_accepter
epegRNA_accepter_5	GCGATCGTCTCGGACTGAGCTAGCTGTCAAaaagggcctcgtgatacgcc	epegRNA_accepter
epegRNA_accepter_6	GCTCAGTCCGAGACGATCGCTACGTCTCAtcacagcttgtctgtaagcgg	epegRNA_accepter
Ab_PE_1	tttcttatccatgaattctgtttcctgtgtgaaattgttatc	Ab_PE
Ab_PE_2	gaaacagaattcatggataagaaatactcaataggcttag	Ab_PE
Ab_PE_3	tacgccaaccagccatacccatggattcttcgtctg	Ab_PE
Ab_PE_4	ccatgggtatggctggttggcgtactgttgtg	Ab_PE
KP_PE_1	gccgatcaacgtcacatttccccgaaaagtgc	KP_PE
KP_PE_2	aggtagtcggcactgtcagaccaagtttactc	KP_PE

KP_PE_3	ttggtctgacagtgccgactaccttggtgatctc	KP_PE
KP_PE_4	cggggaaatgtgacgttgatcggcacgtaagag	KP_PE
pBbS8c_ddCpf1_final_1	tcacatggactaccggatctgcgattctgata	pBbS8c_ddCpf1_final
pBbS8c_ddCpf1_final_2	tcgcagatccggtagtccatgtgaccttcaccc	pBbS8c_ddCpf1_final
pBbS8c_ddCpf1_final_3	ctggtgttctcgcaggctgtctatgtgtgactg	pBbS8c_ddCpf1_final
pBbS8c_ddCpf1_final_4	atagacagcctgcgagaacaccagaacagccc	pBbS8c_ddCpf1_final

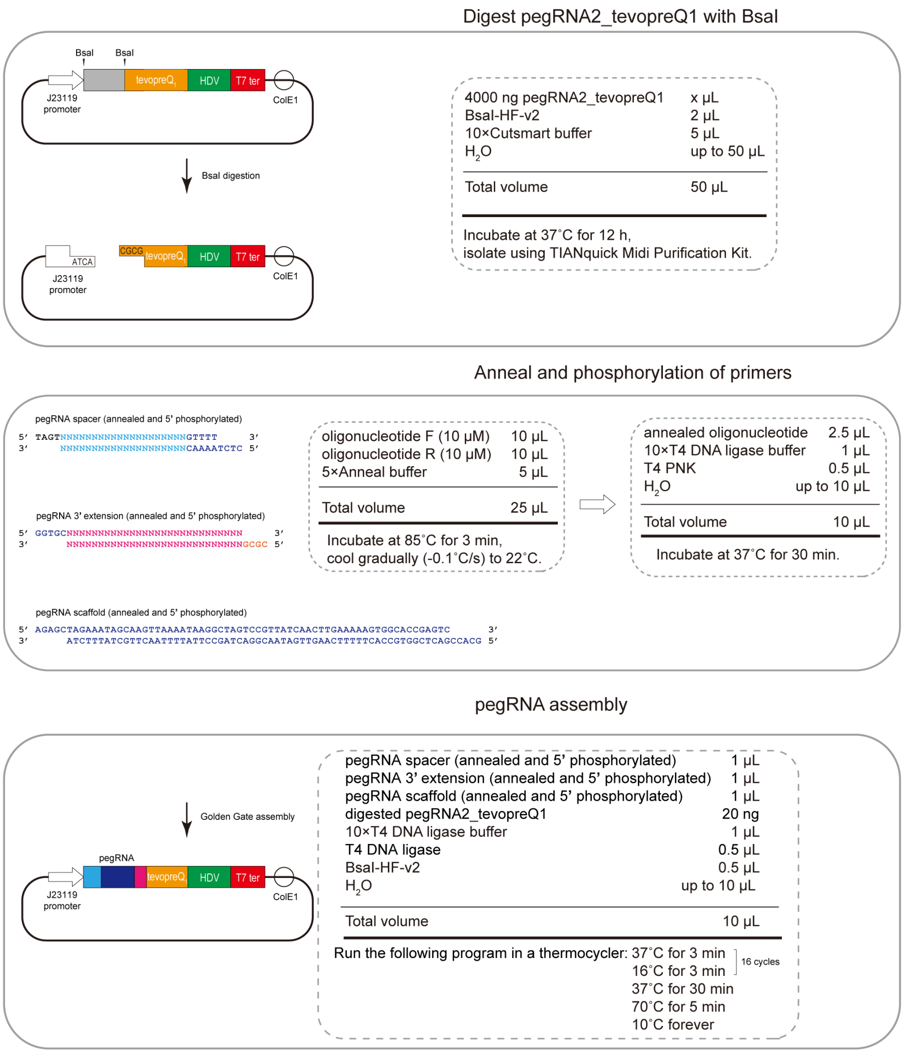

Supplementary Note 1. **Overview of pegRNA cloning protocol.**

References

1. Baba T*, et al.* Construction of Escherichia coli K-12 in-frame, single-gene knockout mutants: the Keio collection. *Molecular Systems Biology* **2**, 2006.0008 (2006).

2. Jiang W, Bikard D, Cox D, Zhang F, Marraffini LA. RNA-guided editing of bacterial genomes using CRISPR-Cas systems. *Nature Biotechnology* **31**, 233-239 (2013).

3. Jervis AJ, Hanko EKR, Dunstan MS, Robinson CJ, Takano E, Scrutton NS. A plasmid toolset for CRISPR-mediated genome editing and CRISPRi gene regulation in Escherichia coli. *Microbial Biotechnology* **14**, 1120-1129 (2021).

4. Wang Y, Wang Z, Chen Y, Hua X, Yu Y, Ji Q. A Highly Efficient CRISPR-Cas9-Based Genome Engineering Platform in Acinetobacter baumannii to Understand the H2O2-Sensing Mechanism of OxyR. *Cell Chemical Biology* **26**, 1732-1742.e1735 (2019).

5. Wang Y*, et al.* CRISPR-Cas9 and CRISPR-Assisted Cytidine Deaminase Enable Precise and Efficient Genome Editing in Klebsiella pneumoniae. *Applied and Environmental Microbiology* **84**, e01834-01818 (2018).
