# Supplementary material for: BacPE: a versatile prime-editing platform in bacteria by inhibiting DNA exonucleases
Source: Nat Commun. 2024 Jan 27;15:825. doi: 10.1038/s41467-024-45114-4 (PMC10821919; doi:10.1038/s41467-024-45114-4)
Supplement: Supplementary file 3 — Peer Review File [file 41467_2024_45114_MOESM3_ESM.pdf]

nature portfolio

Peer Review File

BacPE: a versatile prime-editing platform in bacteria by inhibiting DNA exonucleases

Open Access This file is licensed under a Creative Commons Attribution 4.0 International License, which permits use, sharing, adaptation, distribution and reproduction in any medium or format, as long as you give appropriate credit to the original author(s) and the source, provide a link to the Creative Commons license, and indicate if changes were made. In the cases where the authors are anonymous, such as is the case for the reports of anonymous peer reviewers, author attribution should be to 'Anonymous Referee' followed by a clear attribution to the source work. The images or other third party material in this file are included in the article's Creative Commons license, unless indicated otherwise in a credit line to the material. If material is not included in the article's Creative Commons license and your intended use is not permitted by statutory regulation or exceeds the permitted use, you will need to obtain permission directly from the copyright holder. To view a copy of this license, visit http://creativecommons.org/licenses/by/4.0/.

Reviewers' Comments:

Reviewer #1:
Remarks to the Author:
In this manuscript, the authors explored the applications of prime editing in bacteria. Specifically, through screening 129 Keio collection of E. coli mutants, they identified that 3′-5′ DNA exonucleases sbcB, xseA, and exoX are the key factors that inhibit prime editing in several bacterial species. Consistently, deleting these genes or repressing their expression through CRISPRi enabled prime editing in several bacterial strains. Based on this, a 3′-directed hydrolysis model for inhibiting prime editing in bacteria was proposed. This work is original and impressive. I have a few concerns.

Major points:
1. There have been various CRISPR-Cas9, Cas12, and native type I Cascade-mediated genome editing established in prokaryotes. How necessary is it to develop prime editing in bacteria? This point was not well justified in the manuscript.

2. Related to point 1, are there bacterial species/ strains and/or the types of editing in which existing editing strategies and approaches are incapable of and prime editing could achieve? The authors should show some examples of such applications. Alternatively, as a methodology manuscript, applications of the developed technique in representative Gram-positive and Gram-negative bacterial species should be shown.

3. The authors proposed a 3′-directed hydrolysis model for inhibiting prime editing in bacteria just based on the fact that deletion of 3′-5′ exonuclease genes improved prime editing. It would better to provide more direct, biochemical evidence for this mechanism.

Minor points:
1. Authors should provide schematic diagrams for the types of editing mentioned in the manuscript, e.g. in the Figure 1, schematic diagrams for the types of editing as depicted in the X-axises should be provided.
2. Overall, the figure legends are too simple. Some necessary details should be provided in the legends so that readers can understand the figures with necessary details without going through the materials and methods section.
3. What does MMR represent? It should be spelled the first time it appears in the text.
4. Legend of Fig 2 c-g, " in at different targeting loci"

Reviewer #2:
Remarks to the Author:
In this work, Zhang and colleagues show that repressing or deleting exonucleases involved in DNA repair can radically boost prime editing in bacteria. After finding that prime editing is highly efficient in M. smegmatis, they began screening for DNA repair factors potentially accounting for poor editing in E. coli. This screen uncovered sbcB, a 3′-to-5′ DNA exonuclease, whose deletion boosted prime editing in E. coli across target sites. Further deleting two other known DNA endonucleases (xseA, exoX) further boosted prime editing. Engineering the pegRNA based on prior work further enhanced prime editing, in some cases exceeding 80%. Adopting CRISPRi to repress these three genes allowed enhanced editing in a WT strain of E. coli, while deleting some of these genes in Klebsiella and Acinetobacter also enhanced prime editing across sites. Finally, the authors present a model of how these exonucleases interfere with prime editing.

To my knowledge, prime editing in bacteria has only been reported in one publication (Tong et al., Nat Commun 2021), but with poor editing efficiencies at genomic sites, hindering its adoption by the bacterial community. This work makes an important advance by identifying and removing the responsible barriers, which could open up broad use of prime editing in bacteria and open a range of editing options for fundamental research and strain engineering. The authors further provide extensive datasets, and the manuscript was well-written and should be easily accessible to a broad audience. I do have some important comments about the toxicity/escape as well as claims in the

abstract and reproducibility, although I see these only strengthening the work.

Major comments:

1. While the authors provide extensive editing data, the impact of CFUs compared to non-targeting conditions would be incredibly important to report. Targeting, particularly in the absence of repair proteins, could be highly cytotoxic, with few surviving cells. Such a condition would also select for escape phenotypes such as disruption of the Cas9 or the pegRNA or an unintended edit at the target site. While I don't expect the authors to provide this information for all target sites, reporting relative CFUs and looking at whether non-edited cells are escapers for a few representative examples would be incredibly important to potential users.

2. While the main text generally contains conclusions that nicely match the presented data, the abstract takes a number of liberties that need to be rephrased. Specifically,

- "Here, we report….": inhibition was only shown for E. coli, so this should indicate inhibition or deletion.
- "Comparative prime editing….": The comparison was only between M. smegmatis and E. coli, so better to state these two species.
- "Genetic screening of….": It would be more pertinent to state the number of repair genes that were screened rather than the number of transposons tested. If the number is smaller, then it's reasonable to drop the specific number.
- "We propose a 3′-directed….": I would recommend rewording this to indicate that disrupting related exonucleases enhanced prime editing rather than claiming the model holds. Otherwise, there is a much higher bar to claim a mechanistic model spanning different bacterial strains.
- "Efficient prime editing can be achieved….": CRISPRi was only shown in E. coli, so it remains to be shown that this same approach can greatly boost prime editing in other bacteria.

3. I could not find any description of the number of replicates or the nature of the replicates. In most cases, the error bars were extremely small, raising some concerns how independent the replicates were and how reproducible the overall results are.

4. The authors note that the ddCas12a silencing construct represses the target genes by ~80% individually. How much is repression when the genes are combined? Individual values normally decrease as multiplexing increases.

Other comments:

5. L. 73-77: the original demonstration of prime editing in E. coli was able to achieve chromosomal editing, albeit at very low levels. Therefore, some rewording is needed here to better capture what was previously demonstrated.

6. L. 118: For comparisons of the WT and ΔmutS (Ext. Data Fig. 6), the authors write that mutS deletions have minimal or no impact on the editing efficiency, but at least for the adhE target site it looks like it could have an effect. Including statistical tests with the corresponding p-values would be helpful.

7. L. 122: how many genes were screened as part of the transposon set? It would also be helpful for these genes to be listed in the SI.

8. L. 128-130: The transition from the prior sentence is abrupt. I recommend first stating that sbcB emerged as the top hit in the screens.

9. L. 134: For comparisons of the contribution of single- or combined knockouts of the 3′→5′-DNA exonucleases on the prime editing efficiency (e.g., Fig. 2c-g), are the differences significant from the WT values? Here the authors could include statistical tests and add the p-values to the main text.

10. L. 139: Which genes were deleted? Listing these here or in the SI would be helpful.

11. L. 147: Looking at Fig. S9, the edit made to xylB yields increased editing when sbcB and other repair genes were deleted. Why is there an exception, and can the authors reword the sentence to capture this?

12. L. 210: Replace "significant" with "substantial" since no statistical comparisons are being made.

13. Fig 2a: Write genes following standard nomenclature for bacteria. Also change "scrap" to "scrape".

14. Fig. 2b: based on the depicted assay, the axes should indicate the colony ratio rather than the mutation occurrence, as the authors did not directly assess the frequency of this edit.

15. Fig. 2c-g: what are the colored bars meant to represent?

16. Fig. 2h: for the middle arrow, can an intermediate step be added to show gap filling?

17. I recommend making the plasmids available on Addgene upon publication of the work to ensure the approach can be broadly disseminated. Providing annotated plasmid maps for key constructs such as through Benchling would also ease adoption.

18. The amplicon sequencing data should be made publicly available.

19. The authors could discuss in more detail the importance of investigating redundancy of gene functions in repair pathways as important finding. The authors could also provide a short statement on how prime editing in the field of microbiology could be of advantage in comparison to already established CRISPR-Cas gene editing technologies.

Reviewer #3:
Remarks to the Author:
This manuscript written by Zhang H et al describes the development of a practical prime editing method, which is useful for bacterial fine genome editing. The authors wanted to provide a useful prime editing method for E. coli and found that Exonuclease I (the sbcC gene product) is critical for reducing the efficiency in E. coli cells. Furthermore, additional mutations of xseA and exoX drastically enhanced the prime editing efficiency (up to 100-fold). From these experimental results, the authors proposed a model of the 3'-directed hydrolysis for degradation of the prime editing intermediates to explain inhibition of prime editing in Bacteria. I think this work is interesting and is probably useful for the researchers studying bacterial genetics to follow their protocol. I have several comments to be addressed before publication.

1. My first impression is that all data are graphs representing genome editing efficiency and none of the raw data is shown. I think actual experimental data before calculations of the editing efficiency should be shown as supplemental data. In addition, I want to see the colonies appearing as Ref resistance on the agar plate from sbcC mutant as compared with that from the wild type. Please see the picture of a representative agar plate.

2. The authors selected 129 mutants of the repair gene from the Keio mutant library, and found that the sbcC mutant was critical for increasing prime editing efficiency. I think the mutant strains selected in this study should be listed up in the manuscript, at least in the supplemental data.

3. Their conclusion insists that ExoI (the sbcC product) is the only critical nuclease for the prime editing and ExoVII (the xseA product) and ExoX (the exoX product) can assist the critical function of ExoI in its absence. Deletion of xseA or exoX by theirselves does not affect the prime editing efficiency. From these results, the authors proposed the 3'-directed hydrolysis model. I think additional experiments are needed to make this model credible. The 3' -flapped DNA seems to be

much more preferable substrate for ExoI as compared with ExoVII and ExoX. This substrate specificity can be confirmed by in vitro assays. Otherwise, do they have any other idea to explain the difference of three 3'-5' exonucleases?

4. A result shown in Fig. 2g is different from others. Deletion of one more gene from xseA and exoX, in addition to sbcC deletion is not different from deletions of all three genes. I understand the locus dependence of the prime editing. However, the result of Fig.2g shows a completely different characteristics from other loci, and this result affects on the conclusion of this study. The authors should adequately address this issue.

5. Regarding to the result shown in Fig. 4a, the difference of the efficiency by BacPE varies from1% to 89.4%. I think the results are too variable, and therefore, the practicality and versatility of BacPE will be questionable, if it is true.

We thank the reviewers for their critical and insightful comments. We have revised the main text per the reviewers' suggestions, which are highlighted in red in the revised manuscript. The following are our point-by-point responses to the reviewers' comments:

Reviewer 1

In this manuscript, the authors explored the applications of prime editing in bacteria. Specifically, through screening 129 Keio collection of *E. coli* mutants, they identified that 3'-5' DNA exonucleases *sbcB*, *xseA*, and *exoX* are the key factors that inhibit prime editing in several bacterial species. Consistently, deleting these genes or repressing their expression through CRISPRi enabled prime editing in several bacterial strains. Based on this, a 3'-directed hydrolysis model for inhibiting prime editing in bacteria was proposed. This work is original and impressive. I have a few concerns.

Response: We deeply appreciate your positive feedback, recognition of this work, and support in the publication of this study.

Major points:

1. There have been various CRISPR-Cas9, Cas12, and native type I Cascade-mediated genome editing established in prokaryotes. How necessary is it to develop prime editing in bacteria? This point was not well justified in the manuscript.

Response: Precise genetic manipulation is vital to studying bacterial physiology, but is difficult to achieve in some bacterial species due to the weak intrinsic homologous recombination (HR) capacity and the lack of a compatible exogenous HR system. Despite the versatility of nuclease-based genome editing methods in bacteria, their applications for genome editing in bacteria require HR for repairing the DSBs after genome cleavage. Prime editing might provide a new genome editing method for bacteria species that lack strong HR systems.

2. Related to point 1, are there bacterial species/ strains and/or the types of editing in which existing editing strategies and approaches are incapable of and prime editing could achieve? The authors should show some examples of such applications. Alternatively, as a methodology manuscript, applications of the developed technique in representative Gram-positive and Gram-negative bacterial species should be shown.

Response: Current CRISPR-based methods for genome editing in *Mycobacterium smegmatis* can only generate non-precise mutations (*mBio*, 2020, DOI: 10.1128/mbio.02364-19) or limited types of base substitution (*Frontiers in Genome Editing*, 2021, DOI: 10.3389/fgeed.2021.734436. *Engineering*, 2022, DOI: https://doi.org/10.1016/j.eng.2022.02.013), we prove that prime editing can introduce point mutation, insertion and deletion edit in *Mycobacterium smegmatis* (Fig. 1a). We envision that the prime editing strategy is applicable for genome editing in *Mycobacterium tuberculosis*. However, operating of *Mycobacterium tuberculosis* requires

a P3 laboratory, which is beyond our experimental conditions. The limitation of this study is how to inhibit the repair pathways that can restrict prime editing efficiency in wild-type bacteria. We have addressed this problem in *E. coli*. For gram-positive bacterium, however, we have not established a robust tool to inhibit the repair pathways that can restrict prime editing efficiency.

3. The authors proposed a 3'-directed hydrolysis model for inhibiting prime editing in bacteria just based on the fact that deletion of 3'-5' exonuclease genes improved prime editing. It would better to provide more direct, biochemical evidence for this mechanism.

Response: Previous studies indicated that ExoI degrades DNA with the rate up to 10,000 nucleotides/min (*Journal of Biological Chemistry*, 1972, DOI: https://doi.org/10.1016/S0021-9258(18)62758-9), substantially faster than that of ExoX, which degrades DNA with the rate up to 1,400 nucleotides/min (*Journal of Biological Chemistry*, 1999, DOI: 10.1074/jbc.274.42.30094). We cannot find the substrate degradation rate of ExoVII. Thereby, we performed the cleavage assay to compare the degradation activity of ExoI and ExoVII on PE intermediates.

PE intermediates were produced by annealing the oligonucleotides depicted in the below figure, further digested with ExoI or ExoVII, and analyzed by denaturing Urea-PAGE. The results showed that both ExoI and ExoVII could degrade the FAM-labeled DNA, but the catalytic rate of ExoI was faster than that of ExoVII. A ~20 nt DNA product could be observed in the degradation assay, and prolonged incubation could result in oligonucleotides shorter than 10 nt, suggesting that both nucleases could also degrade dsDNA.

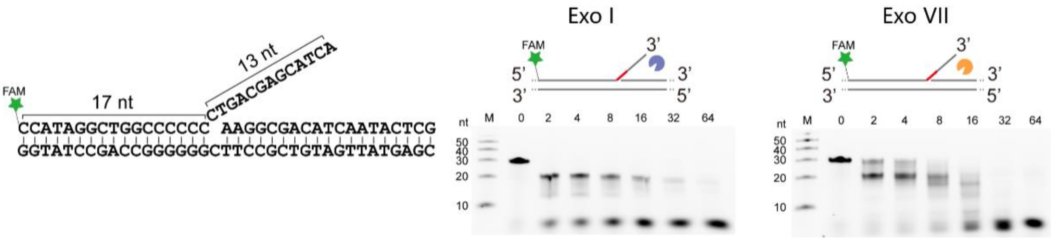

Response Fig. 1 Biochemical evidence for 3'-directed hydrolysis of PE intermediates.
In vitro DNA degradation results for FAM-labeled PE intermediates by exonucleases. The annealed oligonucleotides were prepared as the PE intermediates. The cleaved products were analyzed by TBE-Urea-PAGE.

Minor points:
1. Authors should provide schematic diagrams for the types of editing mentioned in the manuscript, e.g. in the Figure 1, schematic diagrams for the types of editing as depicted in the X- axises should be provided.

Response: Thank you for the suggestion. The schematic diagrams for the types of editing (point mutation, insertion, and deletion) have been shown in Fig. 1.

2. Overall, the figure legends are too simple. Some necessary details should be provided in the legends so that readers can understand the figures with necessary details without going through the materials and methods section.

Response: Thank you for the suggestion. We have provided more details in the legends to make the figures easier to understand in the revised manuscript.

3. What does MMR represent? It should be spelled the first time it appears in the text.

Response: We have provided the definition of MMR the first time it appears in the text (Line 141).

4. Legend of Fig 2 c-g, " in at different targeting loci"

Response: We have revised the sentence as "Comparison of the prime editing efficiency in *E. coli* MG1655 at different targeting loci" in the revised manuscript (Line 588).

Reviewer #2:

In this work, Zhang and colleagues show that repressing or deleting exonucleases involved in DNA repair can radically boost prime editing in bacteria. After finding that prime editing is highly efficient in M. smegmatis, they began screening for DNA repair factors potentially accounting for poor editing in E. coli. This screen uncovered sbcB, a 3'-to-5' DNA exonuclease, whose deletion boosted prime editing in E. coli across target sites. Further deleting two other known DNA endonucleases (xseA, exoX) further boosted prime editing. Engineering the pegRNA based on prior work further enhanced prime editing, in some cases exceeding 80%. Adopting CRISPRi to repress these three genes allowed enhanced editing in a WT strain of E. coli, while deleting some of these genes in Klebsiella and Acinetobacter also enhanced prime editing across sites. Finally, the authors present a model of how these exonucleases interfere with prime editing.
To my knowledge, prime editing in bacteria has only been reported in one publication (Tong et al., Nat Commun 2021), but with poor editing efficiencies at genomic sites, hindering its adoption by the bacterial community. This work makes an important advance by identifying and removing the responsible barriers, which could open up broad use of prime editing in bacteria and open a range of editing options for fundamental research and strain engineering. The authors further provide extensive datasets, and the manuscript was well-written and should be easily accessible to a broad audience. I do have some important comments about the toxicity/escape as well as claims in the abstract and reproducibility, although I see these only strengthening the work.

Response: We really appreciate the reviewer's recognition of this work.

Major comments:

1. While the authors provide extensive editing data, the impact of CFUs compared to non-targeting conditions would be incredibly important to report. Targeting, particularly in the absence of repair proteins, could be highly cytotoxic, with few surviving cells. Such a condition would also select for escape phenotypes such as disruption of the Cas9 or the pegRNA or an unintended edit at the target site. While I don’t expect the authors to provide this information for all target sites, reporting relative CFUs and looking at whether non-edited cells are escapers for a few representative examples would be incredibly important to potential users.

Response: We performed prime editing at the *xylB* loci using BacPE system according to protocols listed in methods section. The results showed that targeting *xylB* slightly decreased the CFU compared with the non-targeting control (a), and we speculate that the inhibition of repair proteins might be cytotoxic to *E. coli*. We randomly picked 24 colonies and PCR amplified the targeting region for Sanger sequencing. All the colonies contained successful editing and no escapers were observed in the assay (b). Moreover, we sequenced the editing plasmids, and no mutations occurred in both the PE nuclease or the pegRNA.

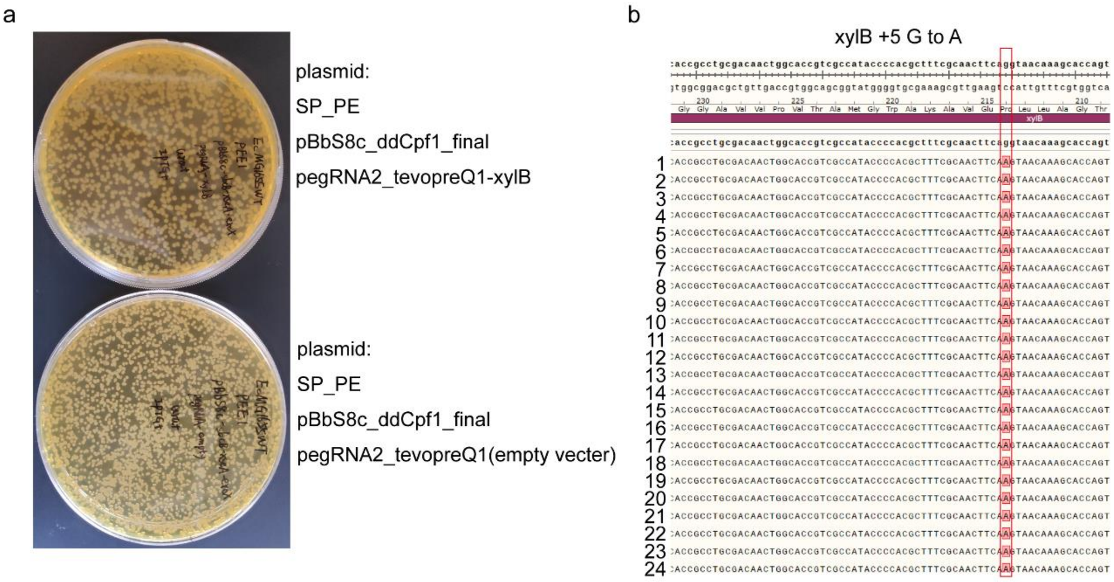

Response Fig. 2 Cytotoxic assay of the BacPE system in *E. coli* MG1655. a, CFUs with the BacPE system after editing at the *xylB* site. 10 ng pegRNA plasmids were electroporated into *E. coli* MG1655 harboring the PE effecter plasmid and the CRISPRi plasmid. In the top plate, prime editing with the BacPE system at *xylB*; in the bottom plate, the BacPE system has no target on the genome. b Sequence alignments of the targeted loci. 24 colonies were randomly picked, and the *xylB* loci were amplified and sequenced. The colony numbers are shown on the left of the sequences. The mutated bases are highlighted in red.

2. While the main text generally contains conclusions that nicely match the presented data, the abstract takes a number of liberties that need to be rephrased. Specifically,

- “Here, we report….”: inhibition was only shown for *E. coli*, so this should indicate inhibition or deletion.

Response: We have revised the sentence as “Herein, we report the development of a versatile prime editing platform in *E. coli* (termed BacPE) by inhibiting 3′ →5′ DNA exonucleases.” in the revised manuscript (Line 41).

- “Comparative prime editing….”: The comparison was only between M. smegmatis and E. coli, so better to state these two species.

Response: We have revised the sentence as “Comparative prime editing in *M. smegmatis* and *E. coli* identified that the bacterial genetic background is likely a key factor in restricting efficient prime editing.” in the revised manuscript (Line 43).

- “Genetic screening of….”: It would be more pertinent to state the number of repair genes that were screened rather than the number of transposons tested. If the number is smaller, then it’s reasonable to drop the specific number.

Response: We have revised the sentence as “Genetic screening of 129 potential DNA repair-related genes using *Escherichia coli* transposon mutants identified *sbcB*.” in the revised manuscript (Line 45).

- “We propose a 3’-directed….”: I would recommend rewording this to indicate that disrupting related exonucleases enhanced prime editing rather than claiming the model holds. Otherwise, there is a much higher bar to claim a mechanistic model spanning different bacterial strains.

Response: We have revised the sentence as “We demonstrated that disrupting related exonucleases enhanced prime editing in *E. coli* and other bacterial species.” in the revised manuscript (Line 50).

- “Efficient prime editing can be achieved….”: CRISPRi was only shown in E. coli, so it remains to be shown that this same approach can greatly boost prime editing in other bacteria.

Response: We have revised the sentence as “Efficient prime editing in wild-type *E. coli* can be achieved.” in the revised manuscript (Line 52).

3. I could not find any description of the number of replicates or the nature of the replicates. In most cases, the error bars were extremely small, raising some concerns how independent the replicates were and how reproducible the overall results are.

Response: We listed the number of replicates in the figure legends in the revised manuscript.

4. The authors note that the ddCas12a silencing construct represses the target genes by ~80% individually. How much is repression when the genes are combined? Individual values normally decrease as multiplexing increases.

Response: We assessed the repression efficiency by RT-qPCR in the revised manuscript (Supplementary Figure 10). In the BacPE system, multiple crRNA expression units were assembled into a single plasmid and the repression efficiency of *sbcB*, *xseA* and *exoX* was 57.4%, 88.5% and 76.3%, respectively. A slight decrease in repression efficiency was observed for *xseA* when multiple crRNAs were expressed simultaneously, whereas no significant decrease in repression efficiency for *sbcB* and *exoX* when multiple crRNAs were expressed, likely because the crRNAs were transcribed using separated promoters, rather than transcribed in a crRNA array driven by a single promoter.

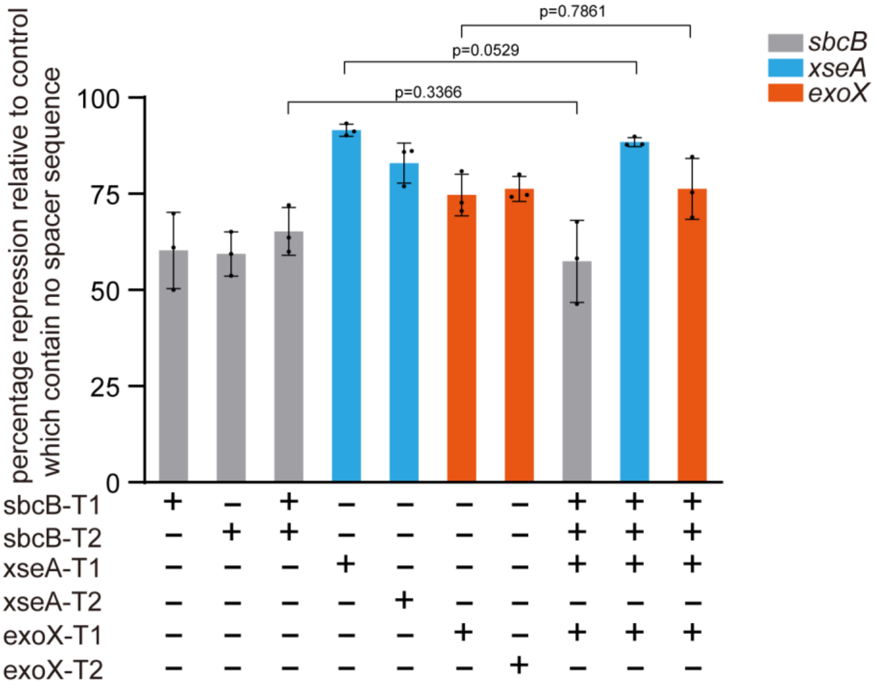

Response Fig. 3 CRISPRi-mediated repression of gene expression with different spacers. Gene-specific spacers were designed to target *scbB*, *xseA* or *exoX*. In the BacPE system, different crRNAs were assembled into a single plasmid to inhibit *sbcB*, *xseA* and *exoX* simultaneously. Student's *t*-test was performed. Data represent mean ± s.d. of n = 3 independent replicates.

Other comments:

5. L. 73-77: the original demonstration of prime editing in *E. coli* was able to achieve chromosomal editing, albeit at very low levels. Therefore, some rewording is needed here to better capture what was previously demonstrated.

Response: We have revised the sentence as "applying prime editors in prokaryotes are limited to *Escherichia coli* and the editing activities are at low levels" in the revised manuscript (Line 88).

6. L. 118: For comparisons of the WT and ΔmutS (Ext. Data Fig. 6), the authors write that mutS deletions have minimal or no impact on the editing efficiency, but at least for the adhE target site it looks like it could have an effect. Including statistical tests with the corresponding p-values would be helpful.

Response: The p-values were noted in the revised manuscript. We revised "However, deletion of *mutS* had minimal or no impact on the editing efficiencies" as "In most cases (40/45), deletion of *mutS* had minimal or no impact on the improvement of prime editing efficiency" in the revised manuscript (Line 148).

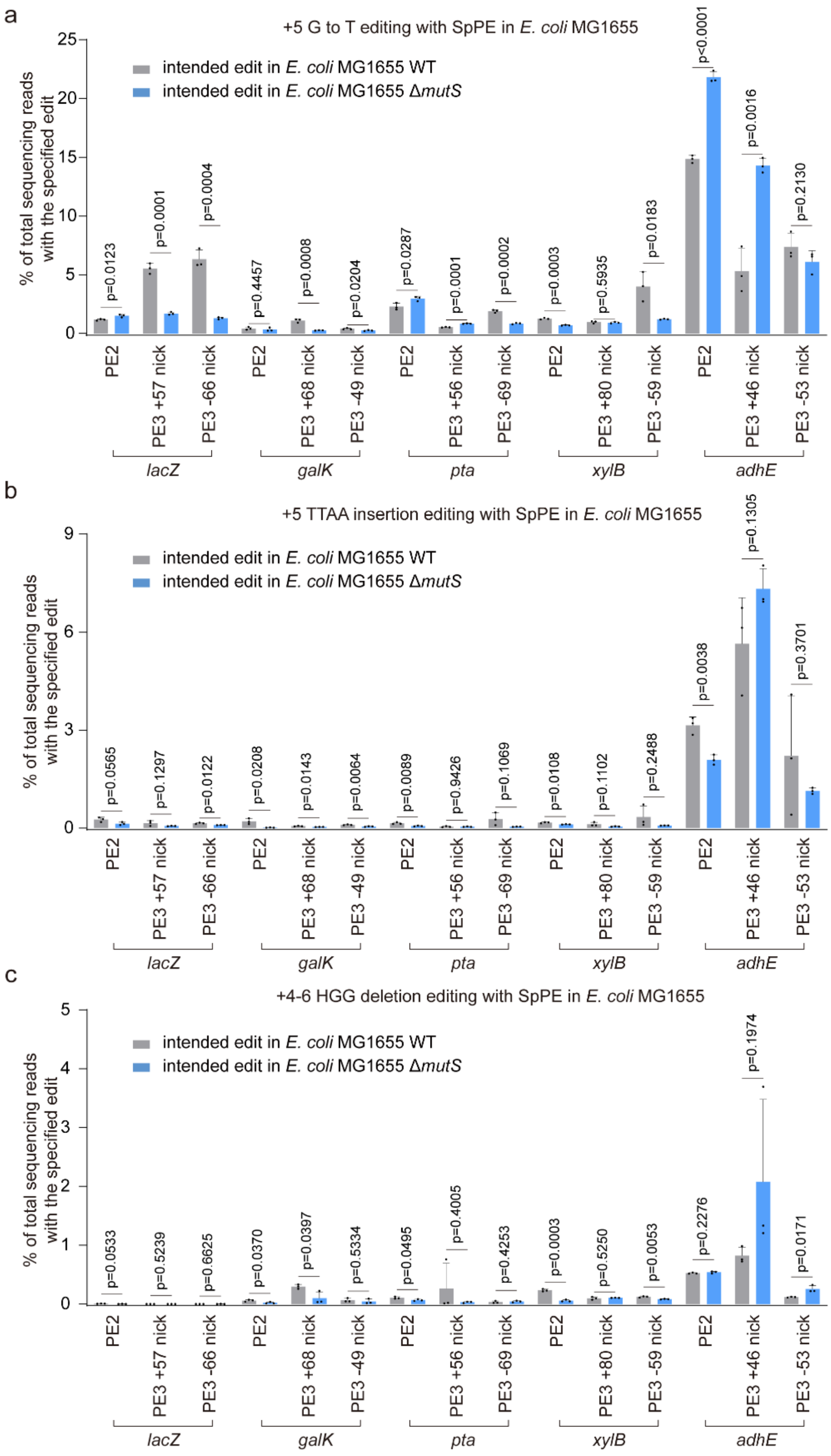

Response Fig. 4 The effect of MMR on prime editing efficiency in *E. coli*. a +5 G to T editing efficiency in the WT strain and MMR-deficient strain. b +5 TTAA insertion editing efficiency in the WT strain and MMR-deficient strain. c +4-6 HGG deletion editing efficiency in the WT strain and MMR-deficient strain. H represents A, C or T. Student's *t*-test was performed. Data represent mean ± s.d. of n = 3 independent replicates.

7. L. 122: how many genes were screened as part of the transposon set? It would also be helpful for these genes to be listed in the SI.

Response: 129 genes were screened in genetic screening approach, and these genes were listed in Supplementary Table 1 in the revised manuscript.

8. L. 128-130: The transition from the prior sentence is abrupt. I recommend first stating that sbcB emerged as the top hit in the screens.

Response: We added "We observed that *sbcB* emerged as the top hit in the screens" in the revised manuscript for transition (Line 159).

9. L. 134: For comparisons of the contribution of single- or combined knockouts of the 3′ → 5′ -DNA exonucleases on the prime editing efficiency (e.g., Fig. 2c-g), are the differences significant from the WT values? Here the authors could include statistical tests and add the p-values to the main text.

Response: The p-values between knock out mutants and WT are noted in the revised manuscript.

10. L. 139: Which genes were deleted? Listing these here or in the SI would be helpful.

Response: The deleted genes were listed in Supplementary Table 1 in the revised manuscript.

11. L. 147: Looking at Fig. S9, the edit made to xylB yields increased editing when sbcB and other repair genes were deleted. Why is there an exception, and can the authors reword the sentence to capture this?

Response: We have replaced "Intriguingly, except *xseA* or *exoX,* the additional deletion of other potential 3′ →5′ DNA exonuleases in the *sbcB* mutant did not enhance the editing efficiency" with "Except the *xylB* loci, the additional deletion of other potential 3′ →5′ DNA exonuleases in the *sbcB* mutant did not enhance the editing efficiency" in the revised manuscript (Line177).

12. L. 210: Replace "significant" with "substantial" since no statistical comparisons are being made.

Response: We have replaced "significant" with "substantial" in the revised manuscript (Line 248).

13. Fig 2a: Write genes following standard nomenclature for bacteria. Also change "scrap" to "scrape".

Response: We have corrected genes following standard nomenclature for bacteria and changed “scrap” to “scrape” in the revised manuscript.

14. Fig. 2b: based on the depicted assay, the axes should indicate the colony ratio rather than the mutation occurrence, as the authors did not directly assess the frequency of this edit.

Response: We have replaced “D516Y mutation occurrence in rpoB” with “The ratio of Rif resistant clones/total clones” in the revised manuscript.

15. Fig. 2c-g: what are the colored bars meant to represent?

Response: We have identified three genes that inhibit prime editing, and combinational deletion of these genes can improve prime editing efficiency, and thus, we highlighted those single and combinational deletion mutants so that readers can capture this information more easily. We realized that Fig. 2c-g need more description, and we added “Δ*sbcB*, Δ*sbcB*Δ*xseA*, and Δ*sbcB*Δ*exoX* mutants are colored in blue, Δ*sbcB*Δ*xseA*Δ*exoX* mutants are colored in red.” in the revised manuscript.

16. Fig. 2h: for the middle arrow, can an intermediate step be added to show gap filling?

Response: The gap filling step has been added to the ligation step in the revised manuscript.

17. I recommend making the plasmids available on Addgene upon publication of the work to ensure the approach can be broadly disseminated. Providing annotated plasmid maps for key constructs such as through Benchling would also ease adoption.

Response: Plasmids have been deposited on Addgene, and will be available after quality control.

18. The amplicon sequencing data should be made publicly available.

Response: The amplicon sequencing data have been uploaded to NCBI and will be released after publication.

19. The authors could discuss in more detail the importance of investigating redundancy of gene functions in repair pathways as important finding. The authors could also provide a short statement on how prime editing in the field of microbiology could be of advantage in comparison to already established CRISPR-Cas gene editing technologies.

Response: Our findings highlight the importance of intrinsic pathways and redundant genes on prime editing restriction, and might provide insights on improving other genome editing tools. Nuclease-based genome editing methods rely on intrinsic or

exogenous homologous recombination (HR) systems for DSB repair. Modulation of cellular pathways that can enhance HR capacity would improve the editing efficiencies of those technologies. CRISPR-Cas12k-associated transposons (CASTs) can mediate site-specific DNA-insertion in bacteria. However, the DNA insertion efficiencies vary substantially across different bacterial species (*Science*, 2019, https://doi.org/10.1126/science.aax9181, *Cell Reports*, 2021, https://doi.org/10.1016/j.celrep.2021.109635). The discovery of cellular determinants would also improve the editing efficiency of CRISPR-Cas12k-mediated DNA insertion.

Compared with nuclease-based genome editing methods that rely on HR for precise editing, prime editors achieve the installation of any single base substitution and small insertions and deletions without requiring homologous recombination or double-strand DNA breaks, potentiating the editing in bacterial species that lack a strong HR system.

Reviewer #3:

This manuscript written by Zhang H et al describes the development of a practical prime editing method, which is useful for bacterial fine genome editing. The authors wanted to provide a useful prime editing method for E. coli and found that Exonuclease I (the sbcC gene product) is critical for reducing the efficiency in E. coli cells. Furthermore, additional mutations of xseA and exoX drastically enhanced the prime editing efficiency (up to 100-fold). From these experimental results, the authors proposed a model of the 3'-directed hydrolysis for degradation of the prime editing intermediates to explain inhibition of prime editing in Bacteria. I think this work is interesting and is probably useful for the researchers studying bacterial genetics to follow their protocol. I have several comments to be addressed before publication.

Response: We really appreciate the reviewer's recognition of this work.

1. My first impression is that all data are graphs representing genome editing efficiency and none of the raw data is shown. I think actual experimental data before calculations of the editing efficiency should be shown as supplemental data. In addition, I want to see the colonies appearing as Ref resistance on the agar plate from sbcC mutant as compared with that from the wild type. Please see the picture of a representative agar plate.

Response: The raw data has been listed in Source data in the revised manuscript. After prime editing, the WT strain and the *sbcB* mutant strain were plated onto agar plates. For each plate, all colonies were scraped and diluted, and then equal volume of cells (10 uL) were plated onto agar plates with or without rifampin. The ratio of Rif resistant clones/total clones were calculated as described below. The picture of a representative agar plate was shown below.

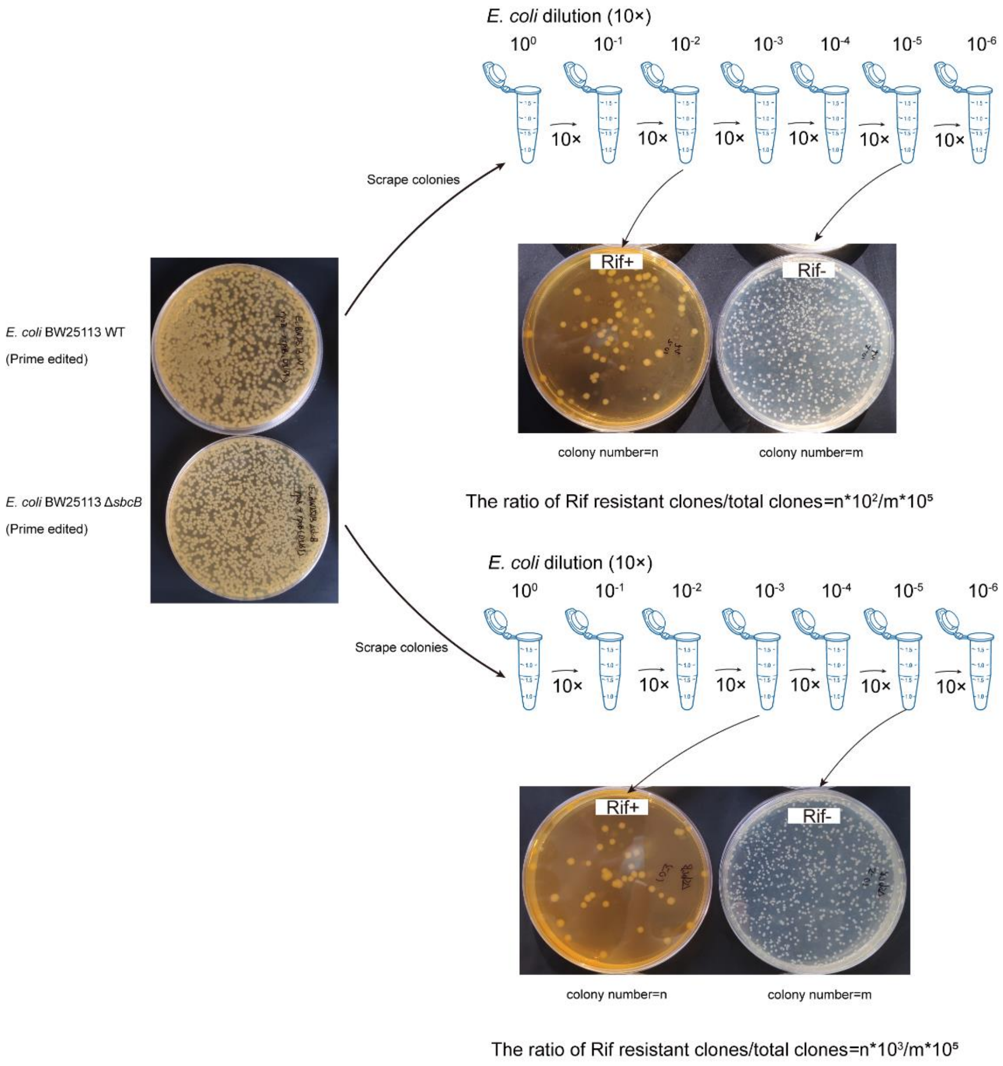

Response Fig. 5 The pictures of representative agar plates in the *rpoB* assay. *E. coli* BW25113 was prime edited and plated onto an agar plate without rifampin. After incubation at 37℃ for 18 hours, all cells were scraped and diluted in EP tubes. Cells were 10×diluted, and 10 uL cells were plated onto agar plates supplied with or without rifampin.

2. The authors selected 129 mutants of the repair gene from the Keio mutant library, and found that the sbcC mutant was critical for increasing prime editing efficiency. I think the mutant strains selected in this study should be listed up in the manuscript, at least in the supplemental data.

Response: The selected 129 mutants from Keio collection have been listed in Supplementary Table 1 in the revised manuscript.

3. Their conclusion insists that ExoI (the sbcC product) is the only critical nuclease for the prime editing and ExoVII (the xseA product) and ExoX (the exoX product) can assist the critical function of ExoI in its absence. Deletion of xseA or exoX by theirselves does not

affect the prime editing efficiency. From these results, the authors proposed the 3'-directed hydrolysis model. I think additional experiments are needed to make this model credible. The 3' -flapped DNA seems to be much more preferable substrate for ExoI as compared with ExoVII and ExoX. This substrate specificity can be confirmed by in vitro assays. Otherwise, do they have any other idea to explain the difference of three 3'-5' exonucleases?

Response: Previous studies indicated that ExoI degrades DNA with the rate up to 10,000 nucleotides/min (*Journal of Biological Chemistry*, 1972, DOI: https://doi.org/10.1016/S0021-9258(18)62758-9), substantially faster than that of ExoX, which degrades DNA with the rate up to 1,400 nucleotides/min (*Journal of Biological Chemistry*, 1999, DOI: 10.1074/jbc.274.42.30094). We cannot find the substrate degradation rate of ExoVII. Thereby, we performed the cleavage assay to compare the degradation activity of ExoI and ExoVII on PE intermediates.

PE intermediates were produced by annealing the oligonucleotides depicted in the below figure, further digested with ExoI or ExoVII, and analyzed by denaturing Urea-PAGE. The results showed that both ExoI and ExoVII could degrade the FAM-labelled DNA, but the catalytic rate of ExoI was faster than that of ExoVII. A ~20 nt DNA product could be observed in the degradation assay, and prolonged incubation could result in oligonucleotides shorter than 10 nt, suggesting that both nucleases could also degrade dsDNA.

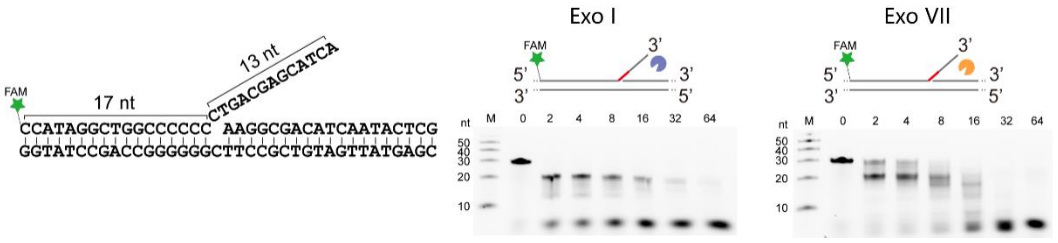

Response Fig. 1 Biochemical evidence for 3'-directed hydrolysis of PE intermediates.
In vitro DNA degradation results for FAM-labeled PE intermediates by exonucleases. The annealed oligonucleotides were prepared as the PE intermediates. The cleaved products were analyzed by TBE-Urea-PAGE.

4. A result shown in Fig. 2g is different from others. Deletion of one more gene from xseA and exoX, in addition to sbcC deletion is not different from deletions of all three genes. I understand the locus dependence of the prime editing. However, the result of Fig.2g shows a completely different characteristics from other loci, and this result affects on the conclusion of this study. The authors should adequately address this issue.

Response: In Fig. 2g, we noticed that the deletion of *sbcB* already achieved a high editing efficiency of ~50%, whereas the deletion of *sbcB* could only achieve the efficiencies of < 20% in Fig. 2c-f. The further enhancement of the editing efficiency at this editing type in Fig. 2g may be restricted by other mechanisms, but not by the redundant exonucleases. Improvements in the PE machinery, such as using the evolved reverse transcriptase

(Cell, 2023, https://doi.org/10.1016/j.cell.2023.07.039), or screening other restrictive pathways, may further enhance the editing efficiencies.

5. Regarding to the result shown in Fig. 4a, the difference of the efficiency by BacPE varies from1% to 89.4%. I think the results are too variable, and therefore, the practicality and versatility of BacPE will be questionable, if it is true.

Response: Prime editing efficiencies can vary substantially on cell types, editing types, PBS lengths, RTT lengths and other factors, and it requires substantial efforts and resources to determine the most efficient pegRNAs to generate a desired edit under various experimental conditions (*Cell*, 2023, DOI: https://doi.org/10.1016/j.cell.2023.03.034). Recent studies have developed computational models to facilitate prime editing applications in human cells. We speculate that similar computational models that facilitate pegRNA design may improve the versatility of BacPE in bacteria.

Reviewers' Comments:

Reviewer #1:
Remarks to the Author:
The authors have addressed my comments adequately.

Reviewer #2:
Remarks to the Author:
The authors have sufficiently addressed all comments raised by the reviewers.

REVIEWERS' COMMENTS

Reviewer #1 (Remarks to the Author):

The authors have addressed my comments adequately.

Response: We deeply appreciate your positive feedback, recognition of this work, and support in the publication of this study.

Reviewer #2 (Remarks to the Author):

The authors have sufficiently addressed all comments raised by the reviewers.
Response: We deeply appreciate your positive feedback, recognition of this work, and support in the publication of this study.
